# Genomic Expression Profiling and Bioinformatics Analysis of Chronic Recurrent Multifocal Osteomyelitis

**DOI:** 10.1155/2021/6898093

**Published:** 2021-02-09

**Authors:** Kai Huang, Bingyuan Lin, Yiyang Liu, Qiaofeng Guo, Haiyong Ren

**Affiliations:** Department of Orthopaedics, Tongde Hospital of Zhejiang Province, Hangzhou, China

## Abstract

**Objective:**

Chronic nonbacterial osteomyelitis (CNO) is an autoinflammatory bone disorder. Its most severe form is referred to as chronic recurrent multifocal osteomyelitis (CRMO). Currently, the exact molecular pathophysiology of CNO/CRMO remains unknown. No uniform diagnostic standard and treatment protocol were available for this disease. The aim of this study was to identify the differentially expressed genes (DEGs) in CRMO tissues compared to normal control tissues to investigate the mechanisms of CRMO.

**Materials:**

Microarray data from the GSE133378 (12 CRMO and 148 matched normal tissue samples) data sets were downloaded from the Gene Expression Omnibus (GEO) database. DEGs were identified using the limma package in the R software. Gene Ontology (GO) analysis, Kyoto Encyclopedia of Genes and Genomes (KEGG) pathway analysis, and protein-protein interaction (PPI) network analysis were performed to further investigate the function of the identified DEGs.

**Results:**

This study identified a total of 1299 differentially expressed mRNAs, including1177 upregulated genes and 122 downregulated genes, between CRMO and matched normal tissue samples. GO analyses showed that DEGs were enriched in immune-related terms. KEGG pathway enrichment analyses showed that the DEGs were mainly related to oxidative phosphorylation, ribosome, and Parkinson disease. Eight modules were extracted from the gene expression network, including one module constituted with immune-related genes and one module constituted with ribosomal-related genes.

**Conclusion:**

Oxidative phosphorylation, ribosome, and Parkinson disease pathways were significantly associated with CRMO. The immune-related genes including IRF5, OAS3, and HLA-A, as well as numerous ribosomal-related genes, might be implicated in the pathogenesis of CRMO. The identification of these genes may contribute to the development of early diagnostic tools, prognostic markers, or therapeutic targets in CRMO.

## 1. Introduction

Chronic nonbacterial osteomyelitis (CNO) is an autoinflammatory bone disorder that primarily affects children and adults [1]. It can generally occur in all age groups while the peak age-of-onset was between 7 and 12 years [2]. The clinical presentation of CNO varies widely, from mild, time-limited, and unifocal asymptomatic bone lesions up to severe, multifocal bone lesions with chronically active or recurrent features [3, 4]. These severe manifestations are referred to as chronic recurrent multifocal osteomyelitis (CRMO) [5].

Despite the intense scientific efforts that had contributed to a better understanding of the underlying molecular mechanism of CNO/CRMO, the exact molecular pathophysiology remains unknown [2, 6]. An infectious origin is excluded as no apparent infectious agents are detectable at the site of the bone lesion, and antibiotic treatment does not affect clinical signs or patient symptoms. It is suggested that CRMO can be genetically driven. It has also been suggested that CRMO may constitute a pediatric form of SAPHO (synovitis, acne, pustulosis, hyperostosis, and osteitis) syndrome [7, 8]. Findings indicated that an imbalance between proinflammatory (TNF-*α*, IL-6) and anti-inflammatory (IL-10, IL-1*β*) cytokines may be centrally involved in the molecular pathology of CNO [9, 10]. Moreover, mutations in IL1RN, LPIN2, FBLIM1, and Pstpip2 have been found in human or murine models of CRMO [6].

There are no widely accepted diagnostic criteria or disease biomarkers; CNO/CRMO remains a diagnosis of exclusion [1]. As the clinical presentation is greatly variable and the clinical symptoms were commonly unspecific, the diagnosis is frequently delayed or even missed [11]. Misdiagnosis, prolonged antibiotic treatment, and delays in optimal treatment were unavoidably persistent among the patients. The estimated prevalence of CNO/CRMO is reported to be 1-2 per million [12]. Although CNO is still deemed as a rare disorder, it is believed that the incidence would be underestimated [10]. According to several case series, CNO/CRMO may be almost as common as infectious osteomyelitis [1, 13, 14]. Currently, in the absence of an exact treatment protocol for patients with CNO/CRMO, treatment of this disease is largely based on expert opinion, case reports, and also personal experience [1, 10]. Nonsteroidal anti-inflammatory drugs (NSAIDs) are usually deemed as the first-line treatment, which have been demonstrated to be useful for pain control and inducing remission [10]. Corticosteroids, disease-modifying antirheumatic drugs (such as methotrexate or sulfasalazine), anti-TNF agents, or bisphosphonates had also been reported to be effective [1, 3]. However, longitudinal, placebo-control large-scale studies on the different possible therapeutic strategies are absent, and the molecular pathophysiology of CNO/CRMO remains incompletely understood; the optimal treatment strategy for CNO/CRMO remains difficult to determine. The discovery of gene network interactions could provide important insights to advance our understanding of the pathogenesis of CNO/CRMO, and the analysis of abnormal gene expression in the patients would help explore new molecular targets for development of diagnostic tools or drugs against CNO/CRMO.

## 2. Materials and Methods

### 2.1. Collection of Gene Expression Data Sets

Microarray data from the GSE133378 data sets (https://www.ncbi.nlm.nih.gov/geo/query/acc.cgi?acc=GSE133378) were downloaded from the GEO (https://www.ncbi.nlm.nih.gov/geo) of NCBI. All data were freely accessible online. GSE133378 includes 49 viral and 7 bacterial cases. Three CRMO samples (R_BEN-012_S19_L001, R_UZG_6_S27_L001, and R_UZG_4_S25_L001) with four biological repetitions and three control samples (AZT14C_S38_L001, AZT15C_S40_L001, and AZT1C_S20_L001) were randomly selected from 148 control samples. The relationship of the intersamples was analyzedThe inter-relationship of the samples was analyzed by principal component analysis (PCA). The array Quality package was used for quality control, and the limma package was used to apply raw data in R software. The normalization criteria were quantile normalization. Genes that met fold changes (FC) > 1 and false discovery rate (FDR) < 0.05 were selected as differentially expressed genes (DEGs).

### 2.2. GO Term and KEGG Pathway Enrichment Analyses

To conduct enrichment analyses, the package clusterProfiler (version 3.8.1) of R software (version 3.4.0) was used for analyzing the KEGG pathways and GO processes in this study. The identified DEGs were enriched for terms in the GO biological process (BP), molecular function (MF), and cellular component (CC) categories [15]. KEGG pathway analysis was utilized to clarify the potential functions and signaling pathways of the DEGs.

### 2.3. Protein-Protein Interaction (PPI) Network Analysis

The Search Tool for the Retrieval of Interacting Genes/Proteins (STRING, https://stringdb.org/) was used to generate the PPI network of proteins encoded by the DEGs. A combined score > 0 was selected to construct the coexpression PPI network. The PPI network was constructed by the Cytoscape Software 3.8 (http://cytoscape.org/).

## 3. Results

### 3.1. Identification of 1299 DEGs in CRMO

**Figure 1:**
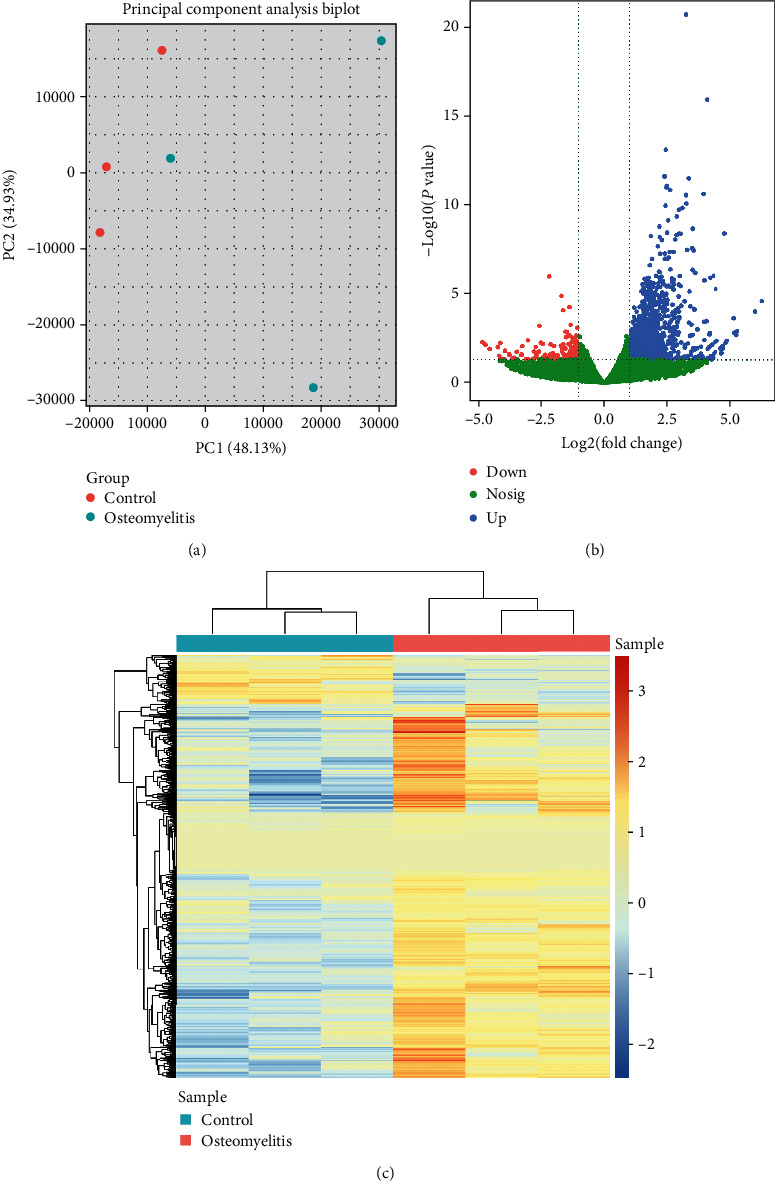
Identification of 1299 DEGs in CRMO. (a) The plot of PCA analysis between CRMO samples and control samples after normalized gene expression. (b) Volcano plots of the gene expression between CRMO samples and control samples from GSE133378 data sets. Cutoff: ∣log2(FC) | >1, *P* value < 0.05. The blue dots represent upregulated DEGs, the red dots represent downregulated DEGs, and the green dots indicated no statistically significant genes. (c) The heat map of the gene expression between CRMO samples and control samples from GSE133378 data sets. Cutoff: ∣log2(FC) | >1, *P* value < 0.05.

The relationship between CRMO samples and control samples was analyzed by PCA, and the results showed obvious bias between CRMO samples and control samples; moreover, the biological repetition of three CRMO samples was consistent (Supplementary Figure S1). Thus, after the data was normalized (Supplementary Figure S2), three CRMO samples (R_BEN-012_S19_L001, R_UZG_6_S27_L001, and R_UZG_4_S25_L001) and three control samples (AZT14C_S38_L001, AZT15C_S40_L001, and AZT1C_S20_L001) were used for subsequent analyses (Figure 1(a)). By the criteria of FDR < 0.05 and fold change > 1, we identified 1299 mRNAs differentially expressed in CRMO compared with matched normal tissues. As a result, 1177 mRNAs were upregulated, and 122 mRNAs were downregulated (Figures 1(b) and 1(c)). The top 10 DEGs according to the value of log_2_FC are listed in Table 1. The detailed DEGs are summarized in Supplementary File 1.

### 3.2. Analyses of Biological Function

**Figure 2:**
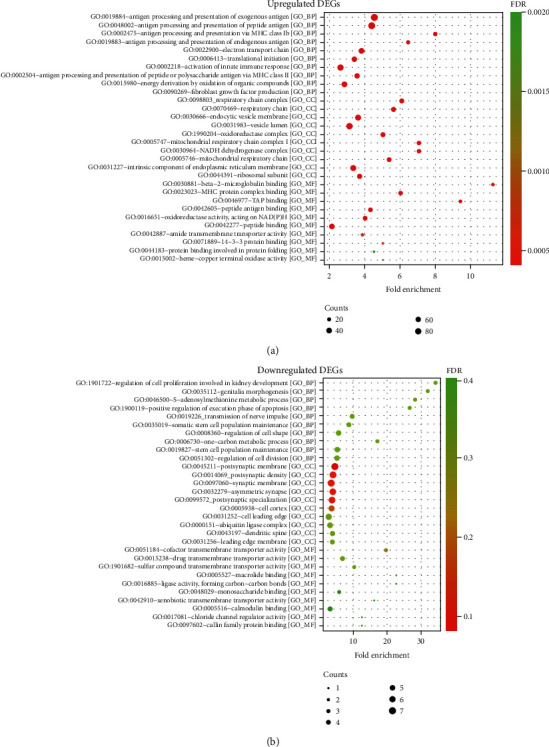
GO function analysis of biological function. (a) The top 30 GO function (BP, CC, and MF) analyses of upregulated DEGs. Cutoff: *P* value < 0.05. (b) The top 30 GO function (BP, CC, and MF) analyses of downregulated DEGs. Cutoff: *P* value < 0.05.

The results of GO term analysis illustrated that in terms of biological processes (BP), the upregulated genes were mainly enriched in “antigen processing and presentation of exogenous antigen,” “antigen processing and presentation of peptide antigen,” and “antigen processing and presentation via MHC class Ib” (Figure 2(a)). Downregulated genes were mainly enriched in “regulation of cell proliferation involved in kidney development,” “genitalia morphogenesis,” and “S-adenosylmethionine metabolic process” (Figure 2(b)). In terms of cellular components (CC), upregulated genes were mainly enriched in the “respiratory chain complex,” “respiratory chain,” and “endocytic vesicle membrane” areas (Figure 2(a)), whereas downregulated genes were mainly enriched in “postsynaptic membrane,” “postsynaptic density,” and “synaptic membrane” (Figure 2(b)). In terms of molecular function (MF), upregulated genes were mainly enriched in “beta-2-microglobulin binding,” “MHC protein complex binding,” and “TAP binding” (Figure 2(a)), whereas downregulated genes were mainly enriched in “cofactor transmembrane transporter activity,” “drug transmembrane transporter activity,” and “sulfur compound transmembrane transporter activity” (Figure 2(b)). As shown in the GO analysis, numerous genes were enriched in GO terms that associated with autoimmune function, such as GO:0019884 antigen processing and presentation of exogenous antigen, GO:0048002 antigen processing and presentation of peptide antigen, GO:0002475 antigen processing and presentation via MHC class Ib, GO:0019883 antigen processing and presentation of endogenous antigen, and GO:0002218 activation of innate immune response. The detailed results of the GO enrichment analyses are provided in Supplementary File 2.

**Figure 3:**
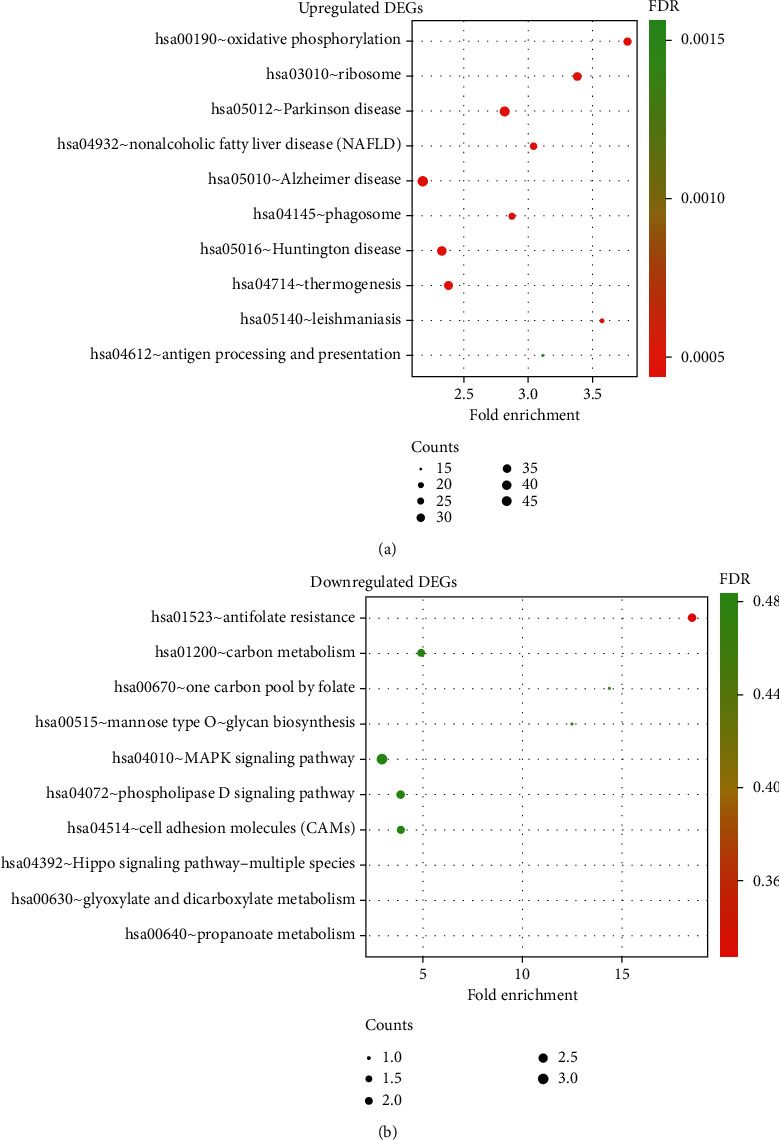
KEGG pathway enrichment analysis of biological function. (a) The top 10 enriched KEGG pathways of upregulated DEGs. Cutoff: *P* value < 0.05. (b) The top 10 enriched KEGG pathways of downregulated DEGs. Cutoff: *P* value < 0.05.

Furthermore, based on KEGG pathway enrichment analysis, the upregulated genes were enriched in pathways such as “oxidative phosphorylation,” “ribosome,” and “Parkinson disease,” whereas downregulated genes were enriched in pathways such as “antifolate resistance,” “carbon metabolism,” and “one carbon pool by folate.” The top 10 enriched pathways of upregulated DEGs and downregulated DEGs are shown in Figure 3. The detailed results of the KEGG pathway analyses are provided in Supplementary File 3.

### 3.3. Establishment of PPI Network

**Figure 4:**
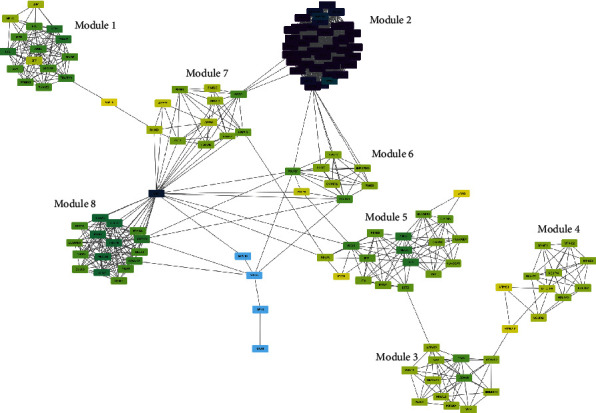
Establishment of PPI network based on DEGs. The PPI network of the DEGs after screening the proteins with degree > 6. The PPI network can be divided into 8 modules.

**Figure 5:**
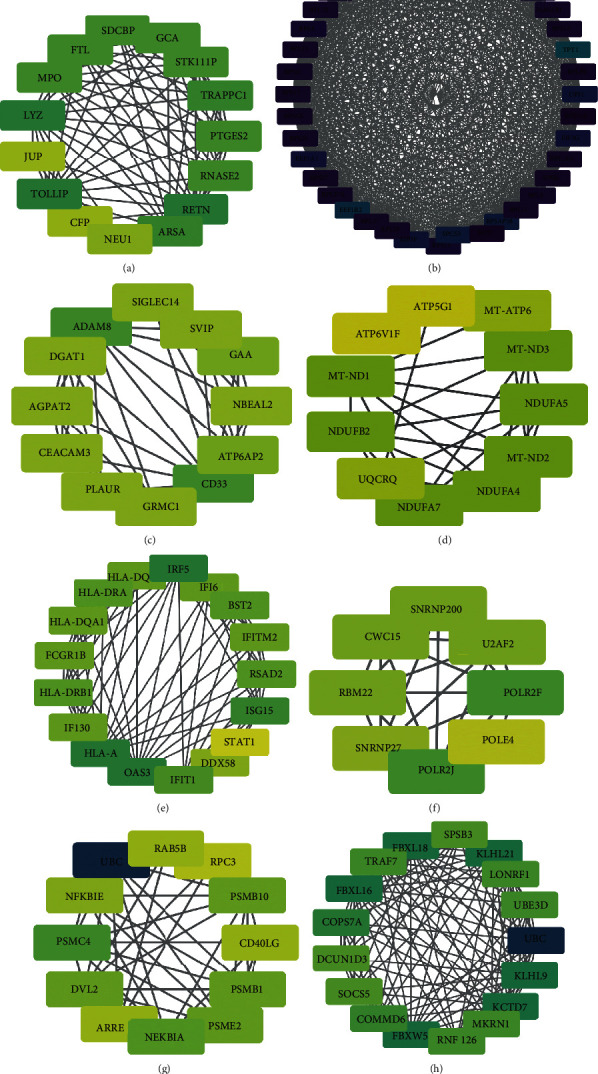
Eight sub-PPI networks of PPI network. (a–h) Eight strong connected sub-PPI networks from the PPI network.

STRING was used to generate the PPI network, which can illustrate interactions between identified DEGs. This PPI network contained 317 nodes and 1432 edges (Supplementary Figure S3). We found numerous DEGs with high connectivity with other proteins. In order to identify protein interaction modules with strong interaction, we screened the proteins with degree > 6 for further analysis. And then a network with 137 nodes and 1165 edges was obtained (Figure 4), while 8 modules were selected from the PPI network by the MCODE plugin in Cytoscape (Figure 5). Further GO term and KEGG pathway analyses for DEGs in each module were performed to clarify the potential functions and signaling pathways of the genes (Supplementary Figure S4 and S5). In module 2 (Figure 5(b)), 40 proteins were found to own high connectivity with other proteins. 31 of 40 proteins encoded ribosomal proteins (RBs) and another 9 proteins including FAU (degree = 38), EIF3G (degree = 33), UPF1 (degree = 32), MAGOH (degree = 38), EIF3F (degree = 32), EEF1B2 (degree = 27), TPT1 (degree = 24), SPCS3 (degree = 31), and EEF1A1 (degree =33). In module 5 (Figure 5(e)), the DEGs were found to be immune-related, including IRF5, IFI6, BST2, IFITM2, RSAD2, ISG15, STAT1, DDX58, IFIT1, OAS3, HLA-A, IFI30, HLA-A, IFI30, HLA-DRB1, FCGR1B, HLA-DQA1, and HLA-DRA. Among them, IRF5 (degree = 14), OAS3 (degree = 14), and HLA-A (degree = 14) had the highest connectivity with other proteins.

## 4. Discussion

Chronic nonbacterial osteomyelitis (CNO) is an autoinflammatory bone disorder. The more severe form of CNO is referred to as chronic recurrent multifocal osteomyelitis (CRMO). The molecular pathophysiology remains unknown, and no exact treatment protocol for this disease has been established. An understanding of the mechanisms underlying the pathophysiology of CNO/CRMO is vital for the development of effective novel therapeutics and diagnostic tools.

In the present study, the gene expression levels between CRMO and matched normal tissues were compared. A total of 1299 DEGs, including 1177 upregulated and 122 downregulated mRNAs, were identified to be associated with CRMO. DEGs were observed and analyzed using GO term and KEGG pathway analyses. The PPI network analysis was applied to identify key genes and significant modules associated with CRMO pathogenesis.

As shown in the GO analysis, in terms of biological processes, numerous genes were enriched in terms that were associated with immune function, such as GO:0019884 antigen processing and presentation of exogenous antigen, GO:0048002 antigen processing and presentation of peptide antigen, GO:0002475 antigen processing and presentation via MHC class Ib, GO:0019883 antigen processing and presentation of endogenous antigen, and GO:0002218 activation of innate immune response. The GO analysis of the DEGs in module 5 showed that genes were enriched in GO terms such as GO:0019884 antigen processing and presentation of exogenous antigen, GO:0048002 antigen processing and presentation of peptide antigen, GO:0002504 antigen processing and presentation of peptide or polysaccharide antigen via MHC class II, and GO:0002699 positive regulation of immune effector process, which were also intensely related with immune function. These GO terms that DEGs were enriched in were similar with the global GO analysis results. Autoinflammatory osteopathies, including CRMO, are suggested to be the result of a dysregulated innate immune system [9]. It is suggested that the genes in module 5 might be primarily involved in the disease progression of CRMO. The DEGs in module 5 included IRF5, IFI6, BST2, IFITM2, RSAD2, ISG15, STAT1, DDX58, IFIT1, OAS3, HLA-A, IFI30, HLA-A, IFI30, HLA-DRB1, FCGR1B, HLA-DQA1, and HLA-DRA. Among them, IRF5 (degree = 14), OAS3 (degree = 14), and HLA-A (degree = 14) owned the highest connectivity with other proteins. The interferon regulatory factor 5 (IRF5) gene, as a member of the interferon regulatory factor (IRF) family, participates in the type I interferon (IFN) signaling pathway and mediates induction of proinflammatory cytokines such as interleukin-6 (IL-6), IL-12, IL-23, and TNF-*α* [16, 17]. A number of studies had indicated that the IRF5 gene contributes to the pathogenesis of varied inflammatory and autoimmune diseases, such as rheumatoid arthritis, human lupus, systemic sclerosis, and inflammatory bowel disease [16, 18]. The IRF5 gene might be associated with the pathogenesis of CRMO, which is believed to be an autoinflammatory bone disorder. The 2′,5′-oligoadenylate synthetase (OAS) family is an IFN-induced enzyme that is involved in the activation of ribonuclease L (RNase L) [19]. It had been indicated that OAS3 is associated with the early inflammatory response to viruses or bacteria and may also play a role in cell growth, apoptosis, differentiation, or gene regulation [19–21]. HLA-A is one of three major types of the human major histocompatibility complex (MHC) class I cell surface receptors. It is known that HLA-A plays a vital role within the immune system through its involvement in adaptive and innate immune responses [22]. The exact role of these genes in CRMO pathogenesis calls for further exploration.

In terms of biological processes, the DEGs were also found to be enriched in “electron transport chain,” “translational initiation,” and “energy derivation by oxidation of organic compounds” in the GO analysis. In terms of cellular components, the DEGs were mainly enriched in “respiratory chain complex,” “respiratory chain,” “oxidoreductase complex,” “mitochondrial respiratory chain complex I,” “NADH dehydrogenase complex,” “mitochondrial respiratory chain,” and “ribosomal subunit.” In terms of molecular function, the DEGs were enriched in “oxidoreductase activity” and “acting on NAD(P)H.” In addition, based on KEGG pathway analysis, genes were enriched in pathways such as “oxidative phosphorylation” and “ribosome.” It is suggested genes or pathways relating to mitochondria and ribosomes might have important roles in the pathophysiology of CRMO. And in the PPI network analysis, 40 genes were found to own high connectivity with other genes in module 2. Among them, 31 encoded ribosomal proteins (RBs), while 9 other genes, including FAU, EIF3G, UPF1, MAGOH, EIF3F, EEF1B2, TPT1, SPCS3, and EEF1A1, were also associated with the function of ribosomes. It has indicated that DEGs in module 2 were intensely connected with each other. Accumulating evidences have shown that mitochondria are a hub of the immune system [23]. By regulating metabolic states as well as mitochondrial reactive oxygen species production, mitochondria are involved in many immune cell functions [24]. In stressed or damaged cells, mitochondrial components are released and are also a source of immunogenicity. Ribosomal proteins have also been shown to be involved in immune signaling and play diverse roles in host immune response [25]. For example, TPT1 can induce the release of cytokines and other signaling molecules from multiple types of immune cells [26]. Moreover, upregulation of EEF1A1 in Sahiwal cows significantly associates with immune function and inflammatory response [27]. Mitochondria and ribosomes are also involved in a variety of cellular phenomena, such as apoptosis, cell cycle, proliferation, and differentiation [25, 28]. The exact molecular mechanisms of mitochondria and ribosomes in CRMO still remain unknown and call for further exploration.

Certain limitations are associated with the present study. The sample size in our study was relatively small. In addition, only bioinformatics approaches were used in the study due to the rarity of this condition. Larger numbers of samples are also required for further investigation to validate the results obtained.

## 5. Conclusion

Oxidative phosphorylation, ribosome, and Parkinson disease pathways were significantly associated with CRMO. The immune-related genes including IRF5, OAS3, and HLA-A, as well as numerous ribosomal-related genes, might be implicated in the pathogenesis of CRMO. The identification of these genes may contribute to the development of early diagnostic tools, prognostic markers, or therapeutic targets in CRMO.

## Figures and Tables

**Table 1:** The top 10 DEGs between CRMO and matched normal tissues within GSE133378 data sets.

Gene symbol	Base mean	LogFC	lfcSE	Stat	*P* value	FDR
PSMB1	492.5637	3.23197	0.339503	9.519701	<0.000001	<0.000000001
CSTB	117.7362	4.06927	0.490468	8.296703	<0.000001	<0.000000001
FTL	1339.244	2.43132	0.324784	7.485968	<0.000001	<0.000000001
LAMP2	297.502	2.382048	0.339133	7.023935	<0.000001	0.000000007
RPS2P7	86.70384	3.342528	0.478375	6.987258	<0.000001	0.000000007
BCL9L	50.66015	-2.16449	0.442774	-4.88848	0.000001	0.000254625
LOC728535	88.75019	-1.68466	0.386537	-4.35833	0.000013	0.001401282
RBMX	249.9677	-1.36433	0.337952	-4.03706	0.000054	0.004287488
LOC613037	168.746	-1.61427	0.409896	-3.93825	0.000082	0.005758674
RRAS2	90.51448	-1.32056	0.381311	-3.46321	0.000534	0.024275822

## Data Availability

The microarray data used to support the findings of this study are from the GSE133378 data sets (https://www.ncbi.nlm.nih.gov/geo/query/acc.cgi?acc=GSE133378) and can be downloaded from the GEO (https://www.ncbi.nlm.nih.gov/geo) of NCBI. The processed data are available from the corresponding author upon request.

## Supplementary Materials

Supplementary 1 Supplementary Figure 1 The plot of PCA analysis between CRMO samples and control sample.

Supplementary 2 Supplementary Figure 2 The box plot of gene expression value before (a) and after (b) normalization and the density of raw data (c) and filtered data (d).

Supplementary 3 Supplementary Figure 3 The PPI network based on DEGs with degree > 6.

Supplementary 4 Supplementary Figure 4 The top 30 enriched GO terms of DEGs in modules 1–8 (a–h, respectively).

Supplementary 5 Supplementary Figure 5 The top 10 significant KEGG signaling pathways that DEGs in modules 1–8 (a–h, respectively) were enriched in.

Supplementary 6 Supplementary File 1 The details of all the DEGs.

Supplementary 7 Supplementary File 2 The detailed results of the GO enrichment analyses of all the DEGs.

Supplementary 8 Supplementary File 3 The detailed results of the KEGG pathway analyses of all the DEGs.
