# Supplementary material for: Genomic Expression Profiling and Bioinformatics Analysis of Chronic Recurrent Multifocal Osteomyelitis
Source: Biomed Res Int. 2021 Feb 9;2021:6898093. doi: 10.1155/2021/6898093 (PMC7888306; doi:10.1155/2021/6898093)
Supplement: Supplementary 6 — Supplementary File 1 The details of all the DEGs. [file 6898093.f6.pdf]

GeneID	Gene.syml	baseMean	logFC	lfcSE	stat	PValue	FDR
5689	PSMB1	492.5637	3.23197	0.339503	9.519701	1.74E-21	2.13E-17
1476	CSTB	117.7362	4.06927	0.490468	8.296703	1.07E-16	6.57E-13
2512	FTL	1339.244	2.43132	0.324784	7.485968	7.10E-14	2.91E-10
3920	LAMP2	297.502	2.382048	0.339133	7.023935	2.16E-12	6.62E-09
86781	RPS2P7	86.70384	3.342528	0.478375	6.987258	2.80E-12	6.88E-09
10581	IFITM2	2159.19	2.473191	0.361171	6.847693	7.51E-12	1.54E-08
6386	SDCBP	273.5472	2.462441	0.361259	6.816272	9.34E-12	1.64E-08
1915	EEF1A1	1493.622	2.60243	0.384334	6.771263	1.28E-11	1.96E-08
7114	TMSB4X	2581.911	3.920136	0.585325	6.697361	2.12E-11	2.90E-08
6426	SRSF1	97.43666	3.234613	0.484399	6.67758	2.43E-11	2.98E-08
6036	RNASE2	100.9149	3.252232	0.500644	6.496091	8.24E-11	9.20E-08
4170	MCL1	960.7553	2.420114	0.374885	6.455611	1.08E-10	1.10E-07
11345	GABARAPL1	110.5201	3.092886	0.482636	6.408314	1.47E-10	1.39E-07
23492	CBX7	160.1396	2.954944	0.463528	6.374895	1.83E-10	1.61E-07
55544	RBM38	452.1999	2.867801	0.459878	6.236	4.49E-10	3.67E-07
6891	TAP2	214.9342	2.526543	0.410245	6.158615	7.34E-10	5.63E-07
285045	LINC0048(	240.4686	2.181415	0.361745	6.030258	1.64E-09	1.18E-06
56666	PANX2	58.22881	3.502241	0.585056	5.986166	2.15E-09	1.47E-06
50854	C6orf48	163.5332	2.967623	0.50398	5.888374	3.90E-09	2.24E-06
401316	EIF4HP1	36.27378	4.763337	0.808418	5.89217	3.81E-09	2.24E-06
6277	S100A6	961.7563	2.485616	0.420883	5.90571	3.51E-09	2.24E-06
10106	CTDSP2	154.9161	3.029587	0.514943	5.883341	4.02E-09	2.24E-06
14	AAMP	80.68037	2.857903	0.488034	5.855953	4.74E-09	2.53E-06
59286	UBL5	177.1674	1.845272	0.316235	5.835137	5.37E-09	2.75E-06
10437	IFI30	259.4903	2.194243	0.377897	5.806454	6.38E-09	3.13E-06
10365	KLF2	105.8084	2.813724	0.488653	5.758122	8.51E-09	4.02E-06
6279	S100A8	1186.212	2.279448	0.396584	5.747708	9.05E-09	4.11E-06
6169	RPL38	847.5438	2.118785	0.377988	5.605424	2.08E-08	9.11E-06
6398	SECTM1	296.9423	3.479102	0.624725	5.56901	2.56E-08	1.08E-05
9553	MRPL33	31.50428	3.281456	0.593716	5.526981	3.26E-08	1.33E-05
29920	PYCR2	150.2676	3.53965	0.64175	5.515621	3.48E-08	1.38E-05
646195	RPS28P7	1188.966	2.746122	0.501442	5.476447	4.34E-08	1.61E-05
56655	POLE4	71.31313	2.594809	0.473668	5.47812	4.30E-08	1.61E-05
3122	HLA-DRA	935.6301	2.213844	0.406874	5.441106	5.30E-08	1.86E-05
4069	LYZ	934.5962	2.38983	0.438892	5.445143	5.18E-08	1.86E-05
684	BST2	244.1674	2.549867	0.476439	5.351928	8.70E-08	2.97E-05
1848	DUSP6	130.7128	2.51837	0.47237	5.33135	9.75E-08	3.23E-05
54940	OCIAD1	232.3372	1.888725	0.355554	5.312067	1.08E-07	3.41E-05
219988	PATL1	100.8544	2.399085	0.451455	5.314122	1.07E-07	3.41E-05
146225	CMTM2	229.6756	1.807295	0.350131	5.161774	2.45E-07	7.51E-05
730257	LOC73025'	72.67	2.613064	0.516624	5.057958	4.24E-07	0.000127
140717	RPL37AP1	19.26886	3.497307	0.693063	5.04616	4.51E-07	0.000132
6636	SNRPF	108.6667	2.166803	0.433294	5.000771	5.71E-07	0.000163
114769	CARD16	64.39771	2.192662	0.439093	4.993611	5.93E-07	0.000165
7280	TUBB2A	36.59507	3.596234	0.722831	4.975204	6.52E-07	0.000178
360	AQP3	21.78828	4.325612	0.882808	4.899832	9.59E-07	0.000252
54994	GID8	174.0617	2.039334	0.416313	4.898555	9.65E-07	0.000252
292	SLC25A5	45.44172	2.991946	0.611253	4.894773	9.84E-07	0.000252
283149	BCL9L	50.66015	-2.16449	0.442774	-4.88848	1.02E-06	0.000255
4616	GADD45B	59.37816	2.258229	0.463003	4.877356	1.08E-06	0.000264
3190	HNRNPK	748.3919	1.7507	0.360682	4.853859	1.21E-06	0.000282
729903	RPS16P10	15.3206	4.188373	0.863074	4.852854	1.22E-06	0.000282
9689	BZW1	64.14655	2.176353	0.447966	4.858298	1.18E-06	0.000282

11344	TWF2	331.026	2.679022	0.55451	4.831332	1.36E-06	0.000308
441951	ZFAS1	178.2944	1.599663	0.331794	4.821255	1.43E-06	0.000318
5997	RGS2	228.9873	2.75286	0.573369	4.801203	1.58E-06	0.000346
5660	PSAP	5610.105	1.673915	0.349416	4.790607	1.66E-06	0.000358
23480	SEC61G	18.42118	3.939326	0.824477	4.777967	1.77E-06	0.000363
4701	NDUFA7	41.2165	2.220493	0.464782	4.777491	1.77E-06	0.000363
51374	ATRAID	16.73102	3.961571	0.828775	4.780033	1.75E-06	0.000363
10957	PNRC1	95.19256	2.318599	0.486813	4.762817	1.91E-06	0.000379
1032	CDKN2D	314.6501	2.321181	0.487404	4.762332	1.91E-06	0.000379
1351	COX8A	101.8265	1.810727	0.381865	4.741799	2.12E-06	0.000413
29777	ABT1	28.46248	2.681973	0.566036	4.73817	2.16E-06	0.000414
3105	HLA-A	1151.411	1.792955	0.379688	4.72218	2.33E-06	0.000441
221838	EEF1A1P6	197.9793	1.953356	0.414604	4.711379	2.46E-06	0.000458
56882	CDC42SE1	72.31925	2.14317	0.455256	4.707614	2.51E-06	0.000459
1.05E+08	LOC10537(	141.6979	1.68183	0.357648	4.70247	2.57E-06	0.000464
1.02E+08	LOC10192!	303.0867	1.955511	0.416316	4.697177	2.64E-06	0.000469
2766	GMPR	172.4453	2.758569	0.590178	4.674131	2.95E-06	0.000499
25798	BRI3	221.3943	1.516784	0.324581	4.673047	2.97E-06	0.000499
4259	MGST3	77.54971	1.800799	0.385347	4.673183	2.97E-06	0.000499
55505	NOP10	39.83246	2.345007	0.501255	4.678268	2.89E-06	0.000499
115992	RNF166	74.66158	2.951859	0.632824	4.664583	3.09E-06	0.000513
5089	PBX2	573.9175	1.698655	0.365607	4.646119	3.38E-06	0.000546
10628	TXNIP	1097.743	1.783399	0.383664	4.648334	3.35E-06	0.000546
5292	PIM1	148.6191	2.535856	0.546482	4.640331	3.48E-06	0.000555
3310	HSPA6	36.99044	3.025188	0.653274	4.630808	3.64E-06	0.000571
83547	RILP	44.43085	2.544441	0.549655	4.629157	3.67E-06	0.000571
5216	PFN1	113.4626	2.471081	0.534715	4.621304	3.81E-06	0.000585
4696	NDUFA3	100.2802	1.917345	0.415471	4.614872	3.93E-06	0.000596
526	ATP6V1B2	329.2354	2.601238	0.564194	4.610539	4.02E-06	0.000601
6129	RPL7	84.24218	2.211109	0.48009	4.605616	4.11E-06	0.000601
374882	TMEM205	35.84483	2.139865	0.464572	4.606103	4.10E-06	0.000601
4318	MMP9	131.4771	2.711587	0.591648	4.583112	4.58E-06	0.000654
27013	CNPPD1	156.9419	1.775284	0.387286	4.583909	4.56E-06	0.000654
55577	NAGK	143.4832	1.848613	0.403743	4.578687	4.68E-06	0.00066
199	AIF1	254.8832	2.074335	0.453916	4.569869	4.88E-06	0.000681
1.03E+08	LOC10272(	780.3782	1.56961	0.343912	4.563987	5.02E-06	0.000692
51504	TRMT112	13.92283	4.413701	0.968712	4.556257	5.21E-06	0.00071
4792	NFKBIA	232.3831	1.843828	0.405562	4.546355	5.46E-06	0.000736
7127	TNFAIP2	204.4636	2.750212	0.606834	4.53207	5.84E-06	0.000779
8660	IRS2	243.3159	1.78638	0.394818	4.524564	6.05E-06	0.000799
10209	EIF1	769.5728	1.484154	0.330723	4.487606	7.20E-06	0.000941
1843	DUSP1	16.06484	3.370014	0.751485	4.48447	7.31E-06	0.000945
1675	CFD	253.1698	1.664319	0.371987	4.474136	7.67E-06	0.000981
81631	MAP1LC3B	74.59492	2.004284	0.448801	4.465861	7.97E-06	0.001009
6388	SDF2	34.52816	2.287037	0.512563	4.461963	8.12E-06	0.001017
1E+08	LOC10028'	59.5266	1.73659	0.389923	4.45367	8.44E-06	0.001047
113	ADCY7	130.9015	1.884964	0.42516	4.433543	9.27E-06	0.001138
7431	VIM	98.83678	1.730627	0.392137	4.413324	1.02E-05	0.001213
4707	NDUFB1	173.5507	1.567431	0.355033	4.414889	1.01E-05	0.001213
4535	ND1	2166.845	2.446345	0.553927	4.416366	1.00E-05	0.001213
22971	RPL41P1	200.9689	2.390568	0.54261	4.405685	1.05E-05	0.001239
150223	YDJC	31.01615	2.306425	0.523632	4.404669	1.06E-05	0.001239
6143	RPL19	414.5781	1.76298	0.401399	4.392086	1.12E-05	0.0013
1052	CEBPD	109.6727	1.92558	0.439402	4.382274	1.17E-05	0.001327

3726	JUNB	288.7024	2.102215	0.47977	4.381711	1.18E-05	0.001327
1050	CEBPA	60.03583	2.438119	0.556447	4.381584	1.18E-05	0.001327
746	TMEM258	220.615	1.713251	0.391429	4.376913	1.20E-05	0.001344
64207	IRF2BPL	432.1453	1.991017	0.455283	4.373144	1.22E-05	0.001355
6167	RPL37	1245.523	1.679647	0.385341	4.358857	1.31E-05	0.001401
728535	LOC72853!	88.75019	-1.68466	0.386537	-4.35833	1.31E-05	0.001401
6993	DYNLT1	75.46941	2.268607	0.519919	4.363385	1.28E-05	0.001401
28972	SPCS1	30.79581	2.365062	0.542693	4.358012	1.31E-05	0.001401
8666	EIF3G	33.11282	2.945726	0.678658	4.340515	1.42E-05	0.001505
339287	MSL1	142.4243	1.743148	0.402137	4.334713	1.46E-05	0.001532
415116	PIM3	288.2871	1.956034	0.452542	4.322322	1.54E-05	0.001607
567	B2M	3116.578	1.674562	0.387901	4.316987	1.58E-05	0.001632
101	ADAM8	355.9068	2.197494	0.509945	4.309278	1.64E-05	0.001676
4694	NDUFA1	251.6534	1.94292	0.451913	4.299318	1.71E-05	0.001738
30834	ZNRD1	31.2127	2.177551	0.508041	4.286169	1.82E-05	0.0018
11091	WDR5	64.47846	1.947784	0.454407	4.286432	1.82E-05	0.0018
808	CALM3	80.35284	1.901912	0.443737	4.286129	1.82E-05	0.0018
51123	ZNF706	27.92287	2.498177	0.588646	4.243938	2.20E-05	0.002157
81926	ABHD17A	114.0061	2.37731	0.561031	4.237395	2.26E-05	0.002203
4946	OAZ1	3220.257	1.780659	0.420623	4.233382	2.30E-05	0.002226
7305	TYROBP	537.4456	1.818607	0.430628	4.223155	2.41E-05	0.002311
9162	DGKI	180.6643	2.265442	0.537833	4.212169	2.53E-05	0.002407
51107	APH1A	757.4909	1.589667	0.377737	4.208393	2.57E-05	0.002411
8772	FADD	32.32382	2.185313	0.519238	4.208695	2.57E-05	0.002411
79132	DHX58	16.0012	3.18308	0.757532	4.201905	2.65E-05	0.002462
84317	CCDC115	41.55612	1.975773	0.470629	4.198154	2.69E-05	0.002484
64747	MFSD1	26.89945	2.257212	0.538813	4.189234	2.80E-05	0.002527
54472	TOLLIP	141.5103	2.203108	0.525756	4.19036	2.79E-05	0.002527
25801	GCA	88.15694	2.412287	0.57577	4.189673	2.79E-05	0.002527
219855	SLC37A2	40.56799	2.121431	0.506853	4.185492	2.85E-05	0.002532
517	ATP5G2	283.8351	1.513163	0.361462	4.186232	2.84E-05	0.002532
9230	RAB11B	142.9882	2.112611	0.50982	4.143839	3.42E-05	0.003017
10589	DRAP1	79.86348	2.485201	0.601173	4.133918	3.57E-05	0.003128
5552	SRGN	801.1274	1.811138	0.43849	4.130398	3.62E-05	0.003153
7846	TUBA1A	94.28399	1.901524	0.461266	4.122407	3.75E-05	0.003219
5432	POLR2C	47.68762	2.156672	0.52302	4.123495	3.73E-05	0.003219
50813	COPS7A	29.19298	2.702433	0.658615	4.103205	4.07E-05	0.003427
54935	DUSP23	30.45401	2.020919	0.492305	4.105016	4.04E-05	0.003427
6303	SAT1	221.6029	1.625861	0.39614	4.104262	4.06E-05	0.003427
10170	DHRS9	39.1785	2.384589	0.582276	4.095291	4.22E-05	0.003522
241	ALOX5AP	175.3691	1.533988	0.375196	4.088502	4.34E-05	0.003602
6957	TRB	1077.968	1.476834	0.362404	4.075101	4.60E-05	0.003784
10105	PPIF	17.46549	3.045227	0.747497	4.0739	4.62E-05	0.003784
64981	MRPL34	12.4026	3.411585	0.83987	4.062039	4.86E-05	0.003955
23399	CTDNEP1	252.8389	1.664826	0.41152	4.045551	5.22E-05	0.004217
2495	FTH1	1834.884	1.407453	0.348496	4.038649	5.38E-05	0.004286
11040	PIM2	200.6564	1.754541	0.434352	4.039447	5.36E-05	0.004286
27316	RBMX	249.9677	-1.36433	0.337952	-4.03706	5.41E-05	0.004287
3068	HDGF	179.1339	1.641134	0.407258	4.029718	5.58E-05	0.004367
7307	U2AF1	764.0513	1.888553	0.468565	4.030504	5.57E-05	0.004367
124460	SNX20	35.70888	2.270608	0.564344	4.023447	5.74E-05	0.004429
2537	IFI6	140.9047	1.574294	0.391261	4.023641	5.73E-05	0.004429
1200	TPP1	243.7706	1.191345	0.296273	4.02111	5.79E-05	0.004445
81622	UNC93B1	53.68494	2.219447	0.553423	4.010398	6.06E-05	0.004623

205251	LINC0011(	14.15713	2.928177	0.731906	4.000759	6.31E-05	0.004785
29796	UQCR10	50.71682	1.713753	0.428531	3.999133	6.36E-05	0.004789
140809	SRXN1	41.65143	2.383424	0.59681	3.993607	6.51E-05	0.004872
6836	SURF4	92.28314	1.742478	0.437026	3.987122	6.69E-05	0.004977
2050	EPHB4	23.84414	3.555798	0.893596	3.9792	6.91E-05	0.005114
6888	TALDO1	568.6581	1.541927	0.387946	3.974591	7.05E-05	0.005183
1E+08	RPL37AP5	11.75092	3.565544	0.899326	3.964685	7.35E-05	0.005371
1.05E+08	LOC10537(	15.86267	2.834172	0.716481	3.955685	7.63E-05	0.005544
92305	TMEM129	56.10977	2.421312	0.612347	3.954149	7.68E-05	0.005547
8477	GPR65	28.33946	2.350687	0.594734	3.952504	7.73E-05	0.005553
51714	SELT	63.66299	1.631623	0.413212	3.948639	7.86E-05	0.005611
8843	HCAR3	44.76758	2.450231	0.620812	3.94682	7.92E-05	0.005621
80256	FAM214B	23.11362	2.488947	0.631622	3.940565	8.13E-05	0.005736
613037	LOC61303'	168.746	-1.61427	0.409896	-3.93825	8.21E-05	0.005759
1337	COX6A1	72.04158	1.58235	0.402958	3.926835	8.61E-05	0.00597
23204	ARL6IP1	34.03299	1.885685	0.480108	3.927626	8.58E-05	0.00597
23479	ISCU	39.41452	2.009789	0.512401	3.922299	8.77E-05	0.00605
9636	ISG15	81.32314	2.095505	0.534938	3.917288	8.96E-05	0.006142
401152	C4orf3	55.15271	1.647594	0.421689	3.90713	9.34E-05	0.006336
9775	EIF4A3	25.26783	2.32066	0.593782	3.908272	9.30E-05	0.006336
6717	SRI	19.60495	2.384377	0.61103	3.902229	9.53E-05	0.00643
388	RHOB	19.98467	2.951566	0.756794	3.900094	9.62E-05	0.006451
4001	LMNB1	218.1674	1.278633	0.32829	3.894825	9.83E-05	0.006557
103910	MYL12B	132.4825	1.583863	0.4084	3.878217	0.000105	0.006984
10094	ARPC3	491.5799	1.70118	0.439731	3.868689	0.000109	0.007223
3663	IRF5	14.34463	2.817022	0.728961	3.864436	0.000111	0.007311
51327	AHSP	30.27183	2.702174	0.699502	3.862997	0.000112	0.007315
91689	SMDT1	32.10314	1.980171	0.513958	3.852783	0.000117	0.007587
51296	SLC15A3	45.65882	2.04474	0.534043	3.828791	0.000129	0.008322
80139	ZNF703	25.76439	2.743788	0.719818	3.811783	0.000138	0.008869
6207	RPS13	610.4137	1.79347	0.472416	3.796381	0.000147	0.009389
5692	PSMB4	39.18346	1.867636	0.493077	3.787715	0.000152	0.009524
23135	KDM6B	50.48684	2.708938	0.715185	3.787744	0.000152	0.009524
286444	RPS2P55	23.70756	2.191162	0.578442	3.788042	0.000152	0.009524
4537	ND3	1463.427	1.626926	0.429485	3.788082	0.000152	0.009524
5580	PRKCD	147.4388	1.857507	0.490636	3.785914	0.000153	0.009545
409	ARRB2	331.6486	1.850857	0.490125	3.776295	0.000159	0.009871
201163	FLCN	83.03791	1.502127	0.399212	3.762728	0.000168	0.01037
6168	RPL37A	8080.298	1.493079	0.397228	3.758749	0.000171	0.010483
3434	IFIT1	42.68572	2.380157	0.63456	3.750875	0.000176	0.010764
1340	COX6B1	144.3677	1.380555	0.368352	3.747927	0.000178	0.010784
7355	SLC35A2	15.42938	2.533358	0.675767	3.748862	0.000178	0.010784
220359	TIGD3	14.06199	2.75956	0.736567	3.746514	0.000179	0.010792
2876	GPX1	230.2863	1.563767	0.418671	3.735073	0.000188	0.01124
3191	HNRNPL	206.8327	1.179136	0.31637	3.727077	0.000194	0.011546
151579	BZW1P2	41.16945	2.961526	0.796227	3.71945	0.0002	0.011842
4814	NINJ1	72.00389	2.154994	0.580499	3.712313	0.000205	0.012065
6050	RNH1	94.61143	1.962981	0.528758	3.712441	0.000205	0.012065
2358	FPR2	19.50525	2.79326	0.753279	3.708134	0.000209	0.012207
23645	PPP1R15A	26.4673	2.12159	0.572771	3.704081	0.000212	0.012345
4097	MAFG	45.75951	1.968273	0.531739	3.701578	0.000214	0.012409
80149	ZC3H12A	28.91771	2.065672	0.558636	3.697709	0.000218	0.012541
1E+08	PET100	105.7331	1.417809	0.383994	3.692271	0.000222	0.012752
389342	RPL10P9	64.89453	5.136749	1.396267	3.678915	0.000234	0.013376

79887	PLBD1	143.8897	1.963142	0.535392	3.666736	0.000246	0.013964
3311	HSPA7	33.40458	2.952482	0.805982	3.663209	0.000249	0.014093
6817	SULT1A1	38.98155	1.710065	0.467907	3.65471	0.000257	0.014386
55625	ZDHHC7	158.3464	1.423509	0.389532	3.654404	0.000258	0.014386
4536	ND2	1503.534	2.057861	0.562761	3.656724	0.000255	0.014386
643246	MAP1LC3B2	22.68789	2.252199	0.616515	3.653111	0.000259	0.014394
55829	VIMP	41.28626	1.832865	0.502695	3.646077	0.000266	0.014727
516	ATP5G1	117.0346	1.424419	0.390803	3.64485	0.000268	0.014731
27243	CHMP2A	66.02549	1.781378	0.489162	3.641693	0.000271	0.014846
7097	TLR2	99.28674	1.311596	0.360301	3.640284	0.000272	0.014861
4758	NEU1	15.00352	2.67099	0.736057	3.62878	0.000285	0.015402
1347	COX7A2	122.6615	1.238185	0.341136	3.629599	0.000284	0.015402
7763	ZFAND5	50.04888	1.548588	0.427233	3.624689	0.000289	0.01558
60675	PROK2	128.3671	2.041818	0.563848	3.621223	0.000293	0.015721
89849	ATG16L2	616.8161	1.504854	0.416585	3.612354	0.000303	0.016198
220323	OAF	44.54894	1.520572	0.42264	3.597795	0.000321	0.017058
10801	9-Sep	1246.556	1.564746	0.435603	3.59214	0.000328	0.017285
3597	IL13RA1	83.84239	1.585786	0.441465	3.592098	0.000328	0.017285
3159	HMGA1	100.0755	1.63876	0.456662	3.588563	0.000333	0.017447
648217	RPL37P23	10.39011	4.037114	1.125803	3.585985	0.000336	0.017545
7454	WAS	131.0982	1.969617	0.550096	3.5805	0.000343	0.017841
4332	MNDA	211.6638	1.448995	0.404967	3.578056	0.000346	0.017933
2215	FCGR3B	1304.475	2.017621	0.565489	3.567922	0.00036	0.018563
2783	GNB2	138.6354	3.935637	1.104575	3.563031	0.000367	0.018656
2548	GAA	31.12274	2.612056	0.733207	3.562509	0.000367	0.018656
529	ATP6V1E1	134.0727	1.128832	0.316617	3.565288	0.000363	0.018656
4710	NDUFB4	69.46392	1.655581	0.464758	3.562241	0.000368	0.018656
6499	SKIV2L	39.00151	2.114264	0.594289	3.557634	0.000374	0.018908
28959	TMEM176B	37.27882	2.889472	0.812696	3.555417	0.000377	0.01899
1E+08	JHDM1D-AS	11.13139	2.88898	0.815963	3.540578	0.000399	0.020008
79930	DOK3	116.4237	1.665719	0.471087	3.535909	0.000406	0.020119
27240	SIT1	114.9667	1.775399	0.501979	3.536804	0.000405	0.020119
9741	LAPTM4A	286.1128	1.839849	0.520153	3.537131	0.000404	0.020119
6648	SOD2	362.536	1.502226	0.42513	3.533567	0.00041	0.020216
729737	LOC729737	21.59077	2.399798	0.679788	3.530214	0.000415	0.020392
201283	AMZ2P1	8.667238	3.350381	0.949784	3.527518	0.000419	0.020519
678	ZFP36L2	142.383	1.374595	0.390157	3.523182	0.000426	0.020775
5606	MAP2K3	579.2222	1.950583	0.554577	3.517243	0.000436	0.021162
4689	NCF4	96.69266	1.781191	0.507027	3.513009	0.000443	0.021417
5869	RAB5B	185.9741	1.699498	0.484923	3.504675	0.000457	0.022012
6776	STAT5A	127.2164	1.892103	0.540539	3.500399	0.000465	0.022281
27301	APEX2	13.33518	2.574933	0.737553	3.491181	0.000481	0.022974
9673	SLC25A44	92.86282	1.785081	0.511612	3.489132	0.000485	0.023061
1192	CLIC1	874.3765	1.870797	0.536566	3.48661	0.000489	0.02319
2114	ETS2	70.82688	1.49619	0.429524	3.483364	0.000495	0.023383
9919	SEC16A	59.37842	1.398868	0.403024	3.47093	0.000519	0.02394
54541	DDIT4	54.64529	1.623452	0.467468	3.47286	0.000515	0.02394
80198	MUS81	9.872492	3.446993	0.991892	3.475171	0.000511	0.02394
54344	DPM3	58.93453	1.584842	0.456509	3.471651	0.000517	0.02394
3428	IFI16	237.053	1.208406	0.348026	3.472168	0.000516	0.02394
642969	LOC642969	73.39534	1.316071	0.378737	3.474889	0.000511	0.02394
2219	FCN1	275.4528	1.758764	0.507499	3.465553	0.000529	0.024242
6391	SDHC	91.10145	1.396171	0.402754	3.466557	0.000527	0.024242
22800	RRAS2	90.51448	-1.32056	0.381311	-3.46321	0.000534	0.024276

79168	LILRA6	84.52999	2.125388	0.613711	3.463176	0.000534	0.024276
5880	RAC2	267.1637	1.484712	0.428984	3.460997	0.000538	0.024383
1992	SERPINB1	181.0101	1.436855	0.41539	3.459048	0.000542	0.02447
51734	MSRB1	104.8493	1.58613	0.458794	3.457169	0.000546	0.024551
1.01E+08	LINC0127:	50.55298	1.923903	0.557341	3.451929	0.000557	0.024941
11337	GABARAP	504.7989	1.29196	0.374409	3.450665	0.000559	0.024967
2029	ENSA	57.07585	1.808142	0.527677	3.426605	0.000611	0.027189
84074	QRICH2	13.03074	-2.54379	0.742594	-3.42554	0.000614	0.027197
338442	HCAR2	41.75516	1.975019	0.577731	3.418576	0.000629	0.027802
5696	PSMB8	67.48459	1.737392	0.509612	3.409245	0.000651	0.028668
7706	TRIM25	141.4111	1.447739	0.425517	3.40231	0.000668	0.0293
133283	EEF1A1P1:	24.33828	2.354956	0.694003	3.393292	0.000691	0.030067
116071	BATF2	17.67877	2.874738	0.846971	3.394139	0.000688	0.030067
84418	CYSTM1	59.76292	1.604633	0.474013	3.385206	0.000711	0.030641
643224	TUBBP5	13.75051	2.809546	0.829943	3.385227	0.000711	0.030641
57085	AGTRAP	37.84391	1.669067	0.493004	3.385507	0.00071	0.030641
90313	TP53I13	14.008	2.559895	0.756725	3.382862	0.000717	0.030796
25853	DCAF12	571.2653	1.917475	0.567779	3.377149	0.000732	0.031224
4722	NDUFS3	51.93495	1.424646	0.421829	3.377306	0.000732	0.031224
81628	TSC22D4	116.4111	2.124906	0.629936	3.373209	0.000743	0.03149
6039	RNASE6	44.21129	1.591086	0.471725	3.372913	0.000744	0.03149
6281	S100A10	87.04217	1.445069	0.428784	3.370154	0.000751	0.031698
689	BTF3	101.8616	1.381713	0.410472	3.366152	0.000762	0.031941
10423	CDIPT	35.62825	1.690997	0.502289	3.366584	0.000761	0.031941
6810	STX4	55.19143	1.505463	0.447978	3.360572	0.000778	0.032483
1E+08	SIGLEC14	29.92983	2.723516	0.811179	3.357477	0.000787	0.032737
9760	TOX	148.4711	-1.05105	0.31335	-3.35424	0.000796	0.033011
55330	BLOC1S4	24.46621	1.829093	0.545483	3.353164	0.000799	0.033028
3142	HLX	32.38363	2.114429	0.63081	3.351929	0.000803	0.033064
256586	LYSMD2	14.79429	2.472455	0.737987	3.350268	0.000807	0.033152
10899	JTB	107.8148	1.554036	0.464161	3.348051	0.000814	0.033262
4860	PNP	16.97155	2.079122	0.621096	3.347507	0.000815	0.033262
710	SERPING1	29.47108	2.415743	0.72218	3.34507	0.000823	0.033444
10491	CRTAP	48.77133	1.464888	0.439003	3.336849	0.000847	0.034335
22919	MAPRE1	45.59137	1.438034	0.431131	3.335488	0.000851	0.03439
11017	SNRNP27	64.91572	1.299615	0.389814	3.333939	0.000856	0.034459
1796	DOK1	33.16159	1.550442	0.465164	3.333107	0.000859	0.034459
26253	CLEC4E	32.35315	2.217604	0.666067	3.3294	0.00087	0.034808
387	RHOA	436.2339	1.159112	0.348245	3.328444	0.000873	0.034814
1084	CEACAM3	55.7478	1.627716	0.489737	3.323652	0.000888	0.035303
3927	LASP1	505.6496	1.258804	0.379656	3.315642	0.000914	0.036213
10636	RGS14	86.80337	1.838741	0.555759	3.308525	0.000938	0.036811
10866	HCP5	23.05526	1.890739	0.571514	3.3083	0.000939	0.036811
127829	ARL8A	337.5862	1.59023	0.480799	3.307474	0.000941	0.036811
1155	TBCB	90.63502	1.235286	0.373385	3.308347	0.000938	0.036811
2907	GRINA	438.7345	1.614866	0.488665	3.304645	0.000951	0.037032
3561	IL2RG	142.1953	1.588376	0.48074	3.304021	0.000953	0.037032
10928	RALBP1	142.4012	1.092386	0.331745	3.292842	0.000992	0.038414
51246	SHISA5	480.1868	1.33792	0.406451	3.29171	0.000996	0.038448
6929	TCF3	103.9659	1.451315	0.441026	3.290768	0.000999	0.038456
9537	TP53I11	8.834074	3.675616	1.11734	3.289613	0.001003	0.038494
80727	TTYH3	92.21716	1.80301	0.548868	3.284963	0.00102	0.038941
5859	QARS	33.49748	1.724757	0.525103	3.284607	0.001021	0.038941
51177	PLEKHO1	319.2472	1.295753	0.395621	3.275238	0.001056	0.040007

3689	ITGB2	4087.15	1.68224	0.513507	3.275985	0.001053	0.040007
440375	RAB43P1	21.83683	2.784849	0.850857	3.272994	0.001064	0.040202
552889	ATXN7L3B	32.39599	1.678466	0.513136	3.270993	0.001072	0.040269
54470	ARMCX6	31.35061	1.71554	0.524504	3.270788	0.001072	0.040269
5912	RAP2B	65.71838	1.442998	0.441345	3.269548	0.001077	0.040323
80213	TM2D3	42.1762	1.780076	0.544722	3.26786	0.001084	0.04044
2357	FPR1	224.4848	1.666715	0.5104	3.265509	0.001093	0.040654
402117	VWC2L	25.07478	1.703493	0.522676	3.259175	0.001117	0.041447
93621	MRFAP1	55.54058	1.455846	0.44705	3.25656	0.001128	0.041705
646531	YBX1P2	30.08017	2.797256	0.860809	3.249565	0.001156	0.042135
6881	TAF10	10.61985	2.616629	0.804856	3.251053	0.00115	0.042135
79719	AAGAB	82.48651	1.390135	0.42756	3.251322	0.001149	0.042135
79042	TSEN34	106.1098	1.743413	0.536534	3.249397	0.001156	0.042135
706	TSPO	153.5516	1.283971	0.394737	3.252728	0.001143	0.042135
4149	MAX	378.425	1.299578	0.400207	3.247263	0.001165	0.042327
6164	RPL34	513.2676	1.541572	0.475416	3.242577	0.001185	0.042526
5514	PPP1R10	121.2154	1.092265	0.336768	3.243374	0.001181	0.042526
647087	C7orf73	71.46815	1.398358	0.431137	3.243418	0.001181	0.042526
375	ARF1	300.0852	1.158252	0.356974	3.244641	0.001176	0.042526
10467	ZNHIT1	19.82471	1.883832	0.581195	3.241308	0.00119	0.042591
148932	MOB3C	75.60667	1.776067	0.550255	3.227717	0.001248	0.04428
10964	IFI44L	31.94317	2.033744	0.630015	3.228086	0.001246	0.04428
3930	LBR	82.96377	1.333277	0.413065	3.227763	0.001248	0.04428
219972	MPEG1	83.52419	1.31183	0.406675	3.225747	0.001256	0.04435
64748	LPPR2	33.17018	1.808217	0.560581	3.225613	0.001257	0.04435
3148	HMGB2	14.49978	2.602653	0.80716	3.224455	0.001262	0.04435
2987	GUK1	225.0842	1.168107	0.362411	3.223154	0.001268	0.04435
126792	B3GALT6	53.1774	1.382741	0.428986	3.223278	0.001267	0.04435
6521	SLC4A1	212.3406	1.383251	0.429293	3.22216	0.001272	0.044378
7392	USF2	243.086	1.458801	0.453124	3.21943	0.001284	0.044676
64231	MS4A6A	117.5977	1.072146	0.33329	3.216853	0.001296	0.044952
283922	LOC28392	50.75074	-1.51365	0.471572	-3.20981	0.001328	0.045939
6194	RPS6	744.4705	1.882005	0.586845	3.206986	0.001341	0.046261
79798	ARMC5	12.93536	2.679659	0.836664	3.202789	0.001361	0.046809
23558	WBP2	126.7631	1.487721	0.464715	3.201362	0.001368	0.04691
91782	CHMP7	19.9622	2.079215	0.649888	3.199343	0.001377	0.046977
1.05E+08	LOC10536	348.5826	-1.46079	0.456564	-3.19952	0.001377	0.046977
23526	HMHA1	202.5326	1.39626	0.43716	3.193935	0.001403	0.047734
5720	PSME1	121.8684	1.317612	0.413691	3.185015	0.001447	0.048878
10598	AHSA1	58.19537	1.236055	0.38822	3.183901	0.001453	0.048878
6923	TCEB2	145.4398	1.174326	0.368827	3.183952	0.001453	0.048878
55423	SIRPG	38.52948	1.544886	0.484991	3.185391	0.001446	0.048878
1627	DBN1	9.747954	2.948265	0.926401	3.182495	0.00146	0.048982
3150	HMGN1	63.28647	1.562269	0.491726	3.177111	0.001488	0.049764
10975	UQCR11	120.0692	1.256629	0.395689	3.175802	0.001494	0.049854
219285	SAMD9L	99.93881	1.306013	0.411462	3.174081	0.001503	0.049964
170622	COMMD6	49.76297	1.48562	0.46812	3.173586	0.001506	0.049964
23129	PLXND1	30.72545	1.713192	0.540776	3.168023	0.001535	0.050793
729614	FLJ37453	78.47701	1.46061	0.461684	3.163659	0.001558	0.051422
5329	PLAUR	201.7488	1.782067	0.5635	3.162495	0.001564	0.05149
6722	SRF	172.9905	1.441352	0.456075	3.160338	0.001576	0.051532
729683	LOC72968	11.31302	2.336704	0.739482	3.159921	0.001578	0.051532
6432	SRSF7	58.40907	1.68961	0.534452	3.161391	0.00157	0.051532
51728	POLR3K	23.23023	2.056434	0.651068	3.158555	0.001586	0.051637

50856	CLEC4A	35.22684	1.805938	0.572181	3.156234	0.001598	0.051912
283537	SLC46A3	44.1717	1.36312	0.432269	3.153405	0.001614	0.05228
6141	RPL18	1014.372	1.185327	0.37621	3.150707	0.001629	0.052626
6230	RPS25	105.693	1.493197	0.474261	3.148469	0.001641	0.052891
9445	ITM2B	297.8972	1.209726	0.384905	3.142923	0.001673	0.053483
79641	ROGDI	32.46007	2.012719	0.640247	3.143661	0.001668	0.053483
23218	NBEAL2	82.77281	1.30685	0.41563	3.144259	0.001665	0.053483
284207	METRNL	35.99474	1.532638	0.488293	3.138771	0.001697	0.054105
81876	RAB1B	48.63858	1.833678	0.584751	3.135825	0.001714	0.05451
53635	PTOV1	22.36353	2.040342	0.651483	3.131843	0.001737	0.055112
10285	SMNDC1	27.63052	1.623365	0.519184	3.126762	0.001767	0.055785
56729	RETN	31.27167	2.097375	0.670647	3.12739	0.001764	0.055785
79709	COLGALT1	211.0652	1.161889	0.371692	3.125946	0.001772	0.055797
811	CALR	123.4099	1.449289	0.463827	3.124632	0.00178	0.055903
1844	DUSP2	10.60183	2.791537	0.895282	3.118054	0.00182	0.057021
79145	CHCHD7	29.46862	1.707937	0.548071	3.116268	0.001832	0.057221
1350	COX7C	231.9943	1.195486	0.384051	3.112835	0.001853	0.057744
972	CD74	611.5516	1.208658	0.388583	3.110425	0.001868	0.05807
6452	SH3BP2	103.329	1.673937	0.53852	3.108402	0.001881	0.058174
23644	EDC4	26.22953	1.808254	0.581642	3.108878	0.001878	0.058174
90780	PYGO2	58.81902	1.325512	0.426659	3.106724	0.001892	0.058358
9535	GMFG	359.678	1.14798	0.370173	3.101198	0.001927	0.05931
57645	POGK	41.13007	1.410352	0.455054	3.099304	0.00194	0.059541
6280	S100A9	2483.592	1.198241	0.387166	3.094898	0.001969	0.060282
128346	C1orf162	33.22757	1.609662	0.520579	3.092061	0.001988	0.06071
83982	IFI27L2	35.82297	1.581471	0.51183	3.089836	0.002003	0.060723
4051	CYP4F3	9.967035	2.918418	0.94438	3.090301	0.002	0.060723
29801	ZDHHC8	12.88738	2.303891	0.745647	3.089789	0.002003	0.060723
23309	SIN3B	71.24625	1.543165	0.499737	3.087953	0.002015	0.060949
642546	HK2P1	7.575175	3.493807	1.131812	3.086915	0.002022	0.061011
928	CD9	32.37887	2.234486	0.724051	3.086091	0.002028	0.061021
441733	PRKXP1	13.60712	2.297425	0.744609	3.085412	0.002033	0.061021
3185	HNRNPF	119.2989	1.162961	0.377191	3.083217	0.002048	0.061174
1831	TSC22D3	673.6851	1.209568	0.392223	3.083879	0.002043	0.061174
1.05E+08	LOC10537[illegible]	27.85335	1.534106	0.498218	3.079186	0.002076	0.061707
2867	FFAR2	197.8228	1.967745	0.638899	3.079897	0.002071	0.061707
3003	GZMK	36.14815	1.560388	0.507376	3.075407	0.002102	0.062083
729402	RPL21P16	95.46862	1.475312	0.479553	3.076432	0.002095	0.062083
116540	MRPL53	55.31382	1.455159	0.473189	3.07522	0.002103	0.062083
11027	LILRA2	123.2825	1.526168	0.496482	3.073963	0.002112	0.062101
7841	MOGS	45.14715	1.561225	0.50793	3.073703	0.002114	0.062101
27230	SERP1	133.1767	1.081983	0.352335	3.070896	0.002134	0.062281
84750	FUT10	37.43725	-1.37226	0.446686	-3.07209	0.002126	0.062281
115207	KCTD12	26.02694	1.603017	0.522036	3.070702	0.002136	0.062281
283897	C16orf54	99.99406	1.835326	0.598996	3.064002	0.002184	0.063489
4513	COX2	1002.691	1.252764	0.408926	3.063544	0.002187	0.063489
6217	RPS16	729.9918	1.308378	0.427481	3.060672	0.002208	0.06395
1.02E+08	LOC10192[illegible]	128.7446	-1.10329	0.361201	-3.05451	0.002254	0.065124
55652	SLC48A1	30.35982	1.579321	0.517291	3.053062	0.002265	0.065187
58526	MID1IP1	26.86367	2.345467	0.768296	3.052815	0.002267	0.065187
51490	C9orf114	40.06232	-1.41383	0.463487	-3.05042	0.002285	0.065556
6737	TRIM21	15.05534	2.360555	0.774359	3.048398	0.002301	0.065691
6142	RPL18A	286.6691	1.306781	0.428652	3.048581	0.002299	0.065691
64110	MAGEF1	12.65144	2.678584	0.879919	3.044127	0.002334	0.066477

84826	SFT2D3	13.55869	2.289593	0.753099	3.040228	0.002364	0.067188
80774	LIMD2	157.7874	1.515746	0.498828	3.038615	0.002377	0.067392
6282	S100A11	100.4319	1.652098	0.544362	3.034923	0.002406	0.067859
54997	TESC	147.8429	1.278437	0.421499	3.033071	0.002421	0.067859
64386	MMP25	294.9982	1.707882	0.563042	3.033312	0.002419	0.067859
64859	NABP1	94.61755	1.474069	0.485813	3.034233	0.002411	0.067859
967	CD63	571.1914	1.210098	0.399157	3.031631	0.002432	0.068029
2210	FCGR1B	22.44636	2.093147	0.690862	3.029763	0.002447	0.068295
79180	EFHD2	195.0988	1.587876	0.525346	3.022533	0.002507	0.069789
3958	LGALS3	23.50949	1.892321	0.626971	3.018197	0.002543	0.070636
59274	MESDC1	20.58333	2.009584	0.667021	3.012773	0.002589	0.071586
1E+08	LOC10013:	27.32052	2.206523	0.732981	3.010342	0.00261	0.071678
7458	EIF4H	80.20303	1.262154	0.419205	3.010825	0.002605	0.071678
118472	ZNF511	9.993182	2.597071	0.86237	3.011551	0.002599	0.071678
10695	CNPY3	57.59473	1.414575	0.470668	3.005462	0.002652	0.072192
23586	DDX58	194.5946	1.220729	0.405997	3.006744	0.002641	0.072192
7316	UBC	584.6326	1.131565	0.376472	3.005711	0.00265	0.072192
6135	RPL11	488.0668	1.379026	0.458573	3.007213	0.002637	0.072192
54556	ING3	53.01333	1.242329	0.414024	3.000617	0.002694	0.073188
25924	MYRIP	29.16777	-1.50104	0.500527	-2.99892	0.002709	0.073435
5002	SLC22A18	20.77049	1.923143	0.642233	2.994464	0.002749	0.074162
56993	TOMM22	63.83333	1.286851	0.429712	2.994686	0.002747	0.074162
2771	GNAI2	663.2501	1.693752	0.565734	2.993902	0.002754	0.074162
2271	FH	11.14801	2.72625	0.911456	2.991093	0.00278	0.074684
51191	HERC5	35.38598	1.749578	0.585217	2.989625	0.002793	0.07488
79073	TMEM109	111.9879	1.683474	0.563368	2.98823	0.002806	0.075058
5382	PMS2P4	44.38388	-1.24795	0.418737	-2.98028	0.00288	0.076867
10018	BCL2L11	20.79352	1.677542	0.56453	2.971576	0.002963	0.078908
116138	KLHDC3	22.32613	1.86174	0.627689	2.966022	0.003017	0.079834
388588	SMIM1	23.94913	1.843261	0.621239	2.967071	0.003007	0.079834
23625	FAM89B	59.82668	1.292557	0.435792	2.965998	0.003017	0.079834
1396	CRIP1	275.3941	1.226342	0.413673	2.964521	0.003032	0.080046
6144	RPL21	297.4494	1.50964	0.509396	2.963588	0.003041	0.080117
25793	FBXO7	393.6772	1.337814	0.45153	2.962848	0.003048	0.080138
79872	CBLL1	27.01859	1.461925	0.493592	2.961807	0.003058	0.080237
646949	RPL23P6	30.88956	1.504453	0.509187	2.954618	0.003131	0.081781
7295	TXN	21.28138	2.218439	0.750722	2.955072	0.003126	0.081781
9467	SH3BP5	81.12565	-1.17311	0.39715	-2.95383	0.003139	0.081816
6155	RPL27	307.4878	1.610121	0.546054	2.94865	0.003192	0.083024
116151	FAM210B	548.2295	1.785893	0.606142	2.946326	0.003216	0.083473
271	AMPD2	50.63813	1.469674	0.499381	2.942992	0.003251	0.083926
23234	DNAJC9	12.97056	2.045306	0.695045	2.942694	0.003254	0.083926
3021	H3F3B	671.2344	1.17069	0.397756	2.943233	0.003248	0.083926
975	CD81	107.4505	1.279799	0.435386	2.93946	0.003288	0.084452
9592	IER2	816.794	1.421683	0.483645	2.939516	0.003287	0.084452
54458	PRR13	396.6852	1.158297	0.394407	2.936809	0.003316	0.085
515	ATP5F1	76.09416	1.186932	0.404295	2.935804	0.003327	0.085098
2214	FCGR3A	240.8527	1.484487	0.506533	2.930682	0.003382	0.086334
26277	TINF2	37.29398	1.824397	0.622921	2.928776	0.003403	0.086685
4874	NPM1P5	15.4831	2.763174	0.944144	2.926644	0.003426	0.0871
128646	SIRPD	62.41583	1.361319	0.466088	2.920737	0.003492	0.088585
53827	FXYD5	75.56905	1.323177	0.453162	2.919877	0.003502	0.088647
2994	GYPB	10.50218	2.261394	0.775847	2.914742	0.00356	0.089933
4673	NAP1L1	243.4017	1.2142	0.416779	2.913293	0.003576	0.090166

4610	MYCL	24.89698	1.839635	0.631643	2.912461	0.003586	0.090221
10945	KDELR1	203.263	1.583449	0.54421	2.909628	0.003619	0.090857
170954	PPP1R18	318.3451	1.024344	0.352307	2.907536	0.003643	0.09128
4061	LY6E	58.03834	2.006703	0.690521	2.906073	0.00366	0.091521
81873	ARPC5L	65.31714	1.131721	0.390362	2.89916	0.003742	0.092756
22821	RASA3	183.9334	1.13728	0.392341	2.898702	0.003747	0.092756
10289	EIF1B	20.51463	1.798735	0.620525	2.898732	0.003747	0.092756
5162	PDHB	20.96779	1.821423	0.627949	2.900592	0.003725	0.092756
79713	IGFLR1	72.97894	1.366963	0.471742	2.89769	0.003759	0.092869
5439	POLR2J	32.85192	1.659787	0.574109	2.891066	0.003839	0.093985
9588	PRDX6	79.78988	1.341705	0.463996	2.891629	0.003833	0.093985
602	BCL3	156.5688	1.886755	0.652473	2.891698	0.003832	0.093985
116092	DNTTIP1	77.45025	1.149199	0.397227	2.893054	0.003815	0.093985
1154	CISH	26.9621	1.638578	0.566826	2.890796	0.003843	0.093985
7106	TSPAN4	14.77236	2.159948	0.747531	2.889443	0.003859	0.094203
9825	SPATA2	7.439241	2.816237	0.975566	2.886773	0.003892	0.094629
10952	SEC61B	55.92331	1.103585	0.382761	2.883221	0.003936	0.094999
149986	LSM14B	31.20913	1.614144	0.559981	2.882498	0.003945	0.094999
80830	APOL6	253.0639	1.04634	0.363005	2.882443	0.003946	0.094999
4615	MYD88	33.75445	3.94947	1.369608	2.883651	0.003931	0.094999
3662	IRF4	17.39237	1.765636	0.613542	2.877774	0.004005	0.095295
7979	SHFM1	37.02868	1.499846	0.521063	2.878436	0.003997	0.095295
5250	SLC25A3	76.23674	1.231403	0.427779	2.878599	0.003994	0.095295
1E+08	NPIPB5	324.6729	-1.12458	0.390403	-2.88056	0.00397	0.095295
23207	PLEKHM2	261.0128	1.284537	0.446131	2.879285	0.003986	0.095295
339344	MYPOP	26.71284	1.478178	0.513604	2.878049	0.004001	0.095295
127687	C1orf122	16.24007	1.738693	0.605126	2.873273	0.004062	0.096105
10235	RASGRP2	597.92	1.346571	0.468577	2.873744	0.004056	0.096105
9121	SLC16A5	17.61439	1.712078	0.595848	2.873346	0.004061	0.096105
91543	RSAD2	32.30615	1.554884	0.541345	2.872262	0.004075	0.096228
1E+08	MTRNR2L9	11.58768	-2.99918	1.044435	-2.87158	0.004084	0.09625
83541	FAM110A	62.27274	1.447483	0.504725	2.867863	0.004133	0.097202
7873	MANF	22.0527	1.639293	0.57223	2.864746	0.004173	0.097976
8349	HIST2H2BI	25.53285	2.125135	0.743071	2.859936	0.004237	0.099161
348094	ANKDD1A	111.3364	1.431535	0.500585	2.859726	0.00424	0.099161
3134	HLA-F	99.38478	1.439839	0.503827	2.857804	0.004266	0.099447
1731	1-Sep	101.5877	1.154845	0.40413	2.857605	0.004269	0.099447
25983	NGDN	44.51521	1.28471	0.449882	2.855661	0.004295	0.099869
147040	KCTD11	71.08291	1.61879	0.567142	2.854293	0.004313	0.10011
6283	S100A12	69.50073	1.276842	0.44757	2.852831	0.004333	0.100382
3112	HLA-DOB	10.13508	2.277106	0.798566	2.851495	0.004351	0.100615
85365	ALG2	12.2148	2.052342	0.72009	2.85012	0.00437	0.100861
3045	HBD	22.51611	1.837875	0.64524	2.848357	0.004395	0.101108
65108	MARCKSL1	9.261605	2.523523	0.887148	2.844532	0.004448	0.102072
6660	SOX5	72.5116	-1.06959	0.376334	-2.84214	0.004481	0.102647
56911	MAP3K7CL	53.66179	1.266229	0.445612	2.841552	0.004489	0.102647
23401	FRAT2	126.5003	1.22457	0.431051	2.840891	0.004499	0.102669
3557	IL1RN	57.8089	1.404996	0.494724	2.839961	0.004512	0.102778
128977	C22orf39	49.11472	1.249715	0.440826	2.834942	0.004583	0.104213
3304	HSPA1B	8.632775	2.738189	0.966396	2.833402	0.004606	0.104523
10768	AHCYL1	113.7743	1.015983	0.358896	2.830856	0.004642	0.10497
8531	YBX3	213.2546	1.546868	0.546342	2.831316	0.004636	0.10497
51759	C9orf78	177.8267	1.370504	0.484955	2.826042	0.004713	0.106365
5095	PCCA	74.69637	-1.15774	0.409896	-2.82447	0.004736	0.106694

6513	SLC2A1	28.92406	1.461575	0.518814	2.817144	0.004845	0.108957
24138	IFIT5	11.27839	2.232698	0.792852	2.816033	0.004862	0.109134
9789	SPCS2	67.42359	1.240282	0.440796	2.813734	0.004897	0.109717
90864	SPSB3	65.82566	1.280547	0.455413	2.811835	0.004926	0.109966
84836	ABHD14B	57.41475	1.700104	0.604573	2.812076	0.004922	0.109966
6187	RPS2	790.8392	1.109835	0.395259	2.807865	0.004987	0.111129
119016	AGAP4	108.0061	-1.26723	0.451721	-2.80533	0.005026	0.111678
353	APRT	69.4926	1.312914	0.468044	2.805108	0.00503	0.111678
6478	SIAH2	96.57891	1.315837	0.469492	2.802683	0.005068	0.111914
132720	C4orf32	22.38505	1.508876	0.538261	2.803242	0.005059	0.111914
6500	SKP1	106.2516	1.073206	0.383071	2.801581	0.005085	0.112095
126003	TRAPPC5	113.4324	1.023344	0.365371	2.800835	0.005097	0.112153
2268	FGR	668.6999	1.262398	0.451839	2.79391	0.005207	0.114378
55312	RFK	11.22268	2.304712	0.82535	2.792407	0.005232	0.114549
1727	CYB5R3	111.3254	1.282168	0.459184	2.792274	0.005234	0.114549
83606	GUCD1	203.1346	1.271231	0.45598	2.787911	0.005305	0.11569
11313	LYPLA2	173.6569	1.778918	0.638735	2.785065	0.005352	0.116504
64319	FBRS	55.5902	1.768573	0.635286	2.7839	0.005371	0.116678
7263	TST	26.49044	1.40788	0.505807	2.783433	0.005379	0.116678
1.01E+08	SPDYE11	8.834804	-2.49335	0.898439	-2.77521	0.005517	0.119249
1.02E+08	LOC10192'	12.55947	1.946757	0.702468	2.771309	0.005583	0.120474
27069	GHITM	42.61752	1.291698	0.466455	2.769179	0.00562	0.121052
51690	LSM7	53.9692	1.262249	0.455977	2.768229	0.005636	0.121193
27113	BBC3	224.3808	1.419659	0.513048	2.767108	0.005656	0.121398
51094	ADIPOR1	65.8556	1.273262	0.46046	2.765197	0.005689	0.121898
9976	CLEC2B	31.97248	1.387271	0.501948	2.763774	0.005714	0.121946
1650	DDOST	42.19186	1.254759	0.453925	2.764243	0.005706	0.121946
10498	CARM1	38.5232	1.402327	0.50931	2.753387	0.005898	0.125508
10399	GNB2L1	370.4797	1.120969	0.407549	2.750511	0.00595	0.126396
29015	SLC43A3	13.10018	1.965229	0.715092	2.748218	0.005992	0.127064
1051	CEBPB	250.2158	1.427481	0.519533	2.747621	0.006003	0.127076
4716	NDUFB10	36.97729	1.308555	0.47658	2.745718	0.006038	0.127376
5199	CFP	145.9477	1.012726	0.36933	2.74206	0.006106	0.128582
53339	BTBD1	67.44371	-1.09889	0.401055	-2.73998	0.006144	0.128955
124446	TMEM219	82.01293	1.106803	0.403919	2.740161	0.006141	0.128955
5045	FURIN	205.6718	1.207483	0.440864	2.738902	0.006164	0.129159
643909	SPDYE9P	9.58112	-2.40901	0.882149	-2.73084	0.006317	0.132138
901	CCNG2	16.97848	1.680192	0.61646	2.725548	0.006419	0.133817
8462	KLF11	14.0065	1.83693	0.673829	2.726108	0.006409	0.133817
65095	KRI1	27.9889	-1.39662	0.51281	-2.72346	0.00646	0.134438
9349	RPL23	234.6538	1.330585	0.488864	2.721791	0.006493	0.13489
83855	KLF16	9.870594	2.520863	0.927017	2.719327	0.006541	0.135441
54732	TMED9	626.6886	1.022867	0.376393	2.717548	0.006577	0.135639
6837	MED22	24.42692	2.011253	0.739943	2.71812	0.006565	0.135639
2002	ELK1	23.93078	1.830613	0.67372	2.717173	0.006584	0.135639
23787	MTCH1	84.6979	1.091159	0.402067	2.713877	0.00665	0.136538
79803	HPS6	11.88548	2.120157	0.782033	2.711083	0.006706	0.137464
51561	IL23A	17.40836	1.739702	0.642464	2.707858	0.006772	0.138532
84266	ALKBH7	31.32	1.537702	0.56796	2.707411	0.006781	0.138532
79143	MBOAT7	475.3788	1.345026	0.496957	2.706522	0.006799	0.138672
10969	EBNA1BP2	9.984465	2.333194	0.862706	2.704507	0.006841	0.139054
4553	TRNA	45.52074	-1.60762	0.594379	-2.7047	0.006837	0.139054
22928	SEPHS2	22.75056	1.487048	0.550297	2.702263	0.006887	0.139534
6237	RRAS	17.76807	1.821799	0.674051	2.702762	0.006877	0.139534

6742	SSBP1	71.03765	1.171846	0.434063	2.699713	0.00694	0.140376
1.02E+08	LOC10192'	9.99449	-2.10608	0.780383	-2.69878	0.006959	0.14053
4508	ATP6	2050.541	1.570263	0.581955	2.698255	0.00697	0.14053
6622	SNCA	83.93977	1.475244	0.547015	2.696899	0.006999	0.140872
2286	FKBP2	101.4607	1.199186	0.445423	2.692242	0.007097	0.142155
65244	SPATS2	58.83621	-1.016	0.377377	-2.69226	0.007097	0.142155
10673	TNFSF13B	112.1487	1.469185	0.545971	2.690958	0.007125	0.142239
81618	ITM2C	8.343816	2.306028	0.856954	2.690959	0.007125	0.142239
1475	CSTA	99.79458	1.295685	0.481756	2.689502	0.007156	0.14263
2995	GYPC	3466.572	1.257735	0.467774	2.688769	0.007172	0.142711
2752	GLUL	92.53895	1.265965	0.47136	2.685771	0.007236	0.143533
8482	SEMA7A	10.07218	2.094593	0.779852	2.685885	0.007234	0.143533
91056	AP5B1	81.64757	1.489839	0.555244	2.683213	0.007292	0.144402
55585	UBE2Q1	36.48371	1.309152	0.488515	2.679861	0.007365	0.145387
2355	FOSL2	127.0199	1.093481	0.408133	2.679226	0.007379	0.145429
23640	HSPBP1	31.65399	1.362968	0.50884	2.678577	0.007394	0.145478
6286	S100P	117.2502	1.257239	0.46989	2.675601	0.00746	0.146074
56951	C5orf15	28.14053	1.290796	0.482379	2.675897	0.007453	0.146074
64407	RGS18	37.23154	1.284172	0.480158	2.674475	0.007485	0.146332
4084	MXD1	602.0686	1.076476	0.402817	2.672372	0.007532	0.146593
58527	ABRACL	128.1849	1.225847	0.459385	2.668453	0.00762	0.148038
1104	RCC1	41.81839	1.139443	0.427361	2.666231	0.007671	0.148785
23229	ARHGEF9	41.3622	1.126019	0.422532	2.664929	0.0077	0.149127
25948	KBTBD2	102.0362	1.002977	0.37664	2.662958	0.007746	0.149705
48	ACO1	84.29284	1.12521	0.422653	2.662255	0.007762	0.149705
388436	LOC38843(	95.45825	1.420156	0.533484	2.662038	0.007767	0.149705
81847	RNF146	30.84773	1.227438	0.461681	2.658629	0.007846	0.150755
729230	CCR2	53.36043	1.399087	0.526618	2.656742	0.00789	0.151365
10869	USP19	29.18547	1.634936	0.616251	2.653036	0.007977	0.152436
115286	SLC25A26	75.42457	-1.01389	0.382199	-2.65278	0.007983	0.152436
10008	KCNE3	95.1386	1.213928	0.457699	2.65224	0.007996	0.152445
6227	RPS21	1980.253	1.399089	0.527977	2.649904	0.008051	0.153265
51258	MRPL51	32.45345	1.376786	0.520168	2.646812	0.008125	0.154434
441168	FAM26F	14.11914	2.045732	0.773591	2.644462	0.008182	0.15527
1.05E+08	LOC10537:	23.54588	-1.7034	0.644329	-2.64367	0.008201	0.155392
352961	HCG26	18.42775	1.859072	0.70393	2.640991	0.008266	0.15629
11024	LILRA1	23.53981	1.42785	0.540713	2.640679	0.008274	0.15629
513	ATP5D	28.49832	1.336535	0.506355	2.63952	0.008302	0.156344
1984	EIF5A	50.98603	1.083967	0.410921	2.637898	0.008342	0.156853
646309	NAMPTP1	19.17793	1.653163	0.626888	2.637096	0.008362	0.156984
11333	PDAP1	79.52891	1.065963	0.404425	2.635753	0.008395	0.157253
83596	BCL2L12	30.2783	1.458722	0.553494	2.63548	0.008402	0.157253
55066	PDPR	58.75817	-1.13991	0.432613	-2.63494	0.008415	0.157262
7450	VWF	11.02988	-1.97378	0.749403	-2.6338	0.008444	0.157554
3276	PRMT1	194.6772	1.598003	0.607313	2.631266	0.008507	0.158251
4706	NDUFAB1	49.04014	1.274587	0.485192	2.626976	0.008615	0.159535
2010	EMD	230.0518	1.044401	0.397764	2.625676	0.008648	0.159904
957	ENTPD5	24.86526	-1.32244	0.503914	-2.62434	0.008682	0.160291
1642	DDB1	142.2388	1.131085	0.431206	2.62307	0.008714	0.160408
1.01E+08	SNHG19	41.67931	1.15369	0.439751	2.623508	0.008703	0.160408
2926	GRSF1	52.62465	1.051443	0.40102	2.621923	0.008744	0.160708
140807	KRT72	8.404788	2.803739	1.069602	2.621292	0.00876	0.160765
84335	AKT1S1	97.03161	1.526295	0.582554	2.620006	0.008793	0.161132
506	ATP5B	109.518	1.025739	0.39158	2.61949	0.008806	0.161135

91869	RFT1	45.77083	1.146657	0.438454	2.615229	0.008917	0.162675
8761	PABPC4	51.15161	1.148455	0.439366	2.61389	0.008952	0.16283
274	BIN1	55.06003	1.418282	0.542527	2.614213	0.008943	0.16283
221491	C6orf1	26.36834	1.638284	0.627042	2.612717	0.008983	0.162876
2969	GTF2I	267.3214	1.019399	0.390303	2.611814	0.009006	0.162876
6218	RPS17	738.155	1.312619	0.502663	2.611329	0.009019	0.162876
55911	APOBR	68.21606	1.542968	0.591115	2.610266	0.009047	0.162876
9454	HOMER3	38.85283	1.478921	0.566568	2.610314	0.009046	0.162876
4697	NDUFA4	27.2426	1.747819	0.670799	2.605577	0.009172	0.16464
8535	CBX4	104.0594	1.132732	0.434723	2.605643	0.00917	0.16464
1455	CSNK1G2	143.7377	1.050667	0.403667	2.602806	0.009246	0.165492
5976	UPF1	82.90714	1.273862	0.489412	2.602841	0.009245	0.165492
6156	RPL30	518.2227	1.312811	0.50537	2.597724	0.009384	0.167717
6628	SNRPB	57.6185	1.407096	0.541924	2.596484	0.009418	0.168079
113829	SLC35A4	17.12262	1.580983	0.609524	2.593799	0.009492	0.168468
8031	NCOA4	330.2579	1.031328	0.397983	2.591387	0.009559	0.168468
51019	CCDC53	75.64392	1.003485	0.387009	2.592923	0.009516	0.168468
55647	RAB20	44.05918	1.251698	0.482452	2.594453	0.009474	0.168468
80344	DCAF11	186.9012	1.498036	0.577595	2.593574	0.009498	0.168468
1.02E+08	LOC10192'	56.57225	1.273419	0.491277	2.592057	0.00954	0.168468
4538	ND4	1304.979	1.266144	0.488628	2.591221	0.009564	0.168468
84243	ZDHHC18	106.8767	1.15406	0.445272	2.591807	0.009547	0.168468
2352	FOLR3	13.40069	1.827989	0.706082	2.58892	0.009628	0.169354
24139	EML2	329.7604	1.027035	0.396824	2.588138	0.00965	0.169497
1.05E+08	LOC10537	7.593417	2.277796	0.881487	2.584038	0.009765	0.17128
11151	CORO1A	1032.703	1.094714	0.423906	2.582447	0.00981	0.171826
4296	MAP3K11	119.1441	1.611037	0.624455	2.57991	0.009883	0.172847
284821	RPL13AP7	44.59724	1.197819	0.464654	2.57787	0.009941	0.173377
129607	CMPK2	25.13967	1.478904	0.573659	2.578018	0.009937	0.173377
51510	CHMP5	39.32524	1.264928	0.49107	2.575861	0.009999	0.17355
728658	RPL13AP5	259.095	1.46813	0.569884	2.576191	0.00999	0.17355
9094	UNC119	62.52249	1.419511	0.551145	2.575566	0.010008	0.17355
7086	TKT	1878.888	1.233488	0.478652	2.577005	0.009966	0.17355
3005	H1F0	12.49692	1.997451	0.77577	2.574799	0.01003	0.17369
1207	CLNS1A	52.79752	1.111673	0.431886	2.573996	0.010053	0.173849
4695	NDUFA2	8.891166	2.411084	0.937239	2.572538	0.010096	0.174337
2975	GTF3C1	28.87535	1.26272	0.490989	2.571787	0.010118	0.17439
10916	MAGED2	26.6806	1.600339	0.622346	2.571461	0.010127	0.17439
3936	LCP1	675.2639	1.153431	0.448777	2.570167	0.010165	0.174797
6747	SSR3	63.12173	1.357031	0.528433	2.568028	0.010228	0.175634
129880	BBS5	20.1323	2.040326	0.794808	2.567069	0.010256	0.175874
1.02E+08	LOC10192'	94.51611	-1.21648	0.473976	-2.56655	0.010272	0.175893
441150	C6orf226	14.24532	1.70575	0.665069	2.564771	0.010324	0.176356
9149	DYRK1B	29.56904	1.763131	0.687469	2.56467	0.010327	0.176356
92558	CCDC64	66.48376	-1.01489	0.395913	-2.56342	0.010365	0.176745
11094	CACFD1	9.637979	2.264683	0.88415	2.561424	0.010424	0.177519
2634	GBP2	136.8417	1.117586	0.436413	2.560848	0.010442	0.177567
1.01E+08	HOTAIRM1	88.63085	1.055482	0.412646	2.557836	0.010533	0.178251
84957	RELT	27.87704	1.277062	0.499324	2.557583	0.01054	0.178251
145853	C15orf61	8.834914	2.278759	0.890624	2.558609	0.010509	0.178251
10237	SLC35B1	29.76932	1.245979	0.487177	2.557551	0.010541	0.178251
83442	SH3BGRL3	224.2754	1.091895	0.427003	2.557112	0.010555	0.178251
6305	SBF1	67.5432	1.301736	0.50921	2.556385	0.010577	0.178379
27071	DAPP1	72.56282	1.088488	0.426119	2.55442	0.010636	0.178502

64283	ARHGEF28	15.73929	-1.64959	0.645753	-2.55452	0.010633	0.178502
51399	TRAPPC4	54.10347	1.249515	0.488967	2.555421	0.010606	0.178502
5037	PEBP1	140.0905	1.238079	0.484716	2.554236	0.010642	0.178502
432	ASGR1	10.24783	1.938233	0.759249	2.55283	0.010685	0.17898
25797	QPCT	40.4495	1.38064	0.541955	2.547519	0.010849	0.18148
51312	SLC25A37	2382.421	1.121244	0.440333	2.546355	0.010885	0.181839
5164	PDK2	18.15746	1.90517	0.748638	2.54485	0.010932	0.182376
22809	ATF5	54.52013	1.552272	0.610432	2.542909	0.010993	0.183108
80273	GRPEL1	17.80936	1.436919	0.565312	2.541817	0.011028	0.183219
1.02E+08	LOC10192(	39.10786	1.523553	0.599612	2.540901	0.011057	0.183452
3108	HLA-DMA	15.2833	1.694128	0.667105	2.539523	0.0111	0.183928
57050	UTP3	22.13754	2.11879	0.835026	2.537394	0.011168	0.184057
51522	TMEM14C	7.96273	2.250973	0.887103	2.537444	0.011167	0.184057
29074	MRPL18	17.12453	1.521229	0.599372	2.538036	0.011148	0.184057
85441	HELZ2	188.9826	2.695328	1.062083	2.537774	0.011156	0.184057
11273	ATXN2L	110.2333	-1.03017	0.406897	-2.53176	0.011349	0.186537
116448	OLIG1	21.74826	1.837106	0.726519	2.528642	0.01145	0.187953
410	ARSA	39.25345	1.737265	0.687495	2.526947	0.011506	0.18861
23406	COTL1	602.507	1.016448	0.402552	2.52501	0.01157	0.189401
5573	PRKAR1A	154.9606	1.058222	0.419352	2.52347	0.01162	0.189979
23382	AHCYL2	106.4529	-1.03598	0.410945	-2.52096	0.011704	0.190952
5699	PSMB10	71.40798	1.309574	0.519678	2.519972	0.011736	0.190952
55181	SMG8	13.77335	1.909064	0.757624	2.519804	0.011742	0.190952
5900	RALGDS	203.7176	1.152309	0.457541	2.51848	0.011786	0.191164
10287	RGS19	97.43806	1.06679	0.423548	2.518697	0.011779	0.191164
6257	RXRB	23.93341	1.397656	0.555331	2.516796	0.011843	0.191633
1.05E+08	LOC10537(	21.67346	-1.52166	0.60469	-2.51643	0.011855	0.191633
57572	DOCK6	20.93461	-1.60763	0.639047	-2.51567	0.011881	0.191682
85379	KIAA1671	40.67618	-1.14414	0.455121	-2.51391	0.01194	0.192387
54436	SH3TC1	90.60749	1.002259	0.398933	2.512351	0.011993	0.192601
23193	GANAB	380.3952	1.244378	0.495208	2.512837	0.011976	0.192601
57707	TLDC1	42.69808	-1.06034	0.422199	-2.51148	0.012023	0.192601
7737	RNF113A	12.23687	2.019972	0.80422	2.511716	0.012015	0.192601
23020	SNRNP200	159.7815	1.480052	0.589377	2.511214	0.012032	0.192601
10758	TRAF3IP2	9.188866	2.436145	0.970603	2.509929	0.012076	0.192992
282974	STK32C	31.60636	1.312272	0.523001	2.509119	0.012103	0.192992
6843	VAMP1	10.19607	2.035187	0.811074	2.509251	0.012099	0.192992
4688	NCF2	448.8094	1.024297	0.408407	2.508029	0.012141	0.193337
1E+08	TOPORS-A(	20.76224	1.384983	0.55257	2.506439	0.012195	0.193958
93643	TJAP1	22.31421	1.319164	0.526654	2.504804	0.012252	0.194605
392256	RPS26P6	9.157598	2.439917	0.975435	2.501362	0.012372	0.195999
81532	MOB2	31.30302	1.308959	0.523263	2.501531	0.012366	0.195999
427	ASAH1	137.4074	1.025159	0.409956	2.500654	0.012396	0.196138
3156	HMGCR	27.63881	1.441481	0.576736	2.499379	0.012441	0.196592
6128	RPL6	683.3451	1.337485	0.536926	2.491006	0.012738	0.20077
56905	C15orf39	51.0565	1.403469	0.563685	2.489811	0.012781	0.200901
159	ADSS	45.89323	1.108147	0.44502	2.490105	0.012771	0.200901
22937	SCAP	131.5634	1.143549	0.459519	2.488576	0.012826	0.201114
60559	SPCS3	252.695	1.07324	0.431469	2.487407	0.012868	0.201197
578	BAK1	93.75363	1.404831	0.564999	2.48643	0.012903	0.201197
84153	RNASEH2C	34.47507	1.247219	0.501525	2.486852	0.012888	0.201197
29902	FAM216A	14.62989	1.822325	0.732986	2.486165	0.012913	0.201197
55920	RCC2	64.39967	1.10462	0.444152	2.487032	0.012881	0.201197
6618	SNAPC2	11.22495	1.974305	0.795091	2.483117	0.013024	0.202413

126364	LRRC25	36.21115	1.302241	0.524354	2.483515	0.013009	0.202413
81030	ZBP1	53.24228	1.341335	0.5407	2.480738	0.013111	0.203255
3240	HP	10.67498	1.935285	0.780662	2.47903	0.013174	0.203864
57136	APMAP	181.8277	1.243042	0.501475	2.47877	0.013184	0.203864
55573	CDV3	159.207	1.213854	0.49019	2.476295	0.013275	0.204852
5213	PFKM	111.152	1.128342	0.455684	2.476148	0.013281	0.204852
4794	NFKBIE	10.38649	1.771952	0.716079	2.474521	0.013342	0.205529
8991	SELENBP1	83.43723	1.453007	0.587741	2.47219	0.013429	0.206111
56935	SMCO4	27.03674	1.584978	0.641146	2.472103	0.013432	0.206111
353514	LILRA5	38.73433	1.243751	0.503192	2.471722	0.013446	0.206111
440345	NPIPB4	1027.946	-1.03807	0.420172	-2.47058	0.013489	0.206513
6232	RPS27	2524.557	1.193992	0.483833	2.467775	0.013596	0.207878
10421	CD2BP2	50.35764	1.284703	0.521066	2.465529	0.013681	0.208408
682	BSG	103.2203	1.622567	0.658067	2.465657	0.013676	0.208408
3399	ID3	27.90484	1.841201	0.748141	2.461035	0.013854	0.210775
1.05E+08	LOC10537	33.38746	-1.22657	0.498639	-2.45984	0.0139	0.211219
643872	HMGN2P6	9.89838	2.175281	0.885007	2.457925	0.013974	0.212084
1901	S1PR1	24.40516	1.769591	0.720937	2.454572	0.014105	0.213544
3094	HINT1	439.8053	1.18658	0.484098	2.451114	0.014241	0.214793
203245	NAIF1	12.42634	1.754552	0.715832	2.451065	0.014243	0.214793
3636	INPPL1	180.431	1.226425	0.500415	2.450816	0.014253	0.214793
391712	TRIM61	10.39322	1.879691	0.767205	2.450049	0.014284	0.21492
9296	ATP6V1F	88.3108	1.005948	0.41198	2.44174	0.014617	0.219393
80142	PTGES2	16.25218	1.616484	0.662503	2.439964	0.014689	0.220205
201176	ARHGAP27	98.17754	1.141716	0.468282	2.438095	0.014765	0.220539
4357	MPST	32.75544	1.267084	0.51967	2.438247	0.014759	0.220539
4245	MGAT1	186.2804	1.135093	0.465659	2.437607	0.014785	0.220569
6130	RPL7A	269.963	1.086447	0.446582	2.432805	0.014982	0.22277
10227	MFSD10	27.5258	1.196983	0.492257	2.431624	0.015031	0.223161
9513	FXR2	90.16226	1.445118	0.594582	2.430476	0.015079	0.223599
3728	JUP	33.11244	1.478398	0.60885	2.428183	0.015175	0.224476
4779	NFE2L1	37.64486	1.255858	0.517165	2.428351	0.015168	0.224476
54809	SAMD9	74.07416	1.073156	0.442206	2.426826	0.015232	0.225046
120425	AMICA1	624.3432	1.080855	0.445939	2.423771	0.01536	0.226675
8394	PIP5K1A	692.3669	1.113728	0.459773	2.422345	0.015421	0.226882
1.01E+08	FRY-AS1	12.64881	-1.66657	0.688178	-2.42171	0.015448	0.226882
2038	EPB42	20.96052	1.45669	0.601516	2.421697	0.015448	0.226882
148789	B3GALNT2	30.26551	-1.1397	0.470505	-2.42229	0.015423	0.226882
5252	PHF1	18.48488	1.928898	0.796895	2.420517	0.015498	0.227231
90025	UBE3D	43.64598	-1.00038	0.41363	-2.41854	0.015583	0.227231
3087	HHEX	10.46796	1.906278	0.788058	2.418956	0.015565	0.227231
966	CD59	34.07992	1.128947	0.466518	2.419942	0.015523	0.227231
10291	SF3A1	96.73552	1.253119	0.51799	2.419197	0.015555	0.227231
8694	DGAT1	17.24302	1.478263	0.612175	2.414772	0.015745	0.228573
400823	FAM177B	27.14456	1.173825	0.486009	2.415233	0.015725	0.228573
79575	ABHD8	11.31976	1.841472	0.762389	2.415397	0.015718	0.228573
8717	TRADD	30.69768	1.256416	0.520583	2.413477	0.015801	0.229002
79134	TMEM185B	10.57215	1.869944	0.774905	2.413126	0.015816	0.229002
5813	PURA	65.573	1.29964	0.53885	2.411879	0.015871	0.229272
64757	1-Mar	15.52942	1.469067	0.609107	2.411837	0.015872	0.229272
284323	ZNF780A	15.71333	1.488593	0.617801	2.409501	0.015974	0.230473
221458	KIF6	28.29442	-1.33769	0.555516	-2.40802	0.016039	0.231076
144406	WDR66	21.47184	-1.31052	0.544305	-2.40769	0.016054	0.231076
10608	MXD4	89.77278	1.353376	0.562314	2.4068	0.016093	0.23137

1861	TOR1A	12.23609	1.738316	0.722585	2.405689	0.016142	0.231623
26012	NSMF	49.77652	1.786311	0.742581	2.405545	0.016148	0.231623
1.05E+08	LOC10537	10.50508	1.936863	0.805471	2.404634	0.016189	0.23193
3015	H2AFZ	456.5586	1.050712	0.437594	2.401108	0.016345	0.232819
6228	RPS23	2212.89	1.269094	0.528373	2.401891	0.016311	0.232819
9587	MAD2L1BP	8.198694	2.006933	0.835739	2.401388	0.016333	0.232819
10023	FRAT1	26.36004	1.293944	0.538829	2.401401	0.016332	0.232819
200185	KRTCAP2	71.03428	1.028731	0.428295	2.401923	0.016309	0.232819
3858	KRT10	40.63179	1.350276	0.562743	2.399455	0.01642	0.233602
81892	SLIRP	45.6368	1.106755	0.461392	2.398731	0.016452	0.233794
573	BAG1	161.4949	1.123528	0.469061	2.395271	0.016608	0.23558
4953	ODC1	33.72734	1.311351	0.547515	2.395094	0.016616	0.23558
399687	MYO18A	72.75654	-1.03955	0.434225	-2.39403	0.016664	0.235994
644338	RPL37AP8	10.02268	1.836303	0.767738	2.391836	0.016764	0.236052
4209	MEF2D	116.2654	1.064142	0.444898	2.391878	0.016762	0.236052
1.02E+08	LOC10192	35.37779	-1.11611	0.466561	-2.3922	0.016748	0.236052
51646	YPEL5	49.83671	1.058357	0.443028	2.388917	0.016898	0.23722
83719	YPEL3	159.5638	1.08726	0.455451	2.387215	0.016977	0.237943
6124	RPL4	236.238	1.178006	0.494105	2.384121	0.01712	0.239134
64773	PCED1A	63.01341	1.157818	0.485633	2.384141	0.017119	0.239134
54978	SLC35F6	21.82303	1.292582	0.542016	2.384767	0.01709	0.239134
6029	RN7SL1	42.47761	-1.19837	0.502991	-2.38249	0.017196	0.239922
8870	IER3	208.2849	1.162241	0.488463	2.379384	0.017342	0.24168
1.01E+08	OTUD6B-AS	22.86654	1.389186	0.584054	2.378525	0.017382	0.241969
90007	MIDN	422.691	1.046306	0.440188	2.376952	0.017456	0.242729
212	ALAS2	475.0992	1.315745	0.55419	2.374177	0.017588	0.244008
4502	MT2A	12.99441	1.640462	0.692098	2.370274	0.017775	0.244939
3615	IMPDH2	15.89522	1.462411	0.616903	2.370568	0.017761	0.244939
90427	BMF	71.97063	1.230495	0.519759	2.367433	0.017912	0.246155
4731	NDUFV3	47.09458	1.096694	0.463295	2.367161	0.017925	0.246155
63923	TNN	14.84655	1.612685	0.683494	2.359473	0.018301	0.249367
5034	P4HB	111.3145	1.083628	0.459341	2.359093	0.01832	0.249367
29058	TMEM230	33.21367	1.081758	0.458333	2.360204	0.018265	0.249367
962	CD48	59.8751	1.157098	0.490786	2.357641	0.018391	0.24979
3052	HCCS	12.08371	1.757647	0.745415	2.357944	0.018376	0.24979
1186	CLCN7	47.17258	1.32799	0.563542	2.356507	0.018448	0.250079
51003	MED31	14.86061	1.625576	0.690415	2.354491	0.018548	0.250532
282694	NASPP1	7.904654	-2.5946	1.102913	-2.35249	0.018648	0.250642
54108	CHRAC1	8.389439	2.357108	1.001983	2.352442	0.018651	0.250642
120071	GYLTL1B	16.09067	1.690483	0.719336	2.350061	0.01877	0.251871
4635	MYL4	16.12872	1.669413	0.710504	2.349618	0.018793	0.251896
11051	NUDT21	26.22562	1.245267	0.530363	2.347952	0.018877	0.25275
4514	COX3	2955.591	1.0536	0.449274	2.345117	0.019021	0.254125
10063	COX17	23.45018	1.375951	0.586722	2.345152	0.019019	0.254125
9332	CD163	12.84089	1.776424	0.758638	2.341595	0.019202	0.255701
1974	EIF4A2	26.85257	1.26673	0.541131	2.340895	0.019238	0.255903
285148	IAH1	41.75586	1.195315	0.511421	2.337242	0.019427	0.257859
1059	CENPB	53.82004	1.234134	0.528439	2.335436	0.019521	0.258828
10362	HMG20B	24.49933	1.401662	0.601429	2.330552	0.019777	0.261662
834	CASP1	49.06579	1.266895	0.544049	2.328639	0.019878	0.261872
64651	CSRNP1	17.53277	1.383699	0.594184	2.328738	0.019873	0.261872
84967	LSM10	31.35213	1.263778	0.542671	2.32881	0.019869	0.261872
4354	MPP1	152.7791	1.083171	0.465273	2.328031	0.01991	0.262016
29035	C16orf72	90.4671	1.015979	0.436718	2.326398	0.019997	0.262877

55303	GIMAP4	190.9378	1.046607	0.45044	2.323519	0.020151	0.263949
653234	AGAP10P	18.93781	-1.35147	0.581913	-2.32247	0.020208	0.263949
1.01E+08	FAM157C	94.13684	1.144341	0.492687	2.322653	0.020198	0.263949
2879	GPX4	23.64266	1.435423	0.617838	2.323299	0.020163	0.263949
23275	POFUT2	12.24471	1.6352	0.703967	2.322836	0.020188	0.263949
4524	MTHFR	25.96255	-1.1981	0.516823	-2.31821	0.020438	0.265822
6201	RPS7	216.5092	1.175673	0.507469	2.316739	0.020518	0.266581
5704	PSMC4	27.38448	1.209101	0.522202	2.315389	0.020592	0.266974
29802	VPREB3	51.92066	1.036291	0.447531	2.315575	0.020581	0.266974
10308	ZNF267	25.06077	1.489035	0.643365	2.314447	0.020643	0.26736
8337	HIST2H2A/	56.69719	1.106186	0.478161	2.313416	0.0207	0.26781
392437	FTLP2	27.11247	1.837738	0.795639	2.309765	0.020901	0.269564
90231	KIAA2013	126.9693	1.024566	0.443708	2.309101	0.020938	0.269569
728392	LOC72839:	27.39906	1.421474	0.615632	2.308966	0.020945	0.269569
79629	OCEL1	15.18818	1.506055	0.653655	2.304052	0.02122	0.271674
81037	CLPTM1L	90.78975	1.015827	0.441149	2.302685	0.021297	0.272374
10567	RABAC1	30.12051	1.233113	0.536196	2.299744	0.021463	0.273928
4987	OPRL1	9.213196	1.8939	0.823464	2.299918	0.021453	0.273928
1.05E+08	LOC10536(	9.043446	2.077008	0.903637	2.298499	0.021533	0.274546
91561	RPS2P40	77.84863	1.456305	0.63381	2.297699	0.021579	0.274785
1.02E+08	LOC10193(	30.08507	-1.11352	0.484772	-2.29699	0.021619	0.274785
7099	TLR4	46.925	1.054398	0.459714	2.293593	0.021814	0.276582
8228	PNPLA4	7.621971	2.025467	0.883345	2.292951	0.021851	0.276582
25978	CHMP2B	22.63765	1.279461	0.557928	2.293236	0.021834	0.276582
113878	DTX2	140.4491	1.430037	0.624077	2.291443	0.021938	0.277397
6647	SOD1	57.66332	1.040125	0.454317	2.289426	0.022055	0.278015
54980	C2orf42	29.92477	-1.05257	0.459684	-2.28978	0.022034	0.278015
59283	CACNG8	7.584244	-2.01268	0.879676	-2.28798	0.022139	0.278788
3181	HNRNPA2B	364.1951	1.205575	0.52712	2.287096	0.02219	0.279151
140700	SAMD10	9.26886	1.940514	0.848632	2.286638	0.022217	0.279201
25915	NDUFAF3	48.14622	1.064179	0.465498	2.286105	0.022248	0.279307
1939	EIF2D	19.96229	1.283204	0.561761	2.284251	0.022357	0.279813
4570	TRNN	166.8026	-1.06719	0.468273	-2.279	0.022667	0.282834
1.01E+08	TVP23C-Cl	35.32429	-1.07233	0.470861	-2.27738	0.022764	0.28375
1.02E+08	LOC10192'	34.69406	1.097529	0.482163	2.27626	0.02283	0.283877
3492	IGH	626.3826	1.081738	0.47529	2.275954	0.022849	0.283877
55851	PSENEN	114.0448	1.124901	0.494448	2.275065	0.022902	0.284032
54431	DNAJC10	33.63949	1.115498	0.49049	2.274251	0.022951	0.28435
201965	RWDD4	16.06954	1.363718	0.599866	2.27337	0.023004	0.28472
25989	ULK3	28.06923	1.110219	0.488846	2.271102	0.023141	0.285838
5538	PPT1	200.1361	1.090779	0.480788	2.268733	0.023285	0.286103
80028	FBXL18	36.13956	-1.05993	0.467327	-2.26806	0.023325	0.286103
3043	HBB	6188.582	2.919943	1.286929	2.268923	0.023273	0.286103
27102	EIF2AK1	92.22513	1.019945	0.449975	2.266669	0.02341	0.28686
1.05E+08	LOC10537:	29.48704	-1.24594	0.549991	-2.26538	0.02349	0.287304
6809	STX3	128.099	1.030482	0.45518	2.263903	0.02358	0.288076
8677	STX10	27.54545	1.202073	0.531585	2.261301	0.023741	0.288088
374907	B3GNT8	9.008933	2.468727	1.091565	2.261639	0.02372	0.288088
54973	CPSF3L	7.281314	2.093759	0.925595	2.262067	0.023693	0.288088
1.05E+08	LOC10537	9.789738	-2.10164	0.929834	-2.26023	0.023807	0.288548
728489	DNLZ	53.96148	1.094944	0.484575	2.259598	0.023846	0.288742
51503	CWC15	37.87403	1.192511	0.52802	2.258457	0.023917	0.289316
8875	VNN2	1908.389	1.068798	0.473894	2.255352	0.024111	0.290803
1.02E+08	LOC10192(	12.98438	-1.5965	0.708233	-2.2542	0.024184	0.291389

10555	AGPAT2	47.93872	1.234422	0.548203	2.25176	0.024337	0.292669
8314	BAP1	12.82865	1.550916	0.688684	2.251999	0.024322	0.292669
226	ALDOA	78.78535	1.079504	0.479989	2.24902	0.024511	0.293895
1.05E+08	LOC10537(	26.53571	1.176039	0.523036	2.248486	0.024545	0.29392
92610	TIFA	10.35844	1.730122	0.769785	2.24754	0.024606	0.294165
89797	NAV2	20.23791	-1.31198	0.584098	-2.24616	0.024694	0.294574
7277	TUBA4A	47.26807	1.229764	0.547565	2.245879	0.024712	0.294574
9531	BAG3	10.56701	1.784972	0.795832	2.2429	0.024903	0.296568
57343	ZNF304	14.84831	1.502058	0.670338	2.240748	0.025042	0.297361
4055	LTBR	118.2511	1.313216	0.586342	2.239676	0.025112	0.297672
11140	CDC37	203.3087	1.108416	0.49494	2.239497	0.025124	0.297672
5566	PRKACA	298.357	1.031797	0.461479	2.235848	0.025362	0.299346
79650	USB1	32.48869	1.259666	0.563858	2.234014	0.025482	0.299397
5111	PCNA	14.68675	1.542089	0.690236	2.234146	0.025473	0.299397
246210	IKBKGP1	45.87171	1.193442	0.53417	2.234197	0.02547	0.299397
57393	TMEM27	7.365975	2.045896	0.915981	2.233557	0.025512	0.299464
1.03E+08	LOC10272:	11.48814	1.955034	0.875589	2.232822	0.025561	0.299746
55437	STRADB	79.17145	1.239477	0.555644	2.230703	0.025701	0.301102
84969	TOX2	60.50688	-1.17488	0.527054	-2.22915	0.025804	0.302023
29126	CD274	11.48268	1.722833	0.773068	2.228565	0.025843	0.302189
80179	MYO19	15.03892	-1.39462	0.626046	-2.22767	0.025903	0.302598
1.05E+08	LOC10537	9.830221	-2.10638	0.945934	-2.22677	0.025963	0.302726
744	MPPED2	11.21498	1.6383	0.735861	2.226371	0.025989	0.302749
643646	HSD17B1P	8.270296	2.271074	1.020466	2.225527	0.026046	0.303087
1.01E+08	INAFM2	7.293341	2.266704	1.019822	2.222646	0.02624	0.304797
55695	NSUN5	12.14264	1.500029	0.675373	2.221037	0.026348	0.305479
2677	GGCX	11.14984	1.65628	0.745852	2.220653	0.026374	0.305479
27000	DNAJC2	19.55246	-1.3025	0.587257	-2.21794	0.026559	0.306763
97	ACYP1	20.8332	-1.25557	0.566329	-2.21702	0.026621	0.307198
5743	PTGS2	8.194731	2.291067	1.034704	2.214226	0.026813	0.308157
7003	TEAD1	12.53474	-1.53331	0.693231	-2.21182	0.026979	0.309576
1.03E+08	LOC10272	9.46353	-1.99266	0.90238	-2.20822	0.027229	0.311101
10857	PGRMC1	8.771589	1.979676	0.896558	2.208083	0.027238	0.311101
9246	UBE2L6	81.16989	1.100089	0.498515	2.206731	0.027333	0.311311
2077	ERF	156.1129	1.0463	0.474093	2.206951	0.027317	0.311311
7675	ZNF121	21.2629	1.194294	0.541905	2.20388	0.027533	0.313297
1.05E+08	LOC10537:	20.66183	-1.18885	0.539582	-2.20328	0.027575	0.313486
80196	RNF34	23.75149	1.177749	0.534805	2.202203	0.027651	0.313901
9045	RPL14	228.4905	1.022182	0.464274	2.20168	0.027688	0.313901
1E+08	LOC10013:	20.24977	-1.51799	0.690753	-2.19759	0.027978	0.315244
6856	SYPL1	22.80696	1.250368	0.569188	2.196758	0.028038	0.315244
286059	LOC28605!	31.55833	1.428094	0.64982	2.197675	0.027972	0.315244
1E+08	LOC10041!	17.08984	1.352566	0.615668	2.196909	0.028027	0.315244
2664	GDI1	22.07728	1.362039	0.620014	2.196786	0.028036	0.315244
219348	PLAC9	27.25469	-1.11401	0.507526	-2.19499	0.028164	0.3158
83892	KCTD10	28.81996	1.206576	0.54965	2.195172	0.028151	0.3158
64332	NFKBIZ	30.64144	1.136841	0.517803	2.195509	0.028127	0.3158
11282	MGAT4B	106.4506	1.085519	0.494669	2.194435	0.028204	0.31594
1874	E2F4	16.63361	1.497507	0.682515	2.1941	0.028228	0.31594
9228	DLGAP2	12.13005	-1.83661	0.837813	-2.19214	0.028369	0.316856
4303	FOXO4	34.59867	1.238428	0.565005	2.191889	0.028388	0.316856
9875	URB1	16.28316	-1.31415	0.599981	-2.19032	0.028501	0.317256
7442	TRPV1	22.39137	-1.1951	0.546792	-2.18566	0.02884	0.320164
221914	GPC2	12.9989	1.729765	0.793234	2.18065	0.029209	0.323383

6138	RPL15	359.1801	1.040251	0.477172	2.180034	0.029255	0.323597
9429	ABCG2	13.04855	-1.47904	0.679177	-2.17769	0.029429	0.324402
401505	TOMM5	18.67568	1.310961	0.602012	2.177633	0.029433	0.324402
50862	RNF141	35.48767	1.236302	0.567773	2.177457	0.029446	0.324402
1E+08	OST4	19.12195	1.330212	0.611051	2.176924	0.029486	0.324402
554226	ANKRD30BI	15.32568	-1.39954	0.642853	-2.17707	0.029475	0.324402
653479	MRPL45P2	40.46278	-1.04526	0.480592	-2.17495	0.029634	0.325735
11068	CYB561D2	17.98057	1.33596	0.61446	2.174201	0.02969	0.326059
283871	PGP	17.90667	1.24749	0.574458	2.171596	0.029886	0.327334
9219	MTA2	49.97259	1.041663	0.480218	2.169146	0.030072	0.329072
54985	HCFC1R1	7.586667	2.192366	1.012368	2.165581	0.030343	0.331454
54998	AURKAIP1	69.30142	1.00536	0.46448	2.164487	0.030427	0.331779
392748	RPS27P17	150.5759	-1.29547	0.59908	-2.16243	0.030585	0.333209
10395	DLC1	20.50946	-1.53588	0.710372	-2.16207	0.030613	0.333209
4145	MATK	34.76013	1.408022	0.651652	2.160696	0.030719	0.333775
11258	DCTN3	10.90027	1.598964	0.740334	2.159787	0.030789	0.334243
29087	THYN1	34.56955	1.284687	0.594945	2.159336	0.030824	0.334327
23780	APOL2	13.23799	1.739009	0.805771	2.158191	0.030913	0.334995
85027	SMIM3	11.66362	1.55176	0.719751	2.155967	0.031086	0.335285
4240	MFGE8	23.28401	1.848585	0.857515	2.155746	0.031104	0.335285
79159	NOL12	21.82006	1.237474	0.574	2.15588	0.031093	0.335285
2017	CTTN	18.22172	1.907029	0.885114	2.154558	0.031196	0.335697
285442	LOC28544:	8.728907	1.930429	0.896957	2.152198	0.031382	0.336511
4519	CYTB	948.1076	1.183958	0.550043	2.152482	0.031359	0.336511
2765	GML	11.40539	1.974053	0.917506	2.151542	0.031433	0.336771
23277	CLUH	29.32381	1.031269	0.479763	2.14954	0.031592	0.337876
4043	LRPAP1	59.4767	1.075843	0.500806	2.148223	0.031696	0.338404
11270	NRM	12.38236	1.509072	0.702381	2.148508	0.031673	0.338404
284194	LGALS9B	32.91905	1.156915	0.538934	2.146671	0.03182	0.339132
3119	HLA-DQB1	91.46758	1.095466	0.510626	2.145341	0.031926	0.339672
11253	MAN1B1	24.132	1.275833	0.595604	2.142082	0.032187	0.341565
1.01E+08	BOLA3-AS	10.87996	1.632365	0.761989	2.142241	0.032174	0.341565
1E+08	FCGR1C	12.43096	1.80036	0.840634	2.141668	0.03222	0.341623
3614	IMPDH1	61.12873	1.175538	0.549302	2.140059	0.03235	0.342113
6150	MRPL23	26.44549	1.393018	0.651456	2.138314	0.032491	0.343311
51439	FAM8A1	25.33807	1.126633	0.527781	2.13466	0.032789	0.34502
93185	IGSF8	9.115775	1.756479	0.823125	2.133916	0.03285	0.34502
9903	KLHL21	40.16451	1.125699	0.528528	2.129875	0.033182	0.345316
728877	LOC72887'	20.2019	-1.23044	0.577687	-2.12995	0.033176	0.345316
142940	TRUB1	10.60897	1.54376	0.724372	2.13117	0.033075	0.345316
1E+08	LOC10013	23.95196	1.225358	0.574501	2.132909	0.032932	0.345316
2935	GSPT1	49.70204	-1.05798	0.496366	-2.13145	0.033052	0.345316
7832	BTG2	54.53147	1.315451	0.617695	2.129611	0.033204	0.345316
147807	ZNF524	18.14122	1.454208	0.682897	2.12947	0.033215	0.345316
4923	NTSR1	7.941022	1.993445	0.935828	2.13014	0.03316	0.345316
3579	CXCR2	362.8723	1.117242	0.524034	2.132003	0.033007	0.345316
7421	VDR	38.13262	1.167677	0.549014	2.126861	0.033432	0.346391
1198	CLK3	21.63561	1.445875	0.679732	2.127126	0.03341	0.346391
124359	CDYL2	32.61697	-1.09962	0.517402	-2.12528	0.033563	0.346841
6535	SLC6A8	95.47137	1.268594	0.597085	2.124645	0.033616	0.346841
55246	CCDC25	23.2499	1.101659	0.519042	2.122485	0.033797	0.348033
3820	KLRB1	46.42478	1.140851	0.537494	2.122536	0.033793	0.348033
4478	MSN	1383.018	1.073092	0.505671	2.122116	0.033828	0.348033
9618	TRAF4	9.379654	1.631348	0.769243	2.120719	0.033945	0.348772

78992	YIPF2	25.71586	1.284676	0.606469	2.11829	0.034151	0.350293
1E+08	DSTNP3	9.903343	2.438574	1.151502	2.117734	0.034198	0.350482
29105	CFAP20	9.118035	1.764426	0.834825	2.113527	0.034556	0.353692
1667	DEFA1	47.07637	1.251538	0.593208	2.109779	0.034877	0.355192
7038	TG	34.35047	1.00726	0.477458	2.109629	0.03489	0.355192
6730	SRP68	23.90347	1.251438	0.592876	2.110793	0.03479	0.355192
8476	CDC42BPA	19.65997	-1.46405	0.693847	-2.11005	0.034854	0.355192
57140	RNPEPL1	90.54771	1.131484	0.53629	2.109834	0.034873	0.355192
65083	NOL6	9.028611	1.678135	0.79576	2.108846	0.034958	0.355308
474344	GIMAP6	37.46233	1.139029	0.541583	2.103147	0.035453	0.358559
432369	ATP5EP2	30.12103	1.100855	0.523594	2.102496	0.03551	0.358839
8818	DPM2	16.22629	1.548973	0.737363	2.100693	0.035668	0.359255
1824	DSC2	23.81701	1.688417	0.803622	2.101009	0.03564	0.359255
387694	SH2D4B	8.517004	-1.82307	0.868047	-2.1002	0.035711	0.359394
115827	RAB3C	13.76691	-1.4711	0.701345	-2.09755	0.035945	0.359431
55698	RADIL	7.601687	-1.93771	0.923795	-2.09756	0.035944	0.359431
813	CALU	41.98605	-1.02729	0.48974	-2.09762	0.035939	0.359431
114836	SLAMF6	27.67799	1.051883	0.50106	2.099316	0.035789	0.359431
25827	FBXL2	9.793555	-1.58689	0.756491	-2.0977	0.035932	0.359431
8626	TP63	14.12742	-1.30018	0.62006	-2.09687	0.036005	0.359438
22871	NLGN1	21.5822	-1.15095	0.549168	-2.09581	0.036099	0.359758
1016	CDH18	25.34319	-1.05104	0.50161	-2.09534	0.03614	0.359881
57326	PBXIP1	223.9677	1.009631	0.482108	2.0942	0.036242	0.360135
56061	UBFD1	10.96003	1.594169	0.761997	2.092092	0.03643	0.361593
9278	ZBTB22	8.365597	1.666153	0.79763	2.08888	0.036719	0.363866
64897	C12orf43	12.20118	1.401821	0.67108	2.088904	0.036716	0.363866
57171	DOLPP1	8.078406	1.945653	0.932036	2.08753	0.03684	0.364778
118487	CHCHD1	20.81932	1.333028	0.63878	2.086836	0.036903	0.365105
728358	DEFA1B	46.76755	1.238105	0.593942	2.084556	0.03711	0.366559
9569	GTF2IRD1	24.07694	-1.27243	0.611751	-2.07998	0.037528	0.367612
80310	PDGFD	39.05935	-1.02542	0.493074	-2.07964	0.037558	0.367612
1.03E+08	LOC10272	12.55133	1.635089	0.786298	2.079478	0.037573	0.367612
388077	IGHV1OR1	12.55133	1.635089	0.786298	2.079478	0.037573	0.367612
7090	TLE3	205.0231	1.10363	0.530478	2.080442	0.037485	0.367612
780	DDR1	10.80639	1.796721	0.865334	2.076332	0.037863	0.369249
3006	HIST1H1C	25.62683	1.197875	0.578187	2.071778	0.038286	0.371603
4521	NUDT1	24.49278	1.314626	0.634521	2.07184	0.03828	0.371603
375593	TRIM73	16.50231	1.603064	0.773901	2.071406	0.038321	0.371631
389362	PSMG4	22.37149	1.156722	0.55959	2.067086	0.038726	0.37351
4651	MYO10	24.77388	-1.19873	0.580091	-2.06645	0.038786	0.373794
79874	RABEP2	19.23257	-1.13719	0.550639	-2.06522	0.038902	0.374328
23547	LILRA4	15.18306	1.468896	0.711179	2.065437	0.038882	0.374328
50848	F11R	57.89335	1.171507	0.567434	2.064569	0.038964	0.374544
80325	ABTB1	57.54533	1.420643	0.688388	2.063724	0.039044	0.374784
133015	PACRGL	24.27401	-1.24804	0.604837	-2.06343	0.039072	0.374784
55657	ZNF692	21.31159	1.535597	0.744338	2.063037	0.039109	0.374849
151613	TTC14	59.63703	1.00381	0.486768	2.062192	0.039189	0.375326
54855	FAM46C	41.42089	1.408114	0.683114	2.061316	0.039273	0.375832
168544	ZNF467	31.53939	1.348959	0.654905	2.059778	0.03942	0.376651
1.03E+08	LOC10272	10.06424	1.574364	0.764733	2.05871	0.039522	0.376674
4343	MOV10	27.93559	1.32422	0.643303	2.058471	0.039545	0.376674
3250	HPR	12.44509	1.360613	0.660893	2.05875	0.039518	0.376674
1.01E+08	SLFN12L	55.17047	1.116341	0.542167	2.059035	0.039491	0.376674
149345	SHISA4	23.01853	3.227125	1.569392	2.05629	0.039755	0.378098

57731	SPTBN4	12.52988	-1.50308	0.731081	-2.05596	0.039786	0.378098
8509	NDST2	23.18998	1.137125	0.553651	2.053868	0.039988	0.379427
2648	KAT2A	19.58784	1.163195	0.566439	2.053522	0.040022	0.379452
8804	CREG1	9.901337	1.589359	0.775024	2.050723	0.040294	0.381441
337875	HIST2H2B/	9.194263	1.572796	0.7671	2.050315	0.040334	0.381512
55365	TMEM176A	12.25065	2.10335	1.026732	2.048587	0.040502	0.382236
10153	CEBPZ	24.24331	1.045013	0.510553	2.046827	0.040675	0.382688
10221	TRIB1	43.36408	1.08389	0.529878	2.045545	0.040801	0.382829
1026	CDKN1A	25.75253	1.399559	0.685131	2.042761	0.041076	0.383522
252839	TMEM9	19.16627	1.396402	0.683769	2.042214	0.04113	0.383737
10965	ACOT2	12.44144	1.400033	0.685948	2.04102	0.041249	0.384348
4685	NCAM2	14.11167	-1.38648	0.679381	-2.04079	0.041272	0.384348
7319	UBE2A	11.39351	1.408118	0.690048	2.040609	0.04129	0.384348
51389	RWDD1	15.69934	1.426856	0.700127	2.037997	0.04155	0.385486
54442	KCTD5	36.02883	1.128192	0.55363	2.03781	0.041569	0.385486
93081	TEX30	8.738415	-1.78017	0.874136	-2.03649	0.041701	0.386126
80349	WDR61	30.16414	1.024878	0.503193	2.036751	0.041675	0.386126
26355	FAM162A	24.72923	1.097731	0.539559	2.034496	0.041902	0.38638
57143	ADCK1	29.43672	-1.00744	0.495097	-2.03483	0.041868	0.38638
54915	YTHDF1	55.02832	1.022785	0.50275	2.034379	0.041913	0.38638
159013	CXorf38	30.63137	1.046248	0.515595	2.029202	0.042438	0.389668
5269	SERPINB6	25.26179	1.107526	0.54685	2.025281	0.042838	0.391931
400027	LINC0093	9.526383	1.572366	0.776326	2.025393	0.042827	0.391931
1509	CTSD	1321.722	1.013258	0.50087	2.022996	0.043074	0.393495
7423	VEGFB	13.54171	1.31698	0.65159	2.021177	0.043261	0.394316
80326	WNT10A	11.22785	1.679482	0.831038	2.020944	0.043286	0.394316
755	C21orf2	38.23067	1.1082	0.54932	2.017403	0.043653	0.395231
83607	AMMECR1L	23.56568	1.058214	0.52463	2.017065	0.043689	0.395231
2257	FGF12	17.71691	-1.21895	0.604567	-2.01624	0.043775	0.395305
6427	SRSF2	51.6	1.0154	0.503716	2.015818	0.043819	0.395305
823	CAPN1	165.3621	1.055475	0.524525	2.012249	0.044194	0.398392
196463	PLBD2	18.18095	-1.21496	0.603948	-2.0117	0.044251	0.39862
84245	MRI1	12.50368	1.391448	0.691892	2.011076	0.044317	0.398921
55738	ARFGAP1	49.07523	1.24605	0.620493	2.008162	0.044626	0.400006
3312	HSPA8	184.9145	1.006471	0.501475	2.007021	0.044747	0.400444
134430	WDR36	21.77766	1.155613	0.576073	2.00602	0.044854	0.400557
7071	KLF10	9.049111	1.758031	0.876489	2.005766	0.044881	0.400557
728005	CTGLF9P	11.83597	-1.52105	0.758488	-2.00537	0.044923	0.400557
27018	NGFRAP1	32.2006	1.349617	0.673751	2.003139	0.045162	0.402105
83638	C11orf68	24.05126	1.454144	0.726399	2.001852	0.045301	0.402752
25977	NECAP1	20.71391	1.173573	0.586538	2.000849	0.045409	0.403421
10262	SF3B4	7.474836	1.780316	0.890615	1.998973	0.045611	0.404343
912	CD1D	9.046049	1.613908	0.807515	1.998611	0.04565	0.404399
7321	UBE2D1	34.73863	1.044873	0.523124	1.99737	0.045785	0.404714
55565	ZNF821	26.06035	-1.0156	0.50845	-1.99744	0.045777	0.404714
199675	MCEMP1	35.76469	1.181243	0.591539	1.996897	0.045836	0.404877
65005	MRPL9	8.387947	1.564992	0.784099	1.995912	0.045943	0.40524
1.05E+08	SPDYE15P	11.51746	-1.54894	0.777619	-1.9919	0.046383	0.407921
57456	KIAA1143	45.48196	1.130539	0.567956	1.990541	0.046531	0.407921
641776	SPDYE14P	10.96225	-1.6629	0.836117	-1.98883	0.04672	0.408856
282991	BLOC1S2	60.3352	1.108191	0.557139	1.989072	0.046693	0.408856
1856	DVL2	35.55448	1.605806	0.807891	1.987651	0.04685	0.409124
10158	PDZK1IP1	34.81652	1.319414	0.664104	1.986757	0.046949	0.409697
7803	PTP4A1	61.41565	1.13161	0.569853	1.985793	0.047056	0.410048

23165	NUP205	36.58116	-1.0207	0.514265	-1.98478	0.047169	0.41045
10807	SDCCAG3	21.24532	1.284212	0.647953	1.981952	0.047485	0.412026
123879	DCUN1D3	17.64757	-1.27036	0.640887	-1.9822	0.047457	0.412026
3099	HK2	36.95934	1.070024	0.539882	1.981959	0.047484	0.412026
1.05E+08	LOC10537:	51.43839	-1.05568	0.533349	-1.97934	0.047778	0.413466
389792	IER5L	8.080349	1.772085	0.896637	1.976369	0.048113	0.414258
1872	E2F3P1	20.36668	1.145613	0.579609	1.976525	0.048095	0.414258
285141	ERICH2	16.45216	1.360914	0.689634	1.973387	0.048451	0.416333
8407	TAGLN2	1509.805	1.061777	0.538632	1.971248	0.048696	0.417517
3116	HLA-DPB2	9.128563	-1.68066	0.852936	-1.97045	0.048787	0.417805
7570	ZNF22	14.20093	1.361037	0.690982	1.969715	0.048871	0.417805
10452	TOMM40	11.07254	1.391967	0.706642	1.969833	0.048858	0.417805
27166	PRELID1	21.97793	1.349208	0.685646	1.967791	0.049092	0.418288
284837	AATBC	23.69261	1.025767	0.52222	1.964241	0.049502	0.419742
121273	C12orf54	8.70735	1.897582	0.96814	1.960029	0.049992	0.42317
1E+08	RPS27P9	3.688232	3.443047	1.452734	2.370047	0.017786	NA
5352	PLOD2	6.135013	-1.83797	0.919524	-1.99883	0.045627	NA
3280	HES1	3.419204	5.265002	1.630836	3.228406	0.001245	NA
133060	OTOP1	3.811009	-3.2498	1.448957	-2.24285	0.024906	NA
27065	NSG1	5.952033	2.224988	1.072055	2.075442	0.037946	NA
53409	ATP5LP3	6.154262	-4.10018	1.481826	-2.76698	0.005658	NA
2565	GABRG1	3.343643	3.304762	1.529417	2.160798	0.030711	NA
1.05E+08	LOC10537'	2.605432	-3.75178	1.677226	-2.23689	0.025293	NA
59350	RXFP1	6.568593	-2.12423	0.95001	-2.23601	0.025351	NA
1E+08	RPL21P51	3.995473	3.52549	1.39193	2.532807	0.011315	NA
170690	ADAMTS16	7.098816	-2.55609	1.075343	-2.377	0.017454	NA
10884	MRPS30	6.963175	2.105064	0.928254	2.267768	0.023343	NA
1.05E+08	LOC10537!	1.444238	-3.93884	1.999913	-1.96951	0.048895	NA
492311	IGIP	2.305081	4.699074	1.783844	2.63424	0.008433	NA
1.01E+08	CICP15	4.517652	2.559631	1.194099	2.143567	0.032068	NA
222698	NKAPL	5.500226	2.006975	1.017769	1.971935	0.048617	NA
1.01E+08	FPGT-TNN	4.767675	2.156475	1.089473	1.979374	0.047774	NA
1E+08	LOC10010	1.484721	-3.97405	1.990445	-1.99656	0.045873	NA
1.02E+08	LOC10192!	3.169239	4.148141	1.607094	2.581144	0.009847	NA
57212	TP73-AS1	6.468768	2.338343	1.05211	2.222527	0.026248	NA
347734	SLC35B2	3.464413	4.257352	1.585421	2.685314	0.007246	NA
7272	TTK	1.604363	-4.11211	2.002733	-2.05325	0.040048	NA
339448	C1orf174	6.641204	1.97522	0.999232	1.976737	0.048071	NA
68	ACTBP8	2.400316	-4.67252	1.740497	-2.68459	0.007262	NA
642741	RPL3P7	3.245828	3.313781	1.628328	2.035082	0.041843	NA
148418	SAMD13	2.296493	-3.5346	1.720695	-2.05417	0.039959	NA
6610	SMPD2	5.869264	2.2197	0.998898	2.22215	0.026273	NA
644303	LOC64430:	1.442217	4.047342	2.050851	1.973494	0.048439	NA
285762	LOC28576:	2.946634	2.988253	1.511342	1.977218	0.048017	NA
10370	CITED2	3.935143	4.452905	1.561712	2.851296	0.004354	NA
84624	FNDC1	6.801475	-1.85913	0.941346	-1.97497	0.048272	NA
644794	LOC64479	1.659185	4.24023	2.154471	1.968108	0.049056	NA
8468	FKBP6	2.891999	-2.97923	1.517327	-1.96347	0.049591	NA
54873	PALMD	3.525337	-4.19155	1.626077	-2.57771	0.009946	NA
391059	FRRS1	5.898685	-2.65392	1.192314	-2.22586	0.026024	NA
401397	LINC0099	2.155433	3.551185	1.737659	2.043661	0.040987	NA
10157	AASS	6.95247	2.228845	0.940637	2.369505	0.017812	NA
1.01E+08	LOC10102'	1.85363	4.396945	1.85688	2.367921	0.017888	NA
29803	REPIN1	6.135259	3.044606	1.09115	2.790273	0.005266	NA

349136	WDR86	4.497223	2.613208	1.136825	2.29869	0.021523	NA
1.01E+08	PRKAG2-A:	3.952499	2.915248	1.332405	2.187959	0.028673	NA
257	ALX3	2.564344	3.809884	1.680778	2.266738	0.023406	NA
51435	SCARA3	6.060527	-2.03439	0.962266	-2.11416	0.034502	NA
203100	HTRA4	4.919039	-2.26817	1.093351	-2.07451	0.038032	NA
84933	C8orf76	4.821935	2.457537	1.131375	2.172168	0.029843	NA
1.05E+08	LOC10537!	4.705805	2.399022	1.12178	2.138585	0.032469	NA
4609	MYC	1.596624	4.194621	2.003286	2.093871	0.036272	NA
113655	MFSD3	2.568853	4.863517	1.703756	2.854586	0.004309	NA
7002	PRDX1P1	5.745832	-2.17965	1.072357	-2.03258	0.042095	NA
441391	RBMXP2	7.232783	3.825842	1.471016	2.600815	0.0093	NA
1.05E+08	LOC10537(	6.339274	-2.41234	1.165991	-2.06892	0.038554	NA
1.05E+08	LOC10537{	5.196216	-3.22347	1.226215	-2.6288	0.008569	NA
2189	FANCG	4.018383	3.664185	1.447907	2.530678	0.011384	NA
403323	LOC40332:	5.608241	4.152611	1.339255	3.100686	0.001931	NA
1E+08	LOC10013:	2.612687	3.874416	1.834249	2.112263	0.034664	NA
1E+08	UNQ6494	2.60892	-4.78838	1.711653	-2.79752	0.00515	NA
9858	PPP1R26	1.604132	4.200996	1.969263	2.133283	0.032901	NA
1.02E+08	LOC10192{	2.587209	-3.7452	1.66366	-2.25118	0.024374	NA
645203	TMEM14D	5.483317	5.964538	1.529785	3.898939	9.66E-05	NA
118932	ANKRD22	3.609917	3.428765	1.544099	2.22056	0.026381	NA
199953	TMEM201	3.086768	3.051512	1.485416	2.054315	0.039945	NA
79751	SLC25A22	6.014161	3.195418	1.195212	2.673517	0.007506	NA
56675	NRIP3	3.957766	-3.44766	1.457867	-2.36487	0.018037	NA
7119	TMSB4XP5	2.306727	3.602847	1.723736	2.090138	0.036605	NA
56834	GPR137	6.091102	2.76252	1.152935	2.396076	0.016572	NA
728975	LOC72897!	3.964516	3.560867	1.450468	2.454978	0.014089	NA
84285	EIF1AD	7.213134	2.322372	0.971941	2.389415	0.016875	NA
8722	CTSF	6.976147	2.649188	0.938334	2.82329	0.004753	NA
9828	ARHGEF17	2.236776	4.679837	1.875956	2.494641	0.012608	NA
684959	SNORA25	1.781801	4.328903	2.085177	2.076037	0.037891	NA
729494	ARPC3P3	3.172208	5.160973	1.634701	3.157135	0.001593	NA
1E+08	ATF4P4	3.772181	-2.77463	1.368713	-2.02718	0.042644	NA
283174	MIR4697H(	6.70052	2.81807	1.054018	2.673646	0.007503	NA
9052	GPRC5A	2.934551	-3.91385	1.625127	-2.40834	0.016025	NA
114785	MBD6	3.647727	2.834977	1.380616	2.053414	0.040032	NA
196403	DTX3	6.635998	6.244889	1.481159	4.216219	2.48E-05	NA
1.05E+08	LOC10537	2.324732	-3.52937	1.719494	-2.05256	0.040115	NA
1.05E+08	LOC10536!	1.673084	-4.14169	1.916713	-2.16083	0.030708	NA
84747	UNC119B	6.164576	2.880892	1.071224	2.689347	0.007159	NA
28984	RGCC	5.547161	3.314284	1.205422	2.749481	0.005969	NA
6846	XCL2	2.885943	3.093781	1.535771	2.01448	0.043959	NA
4247	MGAT2	3.41087	2.611423	1.292447	2.020526	0.043329	NA
9240	PNMA1	2.476264	4.820084	1.721882	2.799312	0.005121	NA
283596	SNHG10	2.502028	4.815278	1.740839	2.766068	0.005674	NA
64423	INF2	6.347358	2.134799	1.062097	2.009985	0.044433	NA
122618	PLD4	1.606634	4.202741	1.978621	2.124077	0.033664	NA
1.05E+08	LOC10537(	2.257429	-3.5144	1.781442	-1.97278	0.04852	NA
64843	ISL2	7.059308	2.040265	0.993962	2.05266	0.040106	NA
1.05E+08	LOC10537(	3.602092	-3.26293	1.486479	-2.19507	0.028159	NA
643707	GOLGA6L4	5.186623	-2.32921	1.045492	-2.22786	0.02589	NA
1.03E+08	LOC10272·	2.479263	-4.71174	1.732775	-2.71919	0.006544	NA
374650	GOLGA6L5I	6.41488	-1.86767	0.921937	-2.02581	0.042784	NA
283951	C16orf91	2.521803	3.789478	1.711492	2.214137	0.026819	NA

146110	RPL7P47	6.460585	2.927604	1.253976	2.334658	0.019561	NA
79724	ZNF768	4.938391	3.190028	1.247566	2.557001	0.010558	NA
4501	MT1X	3.354882	5.232514	1.696039	3.085137	0.002035	NA
29070	CCDC113	5.691575	-2.15506	1.066361	-2.02095	0.043285	NA
79007	DBNDD1	5.663408	2.334888	1.163141	2.007399	0.044707	NA
1.05E+08	LOC10537[illegible]	2.135023	-4.51845	1.807996	-2.49915	0.012449	NA
10462	CLEC10A	7.019107	3.399365	1.11477	3.049386	0.002293	NA
1.02E+08	LOC10192[illegible]	3.276101	4.203412	1.589373	2.644698	0.008176	NA
1.05E+08	LOC10537[illegible]	3.480197	2.656704	1.293255	2.054276	0.039949	NA
8557	TCAP	3.217914	3.130395	1.544979	2.026173	0.042747	NA
317719	KLHL10	4.337469	-2.38546	1.194901	-1.99637	0.045894	NA
8639	AOC3	4.6386	2.644166	1.230055	2.149632	0.031584	NA
2535	FZD2	7.004084	2.617772	0.950681	2.753576	0.005895	NA
342538	NACA2	3.872002	2.744687	1.379514	1.989605	0.046634	NA
9021	SOCS3	6.154898	4.173184	1.320562	3.160158	0.001577	NA
729602	NPIPB1P	6.761429	-2.69281	1.039314	-2.59095	0.009571	NA
5366	PMAIP1	4.771277	2.369262	1.092958	2.167752	0.030178	NA
69	ACTBP9	3.265562	-3.06531	1.532072	-2.00076	0.045419	NA
400668	PRSS57	4.851638	-2.70216	1.286635	-2.10017	0.035714	NA
1E+08	ZGLP1	2.154937	4.618039	1.818391	2.53963	0.011097	NA
401904	RPL23AP2	6.28124	2.115845	0.960078	2.203827	0.027537	NA
1.02E+08	LOC10192[illegible]	2.432868	3.714417	1.741206	2.133244	0.032905	NA
84964	ALKBH6	6.149303	2.722644	1.039715	2.618644	0.008828	NA
1556	CYP2B7P	1.761042	4.335921	1.92539	2.251971	0.024324	NA
1.01E+08	LOC10050[illegible]	2.414941	3.717371	1.872451	1.985297	0.047111	NA
147650	SPACA6P	7.231312	2.53387	0.916949	2.763369	0.005721	NA
400720	ZNF772	5.451033	2.318634	1.077239	2.152385	0.031367	NA
10520	ZNF211	3.969206	2.643981	1.33097	1.986507	0.046977	NA
1.05E+08	LOC10537[illegible]	7.222655	2.237753	0.934847	2.393711	0.016679	NA
85449	KIAA1755	3.716117	-2.56928	1.305297	-1.96835	0.049028	NA
671	BPI	3.021598	4.08966	1.638801	2.495519	0.012577	NA
6285	S100B	6.139694	2.497897	1.197762	2.08547	0.037027	NA
6576	SLC25A1	5.280262	2.207736	1.110422	1.988196	0.04679	NA
421	ARVCF	2.40389	-3.60629	1.77606	-2.0305	0.042306	NA
402055	SRRD	6.095709	2.377421	1.04211	2.281354	0.022528	NA
1.05E+08	LOC10537[illegible]	2.191734	4.652079	2.023724	2.298772	0.021518	NA
440836	ODF3B	5.877838	2.397024	1.059051	2.26337	0.023613	NA
8908	GYG2	2.504286	3.780366	1.75402	2.155258	0.031142	NA
349408	TLR8-AS1	5.912165	2.414198	1.061405	2.27453	0.022934	NA
1.02E+08	LOC10192[illegible]	5.181317	-2.75856	1.158656	-2.38083	0.017274	NA
2623	GATA1	4.615987	2.575634	1.208152	2.131879	0.033017	NA
645251	CBX1P1	1.656682	4.239121	1.990971	2.129173	0.03324	NA
1.02E+08	LOC10192[illegible]	2.195716	4.623041	1.85614	2.490675	0.01275	NA
1.05E+08	LOC10537[illegible]	5.830262	-1.94347	0.969323	-2.00498	0.044965	NA
57801	HES4	3.310502	3.174884	1.493705	2.12551	0.033544	NA
1.05E+08	LOC10537[illegible]	1.489762	4.086153	1.994672	2.048534	0.040508	NA
343381	NBPF2P	2.157936	3.55268	1.737486	2.044724	0.040882	NA
400963	RPS2P17	3.155599	4.112111	1.618838	2.540162	0.01108	NA
129303	TMEM150A	3.770726	4.388286	1.561668	2.81	0.004954	NA
1.01E+08	LOC10050[illegible]	3.034174	-3.03817	1.519881	-1.99895	0.045614	NA
7293	TNFRSF4	3.146444	5.16476	1.62821	3.172048	0.001514	NA
1.05E+08	LOC10537[illegible]	4.38383	-2.4854	1.238732	-2.00641	0.044813	NA
79727	LIN28A	2.652978	-3.75183	1.671613	-2.24444	0.024804	NA
55139	ANKZF1	5.898049	1.863313	0.938523	1.985367	0.047104	NA

1.05E+08	LOC10537:	2.59629	-3.72769	1.68255	-2.2155	0.026726	NA
729083	LOC72908:	4.455035	2.469347	1.221796	2.02108	0.043272	NA
1.03E+08	LOC10272	5.454871	2.664145	1.287301	2.069559	0.038494	NA
8692	HYAL2	2.588628	3.840246	1.740587	2.206293	0.027363	NA
