# Supplementary material for: Genomic Expression Profiling and Bioinformatics Analysis of Chronic Recurrent Multifocal Osteomyelitis
Source: Biomed Res Int. 2021 Feb 9;2021:6898093. doi: 10.1155/2021/6898093 (PMC7888306; doi:10.1155/2021/6898093)
Supplement: Supplementary 7 — Supplementary File 2 The detailed results of the GO enrichment analyses of all the DEGs. [file 6898093.f7.pdf]

Terms	Counts	GeneRati(	BgRatio	pValue	FDR	foldEnri(	geneID	geneSymb(	Category
GO:00198(	84	84/1117	341/2061(	9.77E-33	3.07E-29	4.545166	ENSG0000(	PSMB1/NA/	GO_BP
GO:00480(	83	83/1117	348/2061(	2.84E-31	4.91E-28	4.400719	ENSG0000(	PSMB1/NA/	GO_BP
GO:00024'	26	26/1117	60/20610	1.08E-17	3.51E-15	7.995524	ENSG0000(	TAP2/NA/I	GO_BP
GO:00198(	27	27/1117	77/20610	1.55E-15	3.10E-13	6.469904	ENSG0000(	TAP2/NA/I	GO_BP
GO:00229(	42	42/1117	203/2061(	4.20E-14	4.95E-12	3.817491	ENSG0000(	NDUFA7/C(	GO_BP
GO:00064:	41	41/1117	222/2061(	4.24E-12	4.31E-10	3.407656	ENSG0000(	RPL38/RPI	GO_BP
GO:00022:	58	58/1117	406/2061(	1.32E-11	1.24E-09	2.635887	ENSG0000(	PSMB1/NA/	GO_BP
GO:00025(	36	36/1117	186/2061(	2.12E-11	1.87E-09	3.571202	ENSG0000(	IFI30/HL/	GO_BP
GO:00159(	47	47/1117	304/2061(	7.85E-11	6.67E-09	2.852654	ENSG0000(	NDUFA7/C(	GO_BP
GO:00902(	8	8/1117	13/20610	7.32E-08	4.57E-06	11.35459	ENSG0000(	AIF1/NA/I	GO_BP
GO:00902'	8	8/1117	13/20610	7.32E-08	4.57E-06	11.35459	ENSG0000(	AIF1/NA/I	GO_BP
GO:00218:	10	10/1117	22/20610	7.47E-08	4.61E-06	8.386913	ENSG0000(	MBOAT7/N/	GO_BP
GO:00019:	22	22/1117	112/2061(	1.26E-07	7.42E-06	3.624345	ENSG0000(	HLA-A/NA/	GO_BP
GO:00326:	21	21/1117	104/2061(	1.48E-07	8.44E-06	3.725725	ENSG0000(	TMSB4X/F/	GO_BP
GO:00313(	23	23/1117	124/2061(	1.96E-07	1.05E-05	3.422402	ENSG0000(	HLA-A/NA/	GO_BP
GO:00725(	41	41/1117	316/2061(	2.02E-07	1.07E-05	2.393986	ENSG0000(	KLF2/AIF	GO_BP
GO:00018:	44	44/1117	365/2061(	5.91E-07	2.71E-05	2.224255	ENSG0000(	TMSB4X/KI	GO_BP
GO:00326:	29	29/1117	197/2061(	9.28E-07	3.95E-05	2.716168	ENSG0000(	KLF2/AIF	GO_BP
GO:00508(	47	47/1117	409/2061(	9.83E-07	4.14E-05	2.12031	ENSG0000(	AIF1/NA/I	GO_BP
GO:00019:	20	20/1117	111/2061(	1.95E-06	7.59E-05	3.324542	ENSG0000(	HLA-A/NA/	GO_BP
GO:00341(	24	24/1117	151/2061(	2.01E-06	7.76E-05	2.932642	ENSG0000(	KLF2/B2M/	GO_BP
GO:00512:	52	52/1117	495/2061(	3.94E-06	0.000143	1.938309	ENSG0000(	PANX2/UBI	GO_BP
GO:00149(	6	6/1117	10/20610	4.35E-06	0.00015	11.07073	ENSG0000(	AIF1/NA/I	GO_BP
GO:00326:	15	15/1117	71/20610	4.86E-06	0.000164	3.898143	ENSG0000(	TYROBP/I:	GO_BP
GO:00434(	29	29/1117	214/2061(	5.06E-06	0.000167	2.500397	ENSG0000(	PSMB1/NA/	GO_BP
GO:19035:	50	50/1117	476/2061(	6.04E-06	0.000192	1.938152	ENSG0000(	SDCBP/PAI	GO_BP
GO:00109(	46	46/1117	424/2061(	6.08E-06	0.000192	2.001782	ENSG0000(	PSMB1/NA/	GO_BP
GO:00506(	37	37/1117	312/2061(	6.69E-06	0.000209	2.188124	ENSG0000(	TMSB4X/P/	GO_BP
GO:00075(	13	13/1117	57/20610	8.54E-06	0.000256	4.20817	ENSG0000(	MMP9/CALI	GO_BP
GO:00321(	42	42/1117	391/2061(	1.94E-05	0.000507	1.981971	ENSG0000(	TMSB4X/S	GO_BP
GO:00717(	28	28/1117	218/2061(	2.02E-05	0.000523	2.36988	ENSG0000(	ADAM8/TYI	GO_BP
GO:19019'	7	7/1117	19/20610	3.82E-05	0.000902	6.797814	ENSG0000(	PPP1R10/I	GO_BP
GO:00305(	31	31/1117	263/2061(	4.09E-05	0.000956	2.174857	ENSG0000(	S100A8/A	GO_BP
GO:00071(	21	21/1117	147/2061(	4.49E-05	0.001035	2.635887	ENSG0000(	PSMB1/NA/	GO_BP
GO:00026(	38	38/1117	354/2061(	4.81E-05	0.00108	1.980638	ENSG0000(	HLA-A/NA/	GO_BP
GO:00456(	12	12/1117	59/20610	6.35E-05	0.001307	3.752788	ENSG0000(	B2M/NA/I:	GO_BP
GO:00025'	9	9/1117	35/20610	7.67E-05	0.001553	4.744596	ENSG0000(	WAS/CD74/	GO_BP
GO:00714(	38	38/1117	362/2061(	7.77E-05	0.001567	1.936867	ENSG0000(	LAMP2/GAI	GO_BP
GO:00467:	7	7/1117	21/20610	8.01E-05	0.001585	6.150403	ENSG0000(	LAMP2/GA/	GO_BP
GO:00704(	44	44/1117	443/2061(	8.15E-05	0.001585	1.832626	ENSG0000(	PSMB1/NA/	GO_BP
GO:00015(	8	8/1117	28/20610	8.52E-05	0.001631	5.271774	ENSG0000(	BATF2/BCI	GO_BP
GO:00016(	41	41/1117	407/2061(	0.000103	0.001916	1.858721	ENSG0000(	PSMB1/NA/	GO_BP
GO:00341:	11	11/1117	55/20610	0.000149	0.002618	3.690242	ENSG0000(	ARRB2/LII	GO_BP
GO:00516:	15	15/1117	94/20610	0.000154	0.002642	2.944342	ENSG0000(	FTL/TMSB(	GO_BP
GO:00607(	13	13/1117	75/20610	0.000179	0.002987	3.198209	ENSG0000(	FADD/TLR:	GO_BP
GO:00607(	7	7/1117	24/20610	0.000207	0.003337	5.381603	ENSG0000(	DDR1/NA/I	GO_BP
GO:00613'	7	7/1117	24/20610	0.000207	0.003337	5.381603	ENSG0000(	DDR1/NA/I	GO_BP
GO:00316(	31	31/1117	289/2061(	0.000234	0.003709	1.979195	ENSG0000(	LAMP2/GAI	GO_BP
GO:00329(	35	35/1117	344/2061(	0.000267	0.004075	1.877303	ENSG0000(	AIF1/NA/I	GO_BP
GO:00620:	46	46/1117	497/2061(	0.000291	0.004413	1.707758	ENSG0000(	PSMB1/NA/	GO_BP
GO:00026(	38	38/1117	387/2061(	0.000305	0.00457	1.811747	ENSG0000(	AIF1/NA/I	GO_BP
GO:00326(	6	6/1117	19/20610	0.000368	0.00538	5.826697	ENSG0000(	LILRA2/N/	GO_BP
GO:20010:	15	15/1117	102/2061(	0.000386	0.005571	2.713413	ENSG0000(	KLF2/AIF	GO_BP

GO:004218	29	29/1117	272/20610	0.000405	0.005755	1.967224	ENSG0000	PSMB1/NA/	GO_BP
GO:000645	10	10/1117	52/20610	0.000414	0.005824	3.548309	ENSG0000	HSPA6/CD7	GO_BP
GO:007060	22	22/1117	185/20610	0.000451	0.006273	2.194198	ENSG0000	BST2/AIF	GO_BP
GO:004593	7	7/1117	27/20610	0.00046	0.006337	4.783647	ENSG0000	RGS2/SRI/	GO_BP
GO:003134	25	25/1117	223/20610	0.000472	0.006487	2.068521	ENSG0000	HLA-A/NA/	GO_BP
GO:005160	29	29/1117	276/20610	0.000515	0.006934	1.938714	ENSG0000	IFITM2/RI	GO_BP
GO:004202	10	10/1117	54/20610	0.000568	0.007548	3.41689	ENSG0000	HSPA6/B2M	GO_BP
GO:003261	19	19/1117	152/20610	0.000582	0.007675	2.306401	ENSG0000	PANX2/CAI	GO_BP
GO:003052	32	32/1117	319/20610	0.000613	0.00795	1.850905	ENSG0000	PIM1/NFKI	GO_BP
GO:000961	36	36/1117	374/20610	0.000628	0.008076	1.776052	ENSG0000	IFITM2/RI	GO_BP
GO:003260	19	19/1117	153/20610	0.000631	0.008101	2.291327	ENSG0000	DHX58/ISG	GO_BP
GO:007060	30	30/1117	293/20610	0.00064	0.00819	1.889202	ENSG0000	BST2/AIF	GO_BP
GO:001474	6	6/1117	21/20610	0.000671	0.008529	5.271774	ENSG0000	AIF1/NA/N	GO_BP
GO:000191	23	23/1117	203/20610	0.000673	0.008536	2.090531	ENSG0000	HLA-A/NA/	GO_BP
GO:003164	32	32/1117	322/20610	0.000718	0.009032	1.83366	ENSG0000	LAMP2/PIM	GO_BP
GO:000230	21	21/1117	179/20610	0.000719	0.009032	2.164667	ENSG0000	BST2/B2M/	GO_BP
GO:003803	13	13/1117	87/20610	0.000792	0.009848	2.757077	ENSG0000	MCL1/FADI	GO_BP
GO:003160	6	6/1117	22/20610	0.000881	0.010676	5.032148	ENSG0000	CARD16/L	GO_BP
GO:000252	5	5/1117	15/20610	0.000881	0.010676	6.150403	ENSG0000	S100A8/AI	GO_BP
GO:005123	5	5/1117	15/20610	0.000881	0.010676	6.150403	ENSG0000	FTL/S100A	GO_BP
GO:009055	14	14/1117	99/20610	0.000887	0.010676	2.609262	ENSG0000	SLC25A5/I	GO_BP
GO:007121	29	29/1117	286/20610	0.00091	0.010928	1.870927	ENSG0000	CARD16/T	GO_BP
GO:005092	19	19/1117	159/20610	0.001011	0.012053	2.204861	ENSG0000	TMSB4X/A	GO_BP
GO:000268	19	19/1117	160/20610	0.001091	0.012822	2.191081	ENSG0000	AIF1/NA/N	GO_BP
GO:007267	17	17/1117	137/20610	0.001201	0.013933	2.289566	ENSG0000	AIF1/NA/N	GO_BP
GO:000257	6	6/1117	24/20610	0.001448	0.016067	4.612802	ENSG0000	HLA-DOB/I	GO_BP
GO:006107	11	11/1117	71/20610	0.00145	0.016067	2.858638	ENSG0000	HSPA6/CR	GO_BP
GO:000270	19	19/1117	166/20610	0.001687	0.018377	2.111885	ENSG0000	B2M/NA/S	GO_BP
GO:005109	37	37/1117	410/20610	0.001713	0.018616	1.665109	ENSG0000	TMSB4X/C	GO_BP
GO:190340	17	17/1117	142/20610	0.001782	0.019293	2.208948	ENSG0000	KLF2/AIF	GO_BP
GO:000220	11	11/1117	73/20610	0.001827	0.019528	2.780319	ENSG0000	AIF1/NA/N	GO_BP
GO:005123	33	33/1117	355/20610	0.001842	0.019655	1.715183	ENSG0000	FTL/TMSB4	GO_BP
GO:000709	21	21/1117	193/20610	0.001876	0.019931	2.007644	ENSG0000	DUSP1/MUS	GO_BP
GO:005170	27	27/1117	273/20610	0.001929	0.020456	1.824845	ENSG0000	IFITM2/D	GO_BP
GO:009752	25	25/1117	249/20610	0.00227	0.023222	1.852531	ENSG0000	S100A8/A	GO_BP
GO:000220	10	10/1117	65/20610	0.00248	0.025065	2.838647	ENSG0000	FADD/SIT	GO_BP
GO:190370	23	23/1117	224/20610	0.002506	0.025178	1.894544	ENSG0000	ADAM8/TYI	GO_BP
GO:000268	24	24/1117	238/20610	0.002595	0.026027	1.860626	ENSG0000	AIF1/NA/N	GO_BP
GO:190197	8	8/1117	45/20610	0.00261	0.026072	3.280215	ENSG0000	DUSP1/PPI	GO_BP
GO:009880	15	15/1117	123/20610	0.002697	0.026792	2.250147	ENSG0000	MGST3/ALO	GO_BP
GO:190353	26	26/1117	266/20610	0.002721	0.026887	1.803502	ENSG0000	TMSB4X/C	GO_BP
GO:003437	7	7/1117	36/20610	0.002822	0.027658	3.587735	ENSG0000	ARF1/ZDHI	GO_BP
GO:004327	28	28/1117	295/20610	0.002922	0.028532	1.751301	ENSG0000	TMSB4X/C	GO_BP
GO:000962	10	10/1117	67/20610	0.003117	0.029705	2.753912	ENSG0000	S100A8/S	GO_BP
GO:004518	14	14/1117	113/20610	0.003181	0.030268	2.28599	ENSG0000	TMSB4X/T	GO_BP
GO:000695	30	30/1117	327/20610	0.003509	0.032374	1.692771	ENSG0000	S100A8/L	GO_BP
GO:004545	11	11/1117	80/20610	0.003814	0.034759	2.537041	ENSG0000	GPX1/NCF4	GO_BP
GO:000750	21	21/1117	206/20610	0.004095	0.036676	1.880948	ENSG0000	RGS2/MMP9	GO_BP
GO:004667	31	31/1117	347/20610	0.004498	0.039746	1.648379	ENSG0000	KLF2/S100	GO_BP
GO:003220	12	12/1117	94/20610	0.004769	0.041713	2.355473	ENSG0000	PPP1R10/I	GO_BP
GO:003590	23	23/1117	236/20610	0.004795	0.041856	1.798211	ENSG0000	CTDSP2/HS	GO_BP
GO:005092	24	24/1117	250/20610	0.004855	0.04224	1.771316	ENSG0000	TMSB4X/A	GO_BP
GO:006190	11	11/1117	83/20610	0.005074	0.044069	2.445341	ENSG0000	AIF1/NA/N	GO_BP
GO:000940	8	8/1117	50/20610	0.005121	0.044258	2.952193	ENSG0000	GMPR/NFKI	GO_BP

GO:00092(	26	26/1117	279/2061(	0.005132	0.044277	1.719467	ENSG0000(	GMPR/HSP/	GO_BP
GO:00326	11	11/1117	84/20610	0.00556	0.047339	2.41623	ENSG0000(	IRF5/PRK(	GO_BP
GO:00015	39	39/1117	470/2061(	0.005589	0.04748	1.531058	ENSG0000(	SDCBP/S1(	GO_BP
GO:00510	27	27/1117	295/2061(	0.005595	0.04748	1.688755	ENSG0000(	TMSB4X/C/	GO_BP
GO:00486(	10	10/1117	74/20610	0.006426	0.053742	2.493407	ENSG0000(	AIF1/NA/	GO_BP
GO:00027(	8	8/1117	52/20610	0.006528	0.054416	2.838647	ENSG0000(	BST2/HLA-	GO_BP
GO:00457	12	12/1117	98/20610	0.006664	0.055464	2.259332	ENSG0000(	ATRAID/C	GO_BP
GO:00325(	10	10/1117	75/20610	0.007064	0.057865	2.460161	ENSG0000(	TMSB4X/T	GO_BP
GO:00027(	21	21/1117	218/2061(	0.007767	0.062339	1.77741	ENSG0000(	BST2/B2M,	GO_BP
GO:00030	14	14/1117	125/2061(	0.007827	0.062718	2.066535	ENSG0000(	AQP3/PSA	GO_BP
GO:00516(	8	8/1117	54/20610	0.008208	0.064682	2.733512	ENSG0000(	AIF1/NA/	GO_BP
GO:00457	35	35/1117	426/2061(	0.009648	0.074329	1.515944	ENSG0000(	AIF1/NA/	GO_BP
GO:00900	5	5/1117	25/20610	0.009936	0.076099	3.690242	ENSG0000(	HSPA1B/N/	GO_BP
GO:00316(	6	6/1117	35/20610	0.010513	0.079574	3.163064	ENSG0000(	CARD16/L	GO_BP
GO:00326	6	6/1117	35/20610	0.010513	0.079574	3.163064	ENSG0000(	IRF4/LIL	GO_BP
GO:00421(	6	6/1117	35/20610	0.010513	0.079574	3.163064	ENSG0000(	TSPO/TME	GO_BP
GO:00075	31	31/1117	369/2061(	0.010609	0.080187	1.550102	ENSG0000(	DGKI/PRK(	GO_BP
GO:00313	27	27/1117	313/2061(	0.011851	0.087986	1.591638	ENSG0000(	PFN1/ARP(	GO_BP
GO:00420	6	6/1117	36/20610	0.012051	0.088755	3.075201	ENSG0000(	HLX/CD74,	GO_BP
GO:00510	18	18/1117	188/2061(	0.01382	0.098703	1.766605	ENSG0000(	S100A8/C/	GO_BP
GO:00364	11	11/1117	96/20610	0.014718	0.103547	2.114201	ENSG0000(	MCL1/PSA	GO_BP
GO:00903	16	16/1117	162/2061(	0.014927	0.104733	1.822342	ENSG0000(	UBL5/OAZ	GO_BP
GO:20010	11	11/1117	97/20610	0.015815	0.109481	2.092405	ENSG0000(	CDKN2D/P	GO_BP
GO:00326(	12	12/1117	110/2061(	0.016036	0.110715	2.012859	ENSG0000(	S100A8/A	GO_BP
GO:00301(	17	17/1117	178/2061(	0.016778	0.114922	1.762194	ENSG0000(	DGKI/PRK(	GO_BP
GO:00508(	22	22/1117	249/2061(	0.016846	0.114932	1.630227	ENSG0000(	TYROBP/P	GO_BP
GO:00510	27	27/1117	325/2061(	0.018536	0.123531	1.53287	ENSG0000(	S100A8/C/	GO_BP
GO:00459	7	7/1117	51/20610	0.019416	0.127497	2.532519	ENSG0000(	RGS2/PRO	GO_BP
GO:00325(	6	6/1117	40/20610	0.019787	0.127976	2.767681	ENSG0000(	CHMP2A/R	GO_BP
GO:00026	20	20/1117	224/2061(	0.01982	0.127976	1.647429	ENSG0000(	TYROBP/Z(	GO_BP
GO:00607	20	20/1117	224/2061(	0.01982	0.127976	1.647429	ENSG0000(	CARD16/F/	GO_BP
GO:00302	20	20/1117	227/2061(	0.02251	0.142162	1.625657	ENSG0000(	ATRAID/C	GO_BP
GO:00023	13	13/1117	129/2061(	0.022854	0.14416	1.859424	ENSG0000(	STX4/TCF	GO_BP
GO:00509	9	9/1117	77/20610	0.023102	0.145368	2.156635	ENSG0000(	AIF1/NA/	GO_BP
GO:00098	3	3/1117	12/20610	0.024174	0.147808	4.612802	ENSG0000(	HMGN1/BC	GO_BP
GO:00106	5	5/1117	31/20610	0.024333	0.148083	2.976001	ENSG0000(	CALM3/SR	GO_BP
GO:00601	8	8/1117	66/20610	0.025739	0.153932	2.23651	ENSG0000(	RGS2/ADC	GO_BP
GO:00315	17	17/1117	188/2061(	0.026971	0.159854	1.66846	ENSG0000(	MUS81/PP	GO_BP
GO:00550	5	5/1117	32/20610	0.027592	0.162388	2.883001	ENSG0000(	ITGB2/CD	GO_BP
GO:00016	21	21/1117	252/2061(	0.03369	0.189052	1.537601	ENSG0000(	ATRAID/C	GO_BP
GO:00714(	5	5/1117	34/20610	0.034907	0.194201	2.713413	ENSG0000(	ITGB2/CD	GO_BP
GO:00702(	8	8/1117	70/20610	0.03509	0.194592	2.10871	ENSG0000(	FADD/PPI	GO_BP
GO:00985	3	3/1117	14/20610	0.036905	0.202916	3.95383	ENSG0000(	TLR2/TLR	GO_BP
GO:00303(	18	18/1117	210/2061(	0.036998	0.203217	1.581532	ENSG0000(	BST2/RGS	GO_BP
GO:00429	4	4/1117	24/20610	0.038341	0.208603	3.075201	ENSG0000(	TMSB4X/N	GO_BP
GO:00486(	12	12/1117	125/2061(	0.038767	0.210465	1.771316	ENSG0000(	MMP9/AIF	GO_BP
GO:00712	27	27/1117	348/2061(	0.039341	0.212043	1.431559	ENSG0000(	CARD16/F/	GO_BP
GO:01040(	27	27/1117	348/2061(	0.039341	0.212043	1.431559	ENSG0000(	CARD16/F/	GO_BP
GO:00098	35	35/1117	474/2061(	0.039853	0.214067	1.362431	ENSG0000(	RILP/NA/	GO_BP
GO:00716	12	12/1117	127/2061(	0.042963	0.227308	1.743421	ENSG0000(	AIF1/NA/	GO_BP
GO:00433(	5	5/1117	36/20610	0.043316	0.22825	2.562668	ENSG0000(	SRF/CD74,	GO_BP
GO:00323	19	19/1117	229/2061(	0.043363	0.22825	1.530886	ENSG0000(	UBL5/OAZ	GO_BP
GO:00466	4	4/1117	25/20610	0.043752	0.228885	2.952193	ENSG0000(	JUNB/BSG,	GO_BP
GO:00096	18	18/1117	216/2061(	0.046602	0.238529	1.537601	ENSG0000(	TXNIP/NF	GO_BP

GO:00430	5	5/1117	37/20610	0.047937	0.244156	2.493407	ENSG0000	GMPR/PNP	GO_BP
GO:00347	13	13/1117	144/2061	0.049205	0.249635	1.665734	ENSG0000	RGS2/MMP	GO_BP
GO:00346	4	4/1117	26/20610	0.049567	0.250212	2.838647	ENSG0000	TNFAIP2/	GO_BP
GO:00709	32	32/1117	436/2061	0.050958	0.256025	1.354217	ENSG0000	MCL1/ADA	GO_BP
GO:00097	9	9/1117	89/20610	0.051665	0.259019	1.865853	ENSG0000	HMGN1/PY	GO_BP
GO:00059	24	24/1117	311/2061	0.052303	0.259019	1.423887	ENSG0000	IRS2/WDR	GO_BP
GO:00017	3	3/1117	16/20610	0.052414	0.259019	3.459602	ENSG0000	HCAR2/CC	GO_BP
GO:19021	3	3/1117	16/20610	0.052414	0.259019	3.459602	ENSG0000	CHMP2A/D	GO_BP
GO:00321	31	31/1117	423/2061	0.054871	0.268911	1.352216	ENSG0000	CARD16/A	GO_BP
GO:00439	19	19/1117	236/2061	0.055551	0.271218	1.485479	ENSG0000	IFITM2/B	GO_BP
GO:00458	16	16/1117	191/2061	0.055934	0.271807	1.545651	ENSG0000	HNRNPK/B	GO_BP
GO:00028	7	7/1117	64/20610	0.057305	0.27614	2.018101	ENSG0000	CARD16/D	GO_BP
GO:00461	7	7/1117	64/20610	0.057305	0.27614	2.018101	ENSG0000	TSPO/APR	GO_BP
GO:00448	3	3/1117	17/20610	0.061164	0.289091	3.256096	ENSG0000	OPRL1/NA	GO_BP
GO:00512	3	3/1117	17/20610	0.061164	0.289091	3.256096	ENSG0000	CALM3/SR	GO_BP
GO:19002	3	3/1117	17/20610	0.061164	0.289091	3.256096	ENSG0000	SRF/HMGC	GO_BP
GO:00507	19	19/1117	239/2061	0.061447	0.289375	1.466832	ENSG0000	BST2/DHX	GO_BP
GO:00512	19	19/1117	239/2061	0.061447	0.289375	1.466832	ENSG0000	TMSB4X/C	GO_BP
GO:00219	12	12/1117	135/2061	0.062876	0.293439	1.640107	ENSG0000	RHOA/SLC	GO_BP
GO:00486	16	16/1117	195/2061	0.064929	0.301123	1.513945	ENSG0000	MMP9/AIF	GO_BP
GO:00335	4	4/1117	29/20610	0.069416	0.31711	2.544994	ENSG0000	ZNF703/G	GO_BP
GO:00486	16	16/1117	197/2061	0.069773	0.318463	1.498575	ENSG0000	MMP9/AIF	GO_BP
GO:00024	7	7/1117	67/20610	0.069984	0.318612	1.927738	ENSG0000	PSMB4/KD	GO_BP
GO:00342	6	6/1117	54/20610	0.070886	0.319327	2.050134	ENSG0000	CDC42SE1	GO_BP
GO:19049	19	19/1117	245/2061	0.074504	0.333816	1.43091	ENSG0000	TMSB4X/C	GO_BP
GO:00336	4	4/1117	30/20610	0.076813	0.339247	2.460161	ENSG0000	ATRAID/R	GO_BP
GO:00723	8	8/1117	83/20610	0.080289	0.349975	1.77843	ENSG0000	CARM1/PR	GO_BP
GO:00105	3	3/1117	19/20610	0.080525	0.349975	2.913349	ENSG0000	CALM3/SR	GO_BP
GO:00970	10	10/1117	112/2061	0.082448	0.356541	1.647429	ENSG0000	HNRNPK/A	GO_BP
GO:00028	14	14/1117	172/2061	0.084456	0.362024	1.501843	ENSG0000	CARD16/D	GO_BP
GO:00309	9	9/1117	99/20610	0.088212	0.373983	1.677383	ENSG0000	CALM3/CO	GO_BP
GO:00425	16	16/1117	204/2061	0.088601	0.375322	1.447154	ENSG0000	LAMP2/GA	GO_BP
GO:00325	28	28/1117	395/2061	0.089469	0.378384	1.307934	ENSG0000	TMSB4X/T	GO_BP
GO:00026	16	16/1117	205/2061	0.091531	0.382734	1.440094	ENSG0000	BST2/DHX	GO_BP
GO:00508	7	7/1117	72/20610	0.094538	0.391516	1.793868	ENSG0000	AQP3/ADC	GO_BP
GO:00076	13	13/1117	160/2061	0.094804	0.391529	1.499161	ENSG0000	DGKI/EIF	GO_BP
GO:00620	13	13/1117	160/2061	0.094804	0.391529	1.499161	ENSG0000	TMSB4X/I	GO_BP
GO:00224	24	24/1117	334/2061	0.097705	0.396341	1.325835	ENSG0000	AIF1/NA/	GO_BP
GO:00344	2	2/1117	10/20610	0.098924	0.396341	3.690242	ENSG0000	APOBR/LR	GO_BP
GO:00019	8	8/1117	87/20610	0.09901	0.396341	1.696663	ENSG0000	ARRB2/HL	GO_BP
GO:00860	6	6/1117	59/20610	0.099066	0.396341	1.876394	ENSG0000	CALM3/SR	GO_BP
GO:19004	6	6/1117	59/20610	0.099066	0.396341	1.876394	ENSG0000	PSAP/GPX	GO_BP
GO:00070	19	19/1117	255/2061	0.10016	0.4001	1.374796	ENSG0000	CDKN2D/D	GO_BP
GO:00900	23	23/1117	319/2061	0.100427	0.400856	1.330338	ENSG0000	AIF1/NA/	GO_BP
GO:20010	4	4/1117	33/20610	0.101229	0.402817	2.23651	ENSG0000	RGS2/PSA	GO_BP
GO:00022	5	5/1117	46/20610	0.102006	0.404688	2.005566	ENSG0000	FFAR2/DE	GO_BP
GO:00550	3	3/1117	21/20610	0.102167	0.404688	2.635887	ENSG0000	MT-ATP6/	GO_BP
GO:00512	29	29/1117	419/2061	0.106098	0.417705	1.277053	ENSG0000	BST2/AIF	GO_BP
GO:00197	12	12/1117	148/2061	0.10661	0.419084	1.496044	ENSG0000	S100A8/L	GO_BP
GO:00459	20	20/1117	274/2061	0.108904	0.426942	1.346804	ENSG0000	BST2/RGS	GO_BP
GO:00481	8	8/1117	89/20610	0.109217	0.426942	1.658536	ENSG0000	S100A6/F	GO_BP
GO:00060	4	4/1117	34/20610	0.110072	0.427186	2.17073	ENSG0000	SDHC/FH/	GO_BP
GO:00219	4	4/1117	34/20610	0.110072	0.427186	2.17073	ENSG0000	SRF/SEMA	GO_BP
GO:00432	14	14/1117	180/2061	0.111279	0.430262	1.435094	ENSG0000	RGS2/MMP	GO_BP

GO:190288	6	6/1117	61/20610	0.111775	0.431214	1.814873	ENSG0000(	PSAP/GPX	GO_BP
GO:000940	16	16/1117	212/2061(	0.113749	0.434354	1.392544	ENSG0000(	HSPA6/HS	GO_BP
GO:190560	2	2/1117	11/20610	0.116712	0.435046	3.354765	ENSG0000(	ABHD17A/	GO_BP
GO:190594	2	2/1117	11/20610	0.116712	0.435046	3.354765	ENSG0000(	RETN/CIT	GO_BP
GO:005135	6	6/1117	62/20610	0.118425	0.439538	1.785601	ENSG0000(	CALM3/GN	GO_BP
GO:009890	4	4/1117	35/20610	0.119246	0.43975	2.10871	ENSG0000(	KCNE3/BI	GO_BP
GO:000697	8	8/1117	91/20610	0.119976	0.441147	1.622084	ENSG0000(	TSC22D4/	GO_BP
GO:003134	8	8/1117	91/20610	0.119976	0.441147	1.622084	ENSG0000(	ARRB2/HL	GO_BP
GO:004390	20	20/1117	278/2061(	0.120774	0.442868	1.327425	ENSG0000(	IFITM2/B	GO_BP
GO:000975	19	19/1117	262/2061(	0.12108	0.44336	1.338065	ENSG0000(	ARRB2/RH	GO_BP
GO:000301	33	33/1117	492/2061(	0.121528	0.444518	1.237581	ENSG0000(	RGS2/AIF	GO_BP
GO:000652	29	29/1117	426/2061(	0.122717	0.448088	1.256068	ENSG0000(	PSMB1/NA	GO_BP
GO:002154	15	15/1117	199/2061(	0.123552	0.449873	1.390795	ENSG0000(	KDM6B/RH	GO_BP
GO:003297	28	28/1117	410/2061(	0.124103	0.451561	1.260083	ENSG0000(	TMSB4X/B	GO_BP
GO:004341	21	21/1117	296/2061(	0.126325	0.453521	1.309038	ENSG0000(	SDF2/TME	GO_BP
GO:004213	14	14/1117	184/2061(	0.126391	0.453521	1.403896	ENSG0000(	KLF2/AIF	GO_BP
GO:000189	4	4/1117	36/20610	0.128737	0.459711	2.050134	ENSG0000(	JUNB/BSG	GO_BP
GO:004814	4	4/1117	36/20610	0.128737	0.459711	2.050134	ENSG0000(	FTH1/EMD	GO_BP
GO:009700	4	4/1117	36/20610	0.128737	0.459711	2.050134	ENSG0000(	FLCN/MET	GO_BP
GO:007142	11	11/1117	138/2061(	0.129213	0.460776	1.470749	ENSG0000(	SRSF1/U2	GO_BP
GO:004819	7	7/1117	78/20610	0.1295	0.461481	1.655878	ENSG0000(	SEC16A/R	GO_BP
GO:006201	9	9/1117	108/2061(	0.131497	0.466673	1.537601	ENSG0000(	PPIF/FLC	GO_BP
GO:000240	2	2/1117	12/20610	0.135219	0.467407	3.075201	ENSG0000(	CLEC4A/C	GO_BP
GO:006045	2	2/1117	12/20610	0.135219	0.467407	3.075201	ENSG0000(	OPRL1/NA	GO_BP
GO:009000	2	2/1117	12/20610	0.135219	0.467407	3.075201	ENSG0000(	ETS2/SRF	GO_BP
GO:000695	8	8/1117	94/20610	0.137123	0.471314	1.570316	ENSG0000(	CFD/NA/F	GO_BP
GO:009950	3	3/1117	24/20610	0.138323	0.471314	2.306401	ENSG0000(	STX4/VAM	GO_BP
GO:000188	4	4/1117	37/20610	0.138531	0.471314	1.994725	ENSG0000(	RAB11B/S	GO_BP
GO:009750	4	4/1117	37/20610	0.138531	0.471314	1.994725	ENSG0000(	SDF2/DPM	GO_BP
GO:000150	27	27/1117	399/2061(	0.139001	0.472604	1.248578	ENSG0000(	KLF2/RGS	GO_BP
GO:002153	20	20/1117	284/2061(	0.139984	0.475635	1.299381	ENSG0000(	KDM6B/RH	GO_BP
GO:190382	24	24/1117	350/2061(	0.141045	0.478007	1.265226	ENSG0000(	UBL5/ABH	GO_BP
GO:004814	5	5/1117	51/20610	0.141143	0.478007	1.808942	ENSG0000(	S100A6/C	GO_BP
GO:005170	8	8/1117	95/20610	0.143099	0.483053	1.553786	ENSG0000(	CALM3/GP	GO_BP
GO:190022	3	3/1117	25/20610	0.151219	0.501837	2.214145	ENSG0000(	SRF/HMGC	GO_BP
GO:004575	2	2/1117	13/20610	0.154316	0.501837	2.838647	ENSG0000(	KCNE3/BI	GO_BP
GO:000070	6	6/1117	67/20610	0.154471	0.501837	1.652347	ENSG0000(	TYROBP/P	GO_BP
GO:004885	6	6/1117	67/20610	0.154471	0.501837	1.652347	ENSG0000(	CALM3/CO	GO_BP
GO:005190	23	23/1117	338/2061(	0.155325	0.503687	1.255556	ENSG0000(	VIM/DYNL	GO_BP
GO:003241	8	8/1117	97/20610	0.155429	0.503687	1.521749	ENSG0000(	MMP9/CAL	GO_BP
GO:003090	10	10/1117	128/2061(	0.156743	0.507312	1.441501	ENSG0000(	CTDSP2/H	GO_BP
GO:004390	32	32/1117	490/2061(	0.15875	0.510364	1.204977	ENSG0000(	IFITM2/B	GO_BP
GO:003262	4	4/1117	39/20610	0.158966	0.510364	1.892432	ENSG0000(	MYD88/IL	GO_BP
GO:000758	17	17/1117	241/2061(	0.161644	0.518321	1.301538	ENSG0000(	AQP3/CDK	GO_BP
GO:004591	7	7/1117	83/20610	0.162888	0.519694	1.556126	ENSG0000(	IRS2/WDR	GO_BP
GO:000191	3	3/1117	26/20610	0.164478	0.519694	2.128986	ENSG0000(	SNCA/CHM	GO_BP
GO:003022	3	3/1117	26/20610	0.164478	0.519694	2.128986	ENSG0000(	SRF/NBEA	GO_BP
GO:190040	3	3/1117	26/20610	0.164478	0.519694	2.128986	ENSG0000(	MCL1/TLR	GO_BP
GO:200001	3	3/1117	26/20610	0.164478	0.519694	2.128986	ENSG0000(	TYROBP/S	GO_BP
GO:200017	3	3/1117	26/20610	0.164478	0.519694	2.128986	ENSG0000(	KCTD11/N	GO_BP
GO:001052	5	5/1117	54/20610	0.167315	0.527372	1.708445	ENSG0000(	CALM3/SR	GO_BP
GO:004852	11	11/1117	146/2061(	0.169089	0.530947	1.39016	ENSG0000(	IFITM2/B	GO_BP
GO:000694	6	6/1117	69/20610	0.170101	0.531631	1.604453	ENSG0000(	TYROBP/P	GO_BP
GO:004420	20	20/1117	293/2061(	0.171881	0.532856	1.259468	ENSG0000(	NAGK/IRS	GO_BP

GO:00102	2	2/1117	14/20610	0.173885	0.532856	2.635887	ENSG0000	MT2A/MT1	GO_BP
GO:00906	2	2/1117	14/20610	0.173885	0.532856	2.635887	ENSG0000	SOD2/NA	GO_BP
GO:00907	2	2/1117	14/20610	0.173885	0.532856	2.635887	ENSG0000	CD81/IL2	GO_BP
GO:19017	2	2/1117	14/20610	0.173885	0.532856	2.635887	ENSG0000	FLCN/MYC	GO_BP
GO:00009	13	13/1117	179/2061	0.174333	0.533283	1.340032	ENSG0000	CALM3/RH	GO_BP
GO:19037	13	13/1117	179/2061	0.174333	0.533283	1.340032	ENSG0000	NFKBIA/Z	GO_BP
GO:00485	9	9/1117	116/2061	0.177725	0.538932	1.431559	ENSG0000	POLR2C/I	GO_BP
GO:00095	3	3/1117	27/20610	0.178064	0.538932	2.050134	ENSG0000	TLR2/TLR	GO_BP
GO:00605	3	3/1117	27/20610	0.178064	0.538932	2.050134	ENSG0000	BCL2L11/	GO_BP
GO:00711	11	11/1117	148/2061	0.179904	0.543579	1.371374	ENSG0000	SRSF1/U2	GO_BP
GO:00481	15	15/1117	213/2061	0.181532	0.546598	1.299381	ENSG0000	TYROBP/D	GO_BP
GO:00508	7	7/1117	86/20610	0.18459	0.554842	1.501843	ENSG0000	DGKI/EIF	GO_BP
GO:00107	3	3/1117	28/20610	0.191944	0.56331	1.976915	ENSG0000	VIM/ARRB	GO_BP
GO:00322	3	3/1117	28/20610	0.191944	0.56331	1.976915	ENSG0000	POLE4/UP	GO_BP
GO:19028	3	3/1117	28/20610	0.191944	0.56331	1.976915	ENSG0000	MCL1/TLR	GO_BP
GO:00024	2	2/1117	15/20610	0.193818	0.56331	2.460161	ENSG0000	BCL3/TNF	GO_BP
GO:00602	2	2/1117	15/20610	0.193818	0.56331	2.460161	ENSG0000	TSPO/HES	GO_BP
GO:19010	2	2/1117	15/20610	0.193818	0.56331	2.460161	ENSG0000	SLC25A5/	GO_BP
GO:19035	2	2/1117	15/20610	0.193818	0.56331	2.460161	ENSG0000	SDCBP/CH	GO_BP
GO:00989	5	5/1117	57/20610	0.195219	0.566177	1.618527	ENSG0000	KCNE3/BI	GO_BP
GO:00083	12	12/1117	168/2061	0.201185	0.581786	1.317943	ENSG0000	CDC42SE1	GO_BP
GO:00105	8	8/1117	104/2061	0.202256	0.583133	1.419324	ENSG0000	CALM3/SR	GO_BP
GO:19058	4	4/1117	43/20610	0.202775	0.583133	1.716391	ENSG0000	PPP1R15A	GO_BP
GO:00093	31	31/1117	488/2061	0.203339	0.584107	1.172105	ENSG0000	CARD16/C	GO_BP
GO:00067	3	3/1117	29/20610	0.206081	0.589698	1.908746	ENSG0000	PFKM/ALD	GO_BP
GO:00363	3	3/1117	29/20610	0.206081	0.589698	1.908746	ENSG0000	CALR/CCR	GO_BP
GO:00457	31	31/1117	489/2061	0.206542	0.590689	1.169709	ENSG0000	AIF1/NA/	GO_BP
GO:00353	9	9/1117	121/2061	0.209881	0.598258	1.372404	ENSG0000	PDHB/SNC	GO_BP
GO:00075	12	12/1117	170/2061	0.212267	0.598519	1.302438	ENSG0000	B2M/NA/P	GO_BP
GO:00069	2	2/1117	16/20610	0.214018	0.598519	2.306401	ENSG0000	FCN1/MFG	GO_BP
GO:00309	2	2/1117	16/20610	0.214018	0.598519	2.306401	ENSG0000	RHOB/RHO	GO_BP
GO:00400	2	2/1117	16/20610	0.214018	0.598519	2.306401	ENSG0000	HMGN1/HE	GO_BP
GO:00514	2	2/1117	16/20610	0.214018	0.598519	2.306401	ENSG0000	DUSP1/PR	GO_BP
GO:00906	2	2/1117	16/20610	0.214018	0.598519	2.306401	ENSG0000	B2M/NA	GO_BP
GO:00975	2	2/1117	16/20610	0.214018	0.598519	2.306401	ENSG0000	MT2A/MT1	GO_BP
GO:19002	2	2/1117	16/20610	0.214018	0.598519	2.306401	ENSG0000	SOD2/NA	GO_BP
GO:19035	2	2/1117	16/20610	0.214018	0.598519	2.306401	ENSG0000	SDCBP/CH	GO_BP
GO:19021	5	5/1117	59/20610	0.214656	0.598705	1.563662	ENSG0000	CHMP2A/T	GO_BP
GO:00466	9	9/1117	122/2061	0.216578	0.601482	1.361155	ENSG0000	DUSP6/RG	GO_BP
GO:00020	8	8/1117	106/2061	0.21658	0.601482	1.392544	ENSG0000	CALM3/SR	GO_BP
GO:00458	11	11/1117	155/2061	0.220154	0.607658	1.309441	ENSG0000	IRS2/CTD	GO_BP
GO:19038	3	3/1117	30/20610	0.220445	0.607658	1.845121	ENSG0000	SDCBP/FL	GO_BP
GO:00605	17	17/1117	255/2061	0.222344	0.612242	1.230081	ENSG0000	TWF2/DBN	GO_BP
GO:19035	4	4/1117	45/20610	0.225885	0.617403	1.640107	ENSG0000	SRI/ZC3H	GO_BP
GO:00508	14	14/1117	206/2061	0.2283	0.62025	1.253966	ENSG0000	TYROBP/A	GO_BP
GO:00512	10	10/1117	140/2061	0.228443	0.62025	1.317943	ENSG0000	CALM3/SR	GO_BP
GO:00094	21	21/1117	325/2061	0.23254	0.62025	1.192232	ENSG0000	RGS2/MGS	GO_BP
GO:00019	2	2/1117	17/20610	0.234399	0.62025	2.17073	ENSG0000	SNCA/HGN	GO_BP
GO:00336	2	2/1117	17/20610	0.234399	0.62025	2.17073	ENSG0000	ATRAID/T	GO_BP
GO:00349	2	2/1117	17/20610	0.234399	0.62025	2.17073	ENSG0000	CALR/DNA	GO_BP
GO:00454	2	2/1117	17/20610	0.234399	0.62025	2.17073	ENSG0000	OPRL1/NA	GO_BP
GO:00465	2	2/1117	17/20610	0.234399	0.62025	2.17073	ENSG0000	AHCYL1/M	GO_BP
GO:20001	2	2/1117	17/20610	0.234399	0.62025	2.17073	ENSG0000	PIM1/HES	GO_BP
GO:00506	7	7/1117	93/20610	0.239357	0.628388	1.388801	ENSG0000	DHX58/IF	GO_BP

GO:00485	17	17/1117	259/2061	0.241328	0.632922	1.211083	ENSG0000	TWF2/RGS	GO_BP
GO:00328	16	16/1117	243/2061	0.245877	0.643226	1.214894	ENSG0000	CHMP2A/M	GO_BP
GO:20001	24	24/1117	380/2061	0.247364	0.6457	1.165339	ENSG0000	BST2/AIF	GO_BP
GO:00199	6	6/1117	78/20610	0.247446	0.6457	1.419324	ENSG0000	NFKBIA/Z	GO_BP
GO:19001	6	6/1117	78/20610	0.247446	0.6457	1.419324	ENSG0000	PRKCD/ZC	GO_BP
GO:00324	19	19/1117	295/2061	0.250684	0.650222	1.188383	ENSG0000	TMSB4X/M	GO_BP
GO:00067	25	25/1117	399/2061	0.254349	0.651127	1.156091	ENSG0000	MGST3/IS	GO_BP
GO:00358	13	13/1117	194/2061	0.254373	0.651127	1.236421	ENSG0000	LYZ/CALM	GO_BP
GO:00400	28	28/1117	451/2061	0.25447	0.651127	1.14553	ENSG0000	BST2/AIF	GO_BP
GO:00517	2	2/1117	18/20610	0.254881	0.651127	2.050134	ENSG0000	TXNIP/MY	GO_BP
GO:00901	2	2/1117	18/20610	0.254881	0.651127	2.050134	ENSG0000	MMP9/FLC	GO_BP
GO:00323	5	5/1117	63/20610	0.255176	0.651127	1.464382	ENSG0000	NFKBIA/P	GO_BP
GO:00521	5	5/1117	63/20610	0.255176	0.651127	1.464382	ENSG0000	CHMP2A/T	GO_BP
GO:00098	22	22/1117	349/2061	0.261824	0.664493	1.163113	ENSG0000	SDCBP/MC	GO_BP
GO:00606	3	3/1117	33/20610	0.264567	0.667859	1.677383	ENSG0000	JUNB/HES	GO_BP
GO:00025	5	5/1117	64/20610	0.26559	0.669205	1.441501	ENSG0000	ADCY7/ZC	GO_BP
GO:00108	15	15/1117	230/2061	0.266505	0.67014	1.20334	ENSG0000	NINJ1/RA	GO_BP
GO:00315	24	24/1117	385/2061	0.267817	0.673112	1.150205	ENSG0000	NINJ1/RA	GO_BP
GO:00434	13	13/1117	197/2061	0.271821	0.680608	1.217592	ENSG0000	TMSB4X/P	GO_BP
GO:00069	4	4/1117	49/20610	0.273852	0.680608	1.506221	ENSG0000	PPP1R15A	GO_BP
GO:00073	2	2/1117	19/20610	0.275394	0.680608	1.942232	ENSG0000	CD9/FOLR	GO_BP
GO:00160	2	2/1117	19/20610	0.275394	0.680608	1.942232	ENSG0000	SNCA/VAM	GO_BP
GO:19059	2	2/1117	19/20610	0.275394	0.680608	1.942232	ENSG0000	RETN/CIT	GO_BP
GO:20006	2	2/1117	19/20610	0.275394	0.680608	1.942232	ENSG0000	KCTD11/N	GO_BP
GO:00322	5	5/1117	65/20610	0.276096	0.681695	1.419324	ENSG0000	PFN1/GPR	GO_BP
GO:19018	5	5/1117	66/20610	0.286686	0.697871	1.397819	ENSG0000	RGS2/FLC	GO_BP
GO:19037	5	5/1117	66/20610	0.286686	0.697871	1.397819	ENSG0000	UBL5/RAC	GO_BP
GO:00324	8	8/1117	116/2061	0.293121	0.705335	1.272497	ENSG0000	TMSB4X/C	GO_BP
GO:00099	6	6/1117	83/20610	0.294122	0.705335	1.333822	ENSG0000	CD9/LGAL	GO_BP
GO:00513	3	3/1117	35/20610	0.294544	0.705335	1.581532	ENSG0000	SNCA/HP/	GO_BP
GO:00512	14	14/1117	218/2061	0.294886	0.705335	1.18494	ENSG0000	SRSF1/U2	GO_BP
GO:00011	23	23/1117	374/2061	0.295204	0.705335	1.1347	ENSG0000	KLF2/AQP	GO_BP
GO:00456	7	7/1117	100/2061	0.29855	0.708783	1.291585	ENSG0000	AQP3/MAF	GO_BP
GO:00313	21	21/1117	342/2061	0.308581	0.727985	1.132969	ENSG0000	ADCY7/DH	GO_BP
GO:00512	9	9/1117	135/2061	0.310033	0.727985	1.230081	ENSG0000	CALM3/SR	GO_BP
GO:00901	4	4/1117	52/20610	0.31085	0.727985	1.419324	ENSG0000	PSAP/DVL	GO_BP
GO:19059	6	6/1117	85/20610	0.313274	0.729136	1.302438	ENSG0000	NFKBIA/P	GO_BP
GO:19015	13	13/1117	204/2061	0.313945	0.729136	1.175812	ENSG0000	MGST3/AL	GO_BP
GO:00307	2	2/1117	21/20610	0.316263	0.729136	1.757258	ENSG0000	RGS2/PTG	GO_BP
GO:00328	2	2/1117	21/20610	0.316263	0.729136	1.757258	ENSG0000	RGS2/IRS	GO_BP
GO:00301	4	4/1117	53/20610	0.323296	0.740015	1.392544	ENSG0000	PRKCD/SE	GO_BP
GO:00305	4	4/1117	53/20610	0.323296	0.740015	1.392544	ENSG0000	MMP9/MMP	GO_BP
GO:00326	10	10/1117	154/2061	0.324411	0.740015	1.19813	ENSG0000	FADD/ISG	GO_BP
GO:20010	7	7/1117	103/2061	0.324836	0.740015	1.253966	ENSG0000	RGS2/PSA	GO_BP
GO:00512	9	9/1117	137/2061	0.325208	0.740211	1.212123	ENSG0000	CALM3/SR	GO_BP
GO:00322	7	7/1117	104/2061	0.333682	0.754355	1.241908	ENSG0000	PFN1/GPR	GO_BP
GO:19000	4	4/1117	54/20610	0.335775	0.754355	1.366756	ENSG0000	PRKCD/SE	GO_BP
GO:00450	2	2/1117	22/20610	0.336514	0.754355	1.677383	ENSG0000	SRF/CD74	GO_BP
GO:00613	2	2/1117	22/20610	0.336514	0.754355	1.677383	ENSG0000	RHOA/MSN	GO_BP
GO:01401	2	2/1117	22/20610	0.336514	0.754355	1.677383	ENSG0000	SDCBP/CH	GO_BP
GO:00344	3	3/1117	38/20610	0.339808	0.759426	1.456674	ENSG0000	KLF2/PTG	GO_BP
GO:19000	3	3/1117	38/20610	0.339808	0.759426	1.456674	ENSG0000	PRKCD/CI	GO_BP
GO:19010	3	3/1117	38/20610	0.339808	0.759426	1.456674	ENSG0000	BBC3/BAK	GO_BP
GO:00426	5	5/1117	71/20610	0.340533	0.759426	1.299381	ENSG0000	ARRB2/RE	GO_BP

GO:00513	8	8/1117	122/2061	0.341836	0.761676	1.209915	ENSG0000	CALM3/GN	GO_BP
GO:19021	14	14/1117	226/2061	0.342147	0.762042	1.142995	ENSG0000	SDCBP/CH	GO_BP
GO:00017	9	9/1117	140/2061	0.348221	0.772365	1.186149	ENSG0000	MMP9/KDM	GO_BP
GO:00220	8	8/1117	123/2061	0.350078	0.773583	1.200079	ENSG0000	AIF1/NA/	GO_BP
GO:00302	8	8/1117	123/2061	0.350078	0.773583	1.200079	ENSG0000	ATRAID/S	GO_BP
GO:00427	3	3/1117	39/20610	0.354878	0.773583	1.419324	ENSG0000	UBC/DDB1	GO_BP
GO:00515	3	3/1117	39/20610	0.354878	0.773583	1.419324	ENSG0000	ARL6IP1/	GO_BP
GO:00716	3	3/1117	39/20610	0.354878	0.773583	1.419324	ENSG0000	TYROBP/F	GO_BP
GO:00072	2	2/1117	23/20610	0.356579	0.773583	1.604453	ENSG0000	HMGB2/PY	GO_BP
GO:00901	2	2/1117	23/20610	0.356579	0.773583	1.604453	ENSG0000	PRKCD/SM	GO_BP
GO:20010	2	2/1117	23/20610	0.356579	0.773583	1.604453	ENSG0000	PSAP/TXN	GO_BP
GO:00970	4	4/1117	56/20610	0.360774	0.779563	1.317943	ENSG0000	PRKCD/SL	GO_BP
GO:19900	4	4/1117	56/20610	0.360774	0.779563	1.317943	ENSG0000	EIF4A3/A	GO_BP
GO:00016	12	12/1117	194/2061	0.36099	0.779563	1.141312	ENSG0000	KDM6B/FL	GO_BP
GO:00487	12	12/1117	194/2061	0.36099	0.779563	1.141312	ENSG0000	PBX2/NA/	GO_BP
GO:00071	20	20/1117	336/2061	0.365158	0.78758	1.098286	ENSG0000	DUSP1/ZN	GO_BP
GO:00018	15	15/1117	248/2061	0.368439	0.792746	1.116001	ENSG0000	LYZ/B2M/	GO_BP
GO:19027	3	3/1117	40/20610	0.369902	0.792746	1.383841	ENSG0000	BCL2L11/	GO_BP
GO:20001	6	6/1117	91/20610	0.371664	0.792746	1.216563	ENSG0000	RHOA/GNA	GO_BP
GO:19000	5	5/1117	74/20610	0.373222	0.792746	1.246703	ENSG0000	PRKCD/GN	GO_BP
GO:19037	5	5/1117	74/20610	0.373222	0.792746	1.246703	ENSG0000	CALM3/SR	GO_BP
GO:00508	4	4/1117	57/20610	0.373269	0.792746	1.294822	ENSG0000	PRKCD/SE	GO_BP
GO:00611	4	4/1117	57/20610	0.373269	0.792746	1.294822	ENSG0000	PIM3/SRI	GO_BP
GO:00351	10	10/1117	161/2061	0.375278	0.792746	1.146038	ENSG0000	PBX2/NA/	GO_BP
GO:19000	2	2/1117	24/20610	0.376421	0.792746	1.537601	ENSG0000	GNAI2/AD	GO_BP
GO:00458	5	5/1117	75/20610	0.384127	0.804215	1.230081	ENSG0000	SDCBP/TL	GO_BP
GO:00971	4	4/1117	58/20610	0.385744	0.804215	1.272497	ENSG0000	TYROBP/D	GO_BP
GO:00300	9	9/1117	146/2061	0.394824	0.815318	1.137403	ENSG0000	VIM/SRI/	GO_BP
GO:00020	2	2/1117	25/20610	0.396003	0.815318	1.476097	ENSG0000	IRS2/MYC	GO_BP
GO:00148	2	2/1117	25/20610	0.396003	0.815318	1.476097	ENSG0000	METRNL/N	GO_BP
GO:00092	3	3/1117	42/20610	0.399735	0.817928	1.317943	ENSG0000	RAB11B/G	GO_BP
GO:00466	3	3/1117	42/20610	0.399735	0.817928	1.317943	ENSG0000	PPIF/ZC3	GO_BP
GO:00215	7	7/1117	113/2061	0.414202	0.826613	1.142995	ENSG0000	AIF1/NA/	GO_BP
GO:00070	12	12/1117	202/2061	0.414229	0.826613	1.096111	ENSG0000	CHMP2A/M	GO_BP
GO:00903	3	3/1117	43/20610	0.414508	0.826613	1.287294	ENSG0000	TXN/BAG3	GO_BP
GO:00481	2	2/1117	26/20610	0.415297	0.826613	1.419324	ENSG0000	FADD/SNC	GO_BP
GO:00487	2	2/1117	26/20610	0.415297	0.826613	1.419324	ENSG0000	BLOC1S4/	GO_BP
GO:00515	2	2/1117	26/20610	0.415297	0.826613	1.419324	ENSG0000	RGS2/SNC	GO_BP
GO:00518	2	2/1117	26/20610	0.415297	0.826613	1.419324	ENSG0000	RAB11B/B	GO_BP
GO:00309	10	10/1117	167/2061	0.419451	0.826613	1.104863	ENSG0000	AIF1/NA/	GO_BP
GO:00064	15	15/1117	257/2061	0.42167	0.826613	1.076919	ENSG0000	SRSF1/NO	GO_BP
GO:00430	27	27/1117	474/2061	0.422838	0.826613	1.051018	ENSG0000	SDCBP/MM	GO_BP
GO:00031	1	1/1117	10/20610	0.427267	0.826613	1.845121	ENSG0000	FZD2	GO_BP
GO:00309	1	1/1117	10/20610	0.427267	0.826613	1.845121	ENSG0000	HES1	GO_BP
GO:00326	1	1/1117	10/20610	0.427267	0.826613	1.845121	ENSG0000	TLR2	GO_BP
GO:00336	1	1/1117	10/20610	0.427267	0.826613	1.845121	ENSG0000	GATA1	GO_BP
GO:00448	1	1/1117	10/20610	0.427267	0.826613	1.845121	ENSG0000	HYAL2	GO_BP
GO:00487	1	1/1117	10/20610	0.427267	0.826613	1.845121	ENSG0000	CD63	GO_BP
GO:00514	1	1/1117	10/20610	0.427267	0.826613	1.845121	ENSG0000	DUSP6	GO_BP
GO:00519	1	1/1117	10/20610	0.427267	0.826613	1.845121	ENSG0000	TSPO	GO_BP
GO:00600	1	1/1117	10/20610	0.427267	0.826613	1.845121	ENSG0000	NINJ1	GO_BP
GO:00720	1	1/1117	10/20610	0.427267	0.826613	1.845121	ENSG0000	HES1	GO_BP
GO:00722	1	1/1117	10/20610	0.427267	0.826613	1.845121	ENSG0000	MYC	GO_BP
GO:00907	1	1/1117	10/20610	0.427267	0.826613	1.845121	ENSG0000	IL23A	GO_BP

GO:19024	1	1/1117	10/20610	0.427267	0.826613	1.845121	ENSG0000	ZNF706	GO_BP
GO:19037	1	1/1117	10/20610	0.427267	0.826613	1.845121	ENSG0000	BAG3	GO_BP
GO:20002	1	1/1117	10/20610	0.427267	0.826613	1.845121	ENSG0000	CFAP20	GO_BP
GO:20008	1	1/1117	10/20610	0.427267	0.826613	1.845121	ENSG0000	RETN	GO_BP
GO:00031	5	5/1117	79/20610	0.427575	0.826613	1.167798	ENSG0000	PLXND1/D	GO_BP
GO:00301	5	5/1117	79/20610	0.427575	0.826613	1.167798	ENSG0000	PRKCD/SE	GO_BP
GO:00422	5	5/1117	79/20610	0.427575	0.826613	1.167798	ENSG0000	GPX1/NIN	GO_BP
GO:19030	6	6/1117	97/20610	0.430366	0.830151	1.141312	ENSG0000	PRKCD/SE	GO_BP
GO:00023	2	2/1117	27/20610	0.434276	0.832069	1.366756	ENSG0000	TXNIP/CD	GO_BP
GO:00459	2	2/1117	27/20610	0.434276	0.832069	1.366756	ENSG0000	PRKACA/N	GO_BP
GO:00996	2	2/1117	27/20610	0.434276	0.832069	1.366756	ENSG0000	STX3/HGN	GO_BP
GO:00024	5	5/1117	80/20610	0.438355	0.835921	1.153201	ENSG0000	ARPC3/PR	GO_BP
GO:00083	5	5/1117	80/20610	0.438355	0.835921	1.153201	ENSG0000	CSTB/ARR	GO_BP
GO:19000	5	5/1117	80/20610	0.438355	0.835921	1.153201	ENSG0000	PRKCD/SE	GO_BP
GO:00613	9	9/1117	152/2061	0.441625	0.838828	1.092506	ENSG0000	CALM3/SR	GO_BP
GO:00020	2	2/1117	28/20610	0.452917	0.838828	1.317943	ENSG0000	GAA/MYL4	GO_BP
GO:00360	2	2/1117	28/20610	0.452917	0.838828	1.317943	ENSG0000	INPPL1/M	GO_BP
GO:00082	11	11/1117	190/2061	0.455277	0.838828	1.068228	ENSG0000	SOD2/NA/	GO_BP
GO:19059	10	10/1117	172/2061	0.456231	0.838828	1.072745	ENSG0000	HNRNPK/N	GO_BP
GO:00341	3	3/1117	46/20610	0.458053	0.838828	1.20334	ENSG0000	ADAM8/IL	GO_BP
GO:00456	3	3/1117	46/20610	0.458053	0.838828	1.20334	ENSG0000	TRADD/KR	GO_BP
GO:00215	1	1/1117	11/20610	0.458322	0.838828	1.677383	ENSG0000	ATF5	GO_BP
GO:00510	1	1/1117	11/20610	0.458322	0.838828	1.677383	ENSG0000	GMFG	GO_BP
GO:00702	1	1/1117	11/20610	0.458322	0.838828	1.677383	ENSG0000	RGCC	GO_BP
GO:19012	1	1/1117	11/20610	0.458322	0.838828	1.677383	ENSG0000	RGCC	GO_BP
GO:00486	4	4/1117	64/20610	0.459539	0.839101	1.153201	ENSG0000	RGS2/YBX	GO_BP
GO:00350	5	5/1117	82/20610	0.459766	0.839101	1.125074	ENSG0000	POLR2C/Z	GO_BP
GO:00901	21	21/1117	373/2061	0.460692	0.840496	1.038808	ENSG0000	TMSB4X/A	GO_BP
GO:00347	12	12/1117	210/2061	0.467614	0.84846	1.054355	ENSG0000	TMSB4X/I	GO_BP
GO:00351	2	2/1117	29/20610	0.471201	0.84846	1.272497	ENSG0000	NDUFA2/U	GO_BP
GO:20010	2	2/1117	29/20610	0.471201	0.84846	1.272497	ENSG0000	ARL6IP1/	GO_BP
GO:00075	4	4/1117	65/20610	0.471568	0.84846	1.135459	ENSG0000	PSAP/SLC	GO_BP
GO:01061	9	9/1117	156/2061	0.472614	0.84846	1.064493	ENSG0000	KDM6B/FL	GO_BP
GO:01201	9	9/1117	156/2061	0.472614	0.84846	1.064493	ENSG0000	KDM6B/FL	GO_BP
GO:00611	12	12/1117	211/2061	0.474249	0.84846	1.049358	ENSG0000	PLXND1/S	GO_BP
GO:00435	7	7/1117	120/2061	0.476463	0.84846	1.076321	ENSG0000	RGS2/AIF	GO_BP
GO:00351	9	9/1117	157/2061	0.480309	0.84846	1.057713	ENSG0000	PFN1/BCL	GO_BP
GO:00508	5	5/1117	84/20610	0.480936	0.84846	1.098286	ENSG0000	PRKCD/SE	GO_BP
GO:00991	27	27/1117	488/2061	0.48434	0.84846	1.020866	ENSG0000	CALM3/TY	GO_BP
GO:01201	3	3/1117	48/20610	0.486303	0.84846	1.153201	ENSG0000	FLCN/TLR	GO_BP
GO:00024	1	1/1117	12/20610	0.487695	0.84846	1.537601	ENSG0000	CD274	GO_BP
GO:00069	1	1/1117	12/20610	0.487695	0.84846	1.537601	ENSG0000	SCAP	GO_BP
GO:00434	1	1/1117	12/20610	0.487695	0.84846	1.537601	ENSG0000	CD63	GO_BP
GO:00434	1	1/1117	12/20610	0.487695	0.84846	1.537601	ENSG0000	CD63	GO_BP
GO:00446	1	1/1117	12/20610	0.487695	0.84846	1.537601	ENSG0000	CD81	GO_BP
GO:00606	1	1/1117	12/20610	0.487695	0.84846	1.537601	ENSG0000	SOCS3	GO_BP
GO:00607	1	1/1117	12/20610	0.487695	0.84846	1.537601	ENSG0000	PSAP	GO_BP
GO:00607	1	1/1117	12/20610	0.487695	0.84846	1.537601	ENSG0000	PSAP	GO_BP
GO:00614	1	1/1117	12/20610	0.487695	0.84846	1.537601	ENSG0000	CALR	GO_BP
GO:00725	1	1/1117	12/20610	0.487695	0.84846	1.537601	ENSG0000	RGCC	GO_BP
GO:00905	1	1/1117	12/20610	0.487695	0.84846	1.537601	ENSG0000	S1PR1	GO_BP
GO:19011	1	1/1117	12/20610	0.487695	0.84846	1.537601	ENSG0000	CALR	GO_BP
GO:19041	1	1/1117	12/20610	0.487695	0.84846	1.537601	ENSG0000	TSPO	GO_BP
GO:00068	2	2/1117	30/20610	0.489112	0.84846	1.230081	ENSG0000	CLNS1A/E	GO_BP

GO:00106:	10	10/1117	177/2061(	0.492658	0.851761	1.042441	ENSG0000(	TMSB4X/A/	GO_BP
GO:00459	3	3/1117	49/20610	0.500158	0.858576	1.129666	ENSG0000(	DDIT4/MII	GO_BP
GO:00352(	12	12/1117	215/2061(	0.500632	0.858576	1.029835	ENSG0000(	DUSP6/RG:	GO_BP
GO:00102!	26	26/1117	474/2061(	0.503842	0.858576	1.012092	ENSG0000(	PSAP/RILI	GO_BP
GO:00519{	2	2/1117	31/20610	0.506637	0.858576	1.190401	ENSG0000(	RCC2/NA	GO_BP
GO:00032!	3	3/1117	50/20610	0.513821	0.858576	1.107073	ENSG0000(	IFI6/TSP(	GO_BP
GO:00107	3	3/1117	50/20610	0.513821	0.858576	1.107073	ENSG0000(	VIM/ARRB:	GO_BP
GO:00215	3	3/1117	50/20610	0.513821	0.858576	1.107073	ENSG0000(	ABT1/NFE:	GO_BP
GO:00603:	3	3/1117	50/20610	0.513821	0.858576	1.107073	ENSG0000(	SRF/RRAS,	GO_BP
GO:00613{	3	3/1117	50/20610	0.513821	0.858576	1.107073	ENSG0000(	RHOA/SRF,	GO_BP
GO:00701(	3	3/1117	50/20610	0.513821	0.858576	1.107073	ENSG0000(	ATRAID/I:	GO_BP
GO:00019(	1	1/1117	13/20610	0.515477	0.858576	1.419324	ENSG0000(	UBE2Q1	GO_BP
GO:00975:	1	1/1117	13/20610	0.515477	0.858576	1.419324	ENSG0000(	FADD	GO_BP
GO:00989(	1	1/1117	13/20610	0.515477	0.858576	1.419324	ENSG0000(	KCNE3	GO_BP
GO:00706(	6	6/1117	106/2061(	0.516296	0.859664	1.044408	ENSG0000(	TYROBP/MI	GO_BP
GO:00060(	22	22/1117	403/2061(	0.51756	0.861492	1.007262	ENSG0000(	CEBPA/DHI	GO_BP
GO:00326:	4	4/1117	69/20610	0.518597	0.862664	1.069635	ENSG0000(	PNP/IRF4,	GO_BP
GO:20007'	4	4/1117	69/20610	0.518597	0.862664	1.069635	ENSG0000(	B2M/NA/HI	GO_BP
GO:00217'	2	2/1117	32/20610	0.523765	0.864038	1.153201	ENSG0000(	SRF/ATF5	GO_BP
GO:00482	2	2/1117	32/20610	0.523765	0.864038	1.153201	ENSG0000(	PRKACA/N/	GO_BP
GO:00519!	2	2/1117	32/20610	0.523765	0.864038	1.153201	ENSG0000(	RGS2/SNC/	GO_BP
GO:00602!	2	2/1117	32/20610	0.523765	0.864038	1.153201	ENSG0000(	RETN/CFAI	GO_BP
GO:00906!	2	2/1117	32/20610	0.523765	0.864038	1.153201	ENSG0000(	PSAP/ARRI	GO_BP
GO:00309(	23	23/1117	423/2061(	0.52446	0.864038	1.003257	ENSG0000(	B2M/NA/T'	GO_BP
GO:00107	3	3/1117	51/20610	0.527281	0.864038	1.085365	ENSG0000(	SDCBP/ZNI	GO_BP
GO:00316	4	4/1117	70/20610	0.530046	0.864038	1.054355	ENSG0000(	OPRL1/NA,	GO_BP
GO:00466:	4	4/1117	70/20610	0.530046	0.864038	1.054355	ENSG0000(	PIM1/HLX,	GO_BP
GO:00486:	20	20/1117	370/2061(	0.537871	0.864038	0.997363	ENSG0000(	DUSP6/TWI	GO_BP
GO:00104(	2	2/1117	33/20610	0.540487	0.864038	1.118255	ENSG0000(	IRS2/MYC	GO_BP
GO:00357'	2	2/1117	33/20610	0.540487	0.864038	1.118255	ENSG0000(	SRI/STX4	GO_BP
GO:20010:	2	2/1117	33/20610	0.540487	0.864038	1.118255	ENSG0000(	PSAP/TXN	GO_BP
GO:00514	3	3/1117	52/20610	0.540532	0.864038	1.064493	ENSG0000(	DUSP1/CAI	GO_BP
GO:00196:	1	1/1117	14/20610	0.541753	0.864038	1.317943	ENSG0000(	CEBPA	GO_BP
GO:00358	1	1/1117	14/20610	0.541753	0.864038	1.317943	ENSG0000(	GNAI2	GO_BP
GO:00433{	1	1/1117	14/20610	0.541753	0.864038	1.317943	ENSG0000(	CD74	GO_BP
GO:00517	1	1/1117	14/20610	0.541753	0.864038	1.317943	ENSG0000(	BCL2L11	GO_BP
GO:00610:	1	1/1117	14/20610	0.541753	0.864038	1.317943	ENSG0000(	SRF	GO_BP
GO:00713!	1	1/1117	14/20610	0.541753	0.864038	1.317943	ENSG0000(	WBP2	GO_BP
GO:01201(	6	6/1117	109/2061(	0.543858	0.865798	1.015663	ENSG0000(	KDM6B/FH,	GO_BP
GO:19018{	6	6/1117	109/2061(	0.543858	0.865798	1.015663	ENSG0000(	S100A10/I	GO_BP
GO:00702(	7	7/1117	128/2061(	0.545255	0.867755	1.00905	ENSG0000(	FURIN/CS'	GO_BP
GO:00018!	5	5/1117	91/20610	0.552466	0.875141	1.013803	ENSG0000(	JUNB/CEBI	GO_BP
GO:00512!	4	4/1117	72/20610	0.552534	0.875141	1.025067	ENSG0000(	CHMP2A/CI	GO_BP
GO:00720!	4	4/1117	72/20610	0.552534	0.875141	1.025067	ENSG0000(	PIM1/HMGI	GO_BP
GO:00903	4	4/1117	72/20610	0.552534	0.875141	1.025067	ENSG0000(	B2M/NA/HI	GO_BP
GO:00029:	3	3/1117	53/20610	0.553566	0.875141	1.044408	ENSG0000(	PANX2/PP	GO_BP
GO:00086(	2	2/1117	34/20610	0.556798	0.875141	1.085365	ENSG0000(	RCC2/NA	GO_BP
GO:00109{	2	2/1117	34/20610	0.556798	0.875141	1.085365	ENSG0000(	HNRNPK/LI	GO_BP
GO:00603:	2	2/1117	34/20610	0.556798	0.875141	1.085365	ENSG0000(	RRAS/CSRI	GO_BP
GO:00320{	6	6/1117	111/2061(	0.561842	0.875141	0.997363	ENSG0000(	TMSB4X/NI	GO_BP
GO:00017(	12	12/1117	225/2061(	0.564918	0.875141	0.984064	ENSG0000(	PLXND1/SI	GO_BP
GO:00329(	3	3/1117	54/20610	0.566378	0.875141	1.025067	ENSG0000(	VIM/ARRB:	GO_BP
GO:00451(	3	3/1117	54/20610	0.566378	0.875141	1.025067	ENSG0000(	VIM/TOR1/	GO_BP
GO:00094	1	1/1117	15/20610	0.566605	0.875141	1.230081	ENSG0000(	CD9	GO_BP

GO:00098	1	1/1117	15/20610	0.566605	0.875141	1.230081	ENSG0000	SULT1A1	GO_BP
GO:00181	1	1/1117	15/20610	0.566605	0.875141	1.230081	ENSG0000	GPX1	GO_BP
GO:00480	1	1/1117	15/20610	0.566605	0.875141	1.230081	ENSG0000	BCL2L11	GO_BP
GO:00602	1	1/1117	15/20610	0.566605	0.875141	1.230081	ENSG0000	RAC2	GO_BP
GO:00616	1	1/1117	15/20610	0.566605	0.875141	1.230081	ENSG0000	HSPA8	GO_BP
GO:00719	1	1/1117	15/20610	0.566605	0.875141	1.230081	ENSG0000	CEBPA	GO_BP
GO:00900	1	1/1117	15/20610	0.566605	0.875141	1.230081	ENSG0000	TRMT112	GO_BP
GO:19047	1	1/1117	15/20610	0.566605	0.875141	1.230081	ENSG0000	VDR	GO_BP
GO:20002	1	1/1117	15/20610	0.566605	0.875141	1.230081	ENSG0000	RETN	GO_BP
GO:00310	11	11/1117	207/2061	0.570645	0.878523	0.980499	ENSG0000	GPX1/NIN	GO_BP
GO:00451	12	12/1117	226/2061	0.571176	0.878523	0.97971	ENSG0000	ARRB2/HM	GO_BP
GO:00030	20	20/1117	377/2061	0.572285	0.878523	0.978844	ENSG0000	PBX2/NA/	GO_BP
GO:00427	2	2/1117	35/20610	0.572692	0.878523	1.054355	ENSG0000	OPRL1/NA	GO_BP
GO:00701	2	2/1117	35/20610	0.572692	0.878523	1.054355	ENSG0000	SRGN/GAT	GO_BP
GO:19013	2	2/1117	35/20610	0.572692	0.878523	1.054355	ENSG0000	KCNE3/BI	GO_BP
GO:19021	2	2/1117	35/20610	0.572692	0.878523	1.054355	ENSG0000	TRIM25/T	GO_BP
GO:00076	7	7/1117	132/2061	0.578201	0.885402	0.978473	ENSG0000	RGS14/SR	GO_BP
GO:00108	3	3/1117	55/20610	0.578961	0.885519	1.00643	ENSG0000	NFKBIA/Z	GO_BP
GO:00420	8	8/1117	152/2061	0.585018	0.885594	0.971116	ENSG0000	TYROBP/M	GO_BP
GO:20002	4	4/1117	75/20610	0.585159	0.885594	0.984064	ENSG0000	SERPING1	GO_BP
GO:00102	2	2/1117	36/20610	0.588167	0.885594	1.025067	ENSG0000	SERP1/HY	GO_BP
GO:00328	2	2/1117	36/20610	0.588167	0.885594	1.025067	ENSG0000	ARL6IP1/	GO_BP
GO:00519	2	2/1117	36/20610	0.588167	0.885594	1.025067	ENSG0000	ARL6IP1/	GO_BP
GO:00439	11	11/1117	210/2061	0.590006	0.885594	0.966492	ENSG0000	DHX58/PO	GO_BP
GO:00219	1	1/1117	16/20610	0.590111	0.885594	1.153201	ENSG0000	HES1	GO_BP
GO:00456	1	1/1117	16/20610	0.590111	0.885594	1.153201	ENSG0000	VDR	GO_BP
GO:19002	1	1/1117	16/20610	0.590111	0.885594	1.153201	ENSG0000	DHX58	GO_BP
GO:19048	1	1/1117	16/20610	0.590111	0.885594	1.153201	ENSG0000	NOP10	GO_BP
GO:19054	7	7/1117	134/2061	0.594237	0.889468	0.963869	ENSG0000	STX4/ITG	GO_BP
GO:00427	6	6/1117	115/2061	0.596758	0.891697	0.962672	ENSG0000	SIAH2/DD	GO_BP
GO:00105	2	2/1117	37/20610	0.603222	0.896234	0.997363	ENSG0000	CCR2/PTG	GO_BP
GO:00509	2	2/1117	37/20610	0.603222	0.896234	0.997363	ENSG0000	CD63/HPS	GO_BP
GO:00312	3	3/1117	57/20610	0.603424	0.896234	0.971116	ENSG0000	CD81/TNF	GO_BP
GO:00226	6	6/1117	116/2061	0.605252	0.898466	0.954373	ENSG0000	ACO1/OPR	GO_BP
GO:00302	5	5/1117	97/20610	0.609714	0.899433	0.951093	ENSG0000	SRGN/ID3	GO_BP
GO:00303	10	10/1117	194/2061	0.610353	0.899433	0.951093	ENSG0000	SDCBP/S1	GO_BP
GO:19018	9	9/1117	175/2061	0.612165	0.899433	0.948919	ENSG0000	RGS2/PIM	GO_BP
GO:00189	1	1/1117	17/20610	0.612343	0.899433	1.085365	ENSG0000	TXN	GO_BP
GO:00316	1	1/1117	17/20610	0.612343	0.899433	1.085365	ENSG0000	S100B	GO_BP
GO:00517	1	1/1117	17/20610	0.612343	0.899433	1.085365	ENSG0000	BCL2L11	GO_BP
GO:00518	1	1/1117	17/20610	0.612343	0.899433	1.085365	ENSG0000	BCL2L11	GO_BP
GO:20002	3	3/1117	58/20610	0.615298	0.901402	0.954373	ENSG0000	DUSP1/CA	GO_BP
GO:00468	7	7/1117	137/2061	0.617695	0.901402	0.942762	ENSG0000	IRS2/SRI	GO_BP
GO:00395	2	2/1117	38/20610	0.617856	0.901402	0.971116	ENSG0000	DHX58/DD	GO_BP
GO:20010	5	5/1117	98/20610	0.618834	0.902321	0.941388	ENSG0000	PRKCD/WA	GO_BP
GO:00466	6	6/1117	118/2061	0.621942	0.906344	0.938197	ENSG0000	ARRB2/RE	GO_BP
GO:00486	23	23/1117	445/2061	0.623764	0.907942	0.953658	ENSG0000	PSAP/JUN	GO_BP
GO:00068	7	7/1117	138/2061	0.625348	0.907942	0.935931	ENSG0000	MGST3/SU	GO_BP
GO:00717	8	8/1117	158/2061	0.628885	0.907942	0.934238	ENSG0000	POLR2C/Z	GO_BP
GO:00422	10	10/1117	197/2061	0.62971	0.907942	0.93661	ENSG0000	ARRB2/HL	GO_BP
GO:00018	6	6/1117	119/2061	0.630135	0.907942	0.930313	ENSG0000	JUNB/SRF	GO_BP
GO:00070	2	2/1117	39/20610	0.632071	0.907942	0.946216	ENSG0000	CHMP2A/K	GO_BP
GO:00326	2	2/1117	39/20610	0.632071	0.907942	0.946216	ENSG0000	IRF4/CEB	GO_BP
GO:00075	1	1/1117	18/20610	0.63337	0.907942	1.025067	ENSG0000	SERPING1	GO_BP

GO:003165	1	1/1117	18/20610	0.63337	0.907942	1.025067	ENSG00000	PTGS2	GO_BP
GO:190011	1	1/1117	18/20610	0.63337	0.907942	1.025067	ENSG00000	ZC3H12A	GO_BP
GO:004239	24	24/1117	467/20610	0.636278	0.91092	0.948242	ENSG00000	DGKI/IFI	GO_BP
GO:005079	4	4/1117	80/20610	0.636327	0.91092	0.92256	ENSG00000	RETN/OPR	GO_BP
GO:000657	11	11/1117	218/20610	0.6396	0.913589	0.931024	ENSG00000	GPX1/CRT	GO_BP
GO:190401	12	12/1117	238/20610	0.643106	0.917428	0.930313	ENSG00000	RHOB/ZC3	GO_BP
GO:004512	4	4/1117	81/20610	0.646053	0.917428	0.911171	ENSG00000	ADAM8/FA	GO_BP
GO:004516	15	15/1117	297/20610	0.648188	0.917428	0.931879	ENSG00000	MCL1/KDM	GO_BP
GO:004864	3	3/1117	61/20610	0.649456	0.917428	0.907436	ENSG00000	PIM1/HES	GO_BP
GO:000737	1	1/1117	19/20610	0.653258	0.917428	0.971116	ENSG00000	DVL2	GO_BP
GO:000963	1	1/1117	19/20610	0.653258	0.917428	0.971116	ENSG00000	ZC3H12A	GO_BP
GO:007257	1	1/1117	19/20610	0.653258	0.917428	0.971116	ENSG00000	CEBPB	GO_BP
GO:190027	1	1/1117	19/20610	0.653258	0.917428	0.971116	ENSG00000	TYROBP	GO_BP
GO:006032	2	2/1117	41/20610	0.659254	0.920868	0.900059	ENSG00000	RRAS/CSR	GO_BP
GO:003503	3	3/1117	62/20610	0.660349	0.921397	0.8928	ENSG00000	CD9/FOLR	GO_BP
GO:004863	10	10/1117	202/20610	0.660814	0.921549	0.913426	ENSG00000	TWF2/PIM	GO_BP
GO:000264	1	1/1117	20/20610	0.672068	0.923314	0.92256	ENSG00000	CD274	GO_BP
GO:001098	1	1/1117	20/20610	0.672068	0.923314	0.92256	ENSG00000	LRPAP1	GO_BP
GO:003165	1	1/1117	20/20610	0.672068	0.923314	0.92256	ENSG00000	PTGS2	GO_BP
GO:003606	1	1/1117	20/20610	0.672068	0.923314	0.92256	ENSG00000	POFUT2	GO_BP
GO:004263	1	1/1117	20/20610	0.672068	0.923314	0.92256	ENSG00000	TRADD	GO_BP
GO:190487	1	1/1117	20/20610	0.672068	0.923314	0.92256	ENSG00000	NOP10	GO_BP
GO:190011	2	2/1117	42/20610	0.67223	0.923314	0.878629	ENSG00000	ZC3H12A/	GO_BP
GO:014005	9	9/1117	185/20610	0.677515	0.928851	0.897626	ENSG00000	TUBA1A/S	GO_BP
GO:004512	2	2/1117	43/20610	0.684802	0.931245	0.858196	ENSG00000	ADAM8/S1	GO_BP
GO:006096	2	2/1117	43/20610	0.684802	0.931245	0.858196	ENSG00000	ZC3H12A/	GO_BP
GO:000241	1	1/1117	21/20610	0.689858	0.931245	0.878629	ENSG00000	CD274	GO_BP
GO:003581	1	1/1117	21/20610	0.689858	0.931245	0.878629	ENSG00000	GNAI2	GO_BP
GO:006071	1	1/1117	21/20610	0.689858	0.931245	0.878629	ENSG00000	SOCS3	GO_BP
GO:190211	4	4/1117	86/20610	0.692062	0.932521	0.858196	ENSG00000	SDCBP/RH	GO_BP
GO:001063	15	15/1117	307/20610	0.697306	0.936666	0.901525	ENSG00000	TMSB4X/A	GO_BP
GO:007155	13	13/1117	268/20610	0.699059	0.938778	0.895021	ENSG00000	SDCBP/ZN	GO_BP
GO:004434	1	1/1117	22/20610	0.706684	0.941942	0.838691	ENSG00000	SERPINB1	GO_BP
GO:004852	1	1/1117	22/20610	0.706684	0.941942	0.838691	ENSG00000	RETN	GO_BP
GO:190011	1	1/1117	22/20610	0.706684	0.941942	0.838691	ENSG00000	RNF34	GO_BP
GO:004846	9	9/1117	190/20610	0.707562	0.942041	0.874005	ENSG00000	KLF2/CEB	GO_BP
GO:001404	2	2/1117	45/20610	0.708756	0.942041	0.820054	ENSG00000	SNCA/NTS	GO_BP
GO:003164	2	2/1117	45/20610	0.708756	0.942041	0.820054	ENSG00000	TG/S100B	GO_BP
GO:004476	4	4/1117	89/20610	0.717544	0.944221	0.829268	ENSG00000	BST2/DYN	GO_BP
GO:004688	14	14/1117	292/20610	0.719388	0.944221	0.884647	ENSG00000	SLC25A5/	GO_BP
GO:002260	2	2/1117	46/20610	0.720152	0.944221	0.802226	ENSG00000	ARRB2/RE	GO_BP
GO:003101	2	2/1117	46/20610	0.720152	0.944221	0.802226	ENSG00000	BAK1/HES	GO_BP
GO:003238	3	3/1117	68/20610	0.720539	0.944221	0.814024	ENSG00000	TXN/BAG3	GO_BP
GO:000753	1	1/1117	23/20610	0.722598	0.944221	0.802226	ENSG00000	CITED2	GO_BP
GO:002154	1	1/1117	23/20610	0.722598	0.944221	0.802226	ENSG00000	BTG2	GO_BP
GO:004240	1	1/1117	23/20610	0.722598	0.944221	0.802226	ENSG00000	TG	GO_BP
GO:004393	1	1/1117	23/20610	0.722598	0.944221	0.802226	ENSG00000	RHOA	GO_BP
GO:007142	1	1/1117	23/20610	0.722598	0.944221	0.802226	ENSG00000	PIM1	GO_BP
GO:200077	1	1/1117	23/20610	0.722598	0.944221	0.802226	ENSG00000	BCL2L12	GO_BP
GO:000197	2	2/1117	47/20610	0.731169	0.947883	0.785158	ENSG00000	CCR2/BAK	GO_BP
GO:007084	2	2/1117	47/20610	0.731169	0.947883	0.785158	ENSG00000	EEF1A1/Z	GO_BP
GO:009034	5	5/1117	112/20610	0.732478	0.947883	0.823715	ENSG00000	B2M/NA/H	GO_BP
GO:000761	1	1/1117	24/20610	0.737649	0.947883	0.7688	ENSG00000	UBE2Q1	GO_BP
GO:001088	1	1/1117	24/20610	0.737649	0.947883	0.7688	ENSG00000	ZC3H12A	GO_BP

GO:00435	1	1/1117	24/20610	0.737649	0.947883	0.7688	ENSG0000	NTSR1	GO_BP
GO:00018	2	2/1117	48/20610	0.741815	0.949238	0.7688	ENSG0000	JUNB/SRF	GO_BP
GO:00485	2	2/1117	48/20610	0.741815	0.949238	0.7688	ENSG0000	OPRL1/NA	GO_BP
GO:00602	2	2/1117	48/20610	0.741815	0.949238	0.7688	ENSG0000	TSPO/HES	GO_BP
GO:00612	2	2/1117	48/20610	0.741815	0.949238	0.7688	ENSG0000	RHOA/MSN	GO_BP
GO:00075	13	13/1117	277/2061	0.742062	0.94932	0.865941	ENSG0000	ARRB2/HM	GO_BP
GO:00434	16	16/1117	337/2061	0.742525	0.949677	0.876022	ENSG0000	TRMT112/	GO_BP
GO:00459	14	14/1117	298/2061	0.746349	0.951594	0.866835	ENSG0000	SDCBP/S1	GO_BP
GO:00094	1	1/1117	25/20610	0.751884	0.951594	0.738048	ENSG0000	MPST	GO_BP
GO:00105	1	1/1117	25/20610	0.751884	0.951594	0.738048	ENSG0000	MOV10	GO_BP
GO:00105	1	1/1117	25/20610	0.751884	0.951594	0.738048	ENSG0000	MOV10	GO_BP
GO:00310	1	1/1117	25/20610	0.751884	0.951594	0.738048	ENSG0000	WNT10A	GO_BP
GO:00456	1	1/1117	25/20610	0.751884	0.951594	0.738048	ENSG0000	HES1	GO_BP
GO:19020	1	1/1117	25/20610	0.751884	0.951594	0.738048	ENSG0000	ZC3H12A	GO_BP
GO:00080	2	2/1117	49/20610	0.752099	0.951594	0.753111	ENSG0000	BSG/TNN	GO_BP
GO:00343	15	15/1117	320/2061	0.754721	0.954446	0.8649	ENSG0000	ZNF703/F	GO_BP
GO:00447	3	3/1117	72/20610	0.755834	0.954689	0.7688	ENSG0000	POLE4/UP	GO_BP
GO:19043	3	3/1117	72/20610	0.755834	0.954689	0.7688	ENSG0000	STX4/LGA	GO_BP
GO:00230	24	24/1117	500/2061	0.759877	0.955073	0.885658	ENSG0000	SLC25A5/	GO_BP
GO:00468	3	3/1117	73/20610	0.764074	0.955073	0.758269	ENSG0000	PIM3/HMG	GO_BP
GO:00108	1	1/1117	26/20610	0.765347	0.955073	0.709662	ENSG0000	NFKBIA	GO_BP
GO:00603	1	1/1117	26/20610	0.765347	0.955073	0.709662	ENSG0000	SRF	GO_BP
GO:00607	1	1/1117	26/20610	0.765347	0.955073	0.709662	ENSG0000	SOCS3	GO_BP
GO:00971	1	1/1117	26/20610	0.765347	0.955073	0.709662	ENSG0000	HES1	GO_BP
GO:00083	6	6/1117	139/2061	0.770732	0.958867	0.796455	ENSG0000	PSAP/TLR	GO_BP
GO:00468	4	4/1117	96/20610	0.770863	0.958867	0.7688	ENSG0000	ADAM8/TP	GO_BP
GO:00440	2	2/1117	51/20610	0.771608	0.958867	0.723577	ENSG0000	PSAP/S10	GO_BP
GO:00109	5	5/1117	118/2061	0.772912	0.959202	0.781831	ENSG0000	TLR2/SRF,	GO_BP
GO:00083	1	1/1117	27/20610	0.778081	0.960035	0.683378	ENSG0000	RGS14	GO_BP
GO:00301	1	1/1117	27/20610	0.778081	0.960035	0.683378	ENSG0000	TLR4	GO_BP
GO:00316	1	1/1117	27/20610	0.778081	0.960035	0.683378	ENSG0000	EIF4A3	GO_BP
GO:00513	1	1/1117	27/20610	0.778081	0.960035	0.683378	ENSG0000	MUS81	GO_BP
GO:00606	1	1/1117	27/20610	0.778081	0.960035	0.683378	ENSG0000	SOCS3	GO_BP
GO:00723	1	1/1117	27/20610	0.778081	0.960035	0.683378	ENSG0000	SERPING1	GO_BP
GO:19000	1	1/1117	27/20610	0.778081	0.960035	0.683378	ENSG0000	TLR4	GO_BP
GO:19035	3	3/1117	75/20610	0.779875	0.960035	0.738048	ENSG0000	RGS2/RHO	GO_BP
GO:19052	2	2/1117	52/20610	0.780852	0.960035	0.709662	ENSG0000	ARRB2/KA	GO_BP
GO:00310	6	6/1117	141/2061	0.782291	0.961408	0.785158	ENSG0000	CHMP2A/C	GO_BP
GO:00600	4	4/1117	98/20610	0.784558	0.963045	0.753111	ENSG0000	PYGO2/MM	GO_BP
GO:00466	7	7/1117	163/2061	0.786335	0.963045	0.792383	ENSG0000	HMGB2/BC	GO_BP
GO:20001	2	2/1117	53/20610	0.789765	0.963045	0.696272	ENSG0000	GNAI2/NA	GO_BP
GO:00026	1	1/1117	28/20610	0.790124	0.963045	0.658972	ENSG0000	CD274	GO_BP
GO:00067	1	1/1117	28/20610	0.790124	0.963045	0.658972	ENSG0000	AHCYL1	GO_BP
GO:00508	1	1/1117	28/20610	0.790124	0.963045	0.658972	ENSG0000	TLR4	GO_BP
GO:00605	1	1/1117	28/20610	0.790124	0.963045	0.658972	ENSG0000	CRIP1	GO_BP
GO:00604	3	3/1117	77/20610	0.794794	0.96698	0.718878	ENSG0000	HES1/CIT	GO_BP
GO:00488	9	9/1117	207/2061	0.795681	0.96698	0.802226	ENSG0000	PSAP/CEB	GO_BP
GO:00017	2	2/1117	54/20610	0.798356	0.96698	0.683378	ENSG0000	MCL1/HES	GO_BP
GO:00101	2	2/1117	54/20610	0.798356	0.96698	0.683378	ENSG0000	RRAS/CSR	GO_BP
GO:19035	2	2/1117	54/20610	0.798356	0.96698	0.683378	ENSG0000	PPP1R15A,	GO_BP
GO:00485	1	1/1117	29/20610	0.801514	0.96698	0.636249	ENSG0000	CFAP20	GO_BP
GO:00600	1	1/1117	29/20610	0.801514	0.96698	0.636249	ENSG0000	PFN1	GO_BP
GO:19052	1	1/1117	29/20610	0.801514	0.96698	0.636249	ENSG0000	RGS2	GO_BP
GO:00343	3	3/1117	78/20610	0.801931	0.96698	0.709662	ENSG0000	HNRNPK/A	GO_BP

GO:00604	5	5/1117	123/2061	0.802896	0.967918	0.750049	ENSG0000	DUSP6/RG	GO_BP
GO:00073	22	22/1117	474/2061	0.803097	0.967936	0.856385	ENSG0000	PBX2/NA/	GO_BP
GO:00341	4	4/1117	101/2061	0.803869	0.968415	0.730741	ENSG0000	ADAM8/IL	GO_BP
GO:00604	8	8/1117	188/2061	0.805083	0.968431	0.785158	ENSG0000	TWF2/PFN	GO_BP
GO:00085	2	2/1117	55/20610	0.806636	0.968431	0.670953	ENSG0000	RGS14/HM	GO_BP
GO:00030	1	1/1117	30/20610	0.812287	0.968431	0.61504	ENSG0000	GAA	GO_BP
GO:00028	2	2/1117	56/20610	0.814611	0.970309	0.658972	ENSG0000	DHX58/CD	GO_BP
GO:00486	4	4/1117	104/2061	0.82175	0.971863	0.709662	ENSG0000	PIM1/ARR	GO_BP
GO:00032	1	1/1117	31/20610	0.822475	0.971863	0.5952	ENSG0000	TCAP	GO_BP
GO:00064	1	1/1117	31/20610	0.822475	0.971863	0.5952	ENSG0000	KDM6B	GO_BP
GO:00359	1	1/1117	31/20610	0.822475	0.971863	0.5952	ENSG0000	FOXO4	GO_BP
GO:00506	1	1/1117	31/20610	0.822475	0.971863	0.5952	ENSG0000	DHX58	GO_BP
GO:00903	1	1/1117	31/20610	0.822475	0.971863	0.5952	ENSG0000	BCL2L12	GO_BP
GO:19054	1	1/1117	31/20610	0.822475	0.971863	0.5952	ENSG0000	GDI1	GO_BP
GO:19018	4	4/1117	105/2061	0.827403	0.975027	0.702903	ENSG0000	PIM1/ARR	GO_BP
GO:01101	3	3/1117	82/20610	0.828424	0.975027	0.675044	ENSG0000	PIM1/VDR	GO_BP
GO:19038	5	5/1117	128/2061	0.829659	0.975027	0.72075	ENSG0000	ABHD17A/	GO_BP
GO:00190	1	1/1117	32/20610	0.832111	0.975027	0.5766	ENSG0000	UBE2Q1	GO_BP
GO:00445	1	1/1117	32/20610	0.832111	0.975027	0.5766	ENSG0000	MPST	GO_BP
GO:00602	1	1/1117	32/20610	0.832111	0.975027	0.5766	ENSG0000	TSPO	GO_BP
GO:00352	6	6/1117	152/2061	0.838073	0.978204	0.728337	ENSG0000	KLF2/STA	GO_BP
GO:00107	1	1/1117	33/20610	0.841224	0.978204	0.559128	ENSG0000	ADIPOR1	GO_BP
GO:01200	4	4/1117	108/2061	0.843475	0.978204	0.683378	ENSG0000	TWF2/PFN	GO_BP
GO:00439	2	2/1117	60/20610	0.843641	0.978204	0.61504	ENSG0000	CTTN/NA	GO_BP
GO:00508	2	2/1117	60/20610	0.843641	0.978204	0.61504	ENSG0000	GAA/TCAP	GO_BP
GO:00095	8	8/1117	198/2061	0.847164	0.978204	0.745503	ENSG0000	CD9/YBX3	GO_BP
GO:00070	5	5/1117	132/2061	0.848876	0.978204	0.698909	ENSG0000	CHMP2A/C	GO_BP
GO:00315	1	1/1117	34/20610	0.849843	0.978204	0.542683	ENSG0000	DUSP1	GO_BP
GO:00482	1	1/1117	34/20610	0.849843	0.978204	0.542683	ENSG0000	TSPO	GO_BP
GO:00508	1	1/1117	34/20610	0.849843	0.978204	0.542683	ENSG0000	LGALS3	GO_BP
GO:00716	7	7/1117	177/2061	0.850481	0.978204	0.729709	ENSG0000	KLF2/RHO	GO_BP
GO:00347	6	6/1117	156/2061	0.855261	0.978694	0.709662	ENSG0000	DHRS9/SU	GO_BP
GO:00516	6	6/1117	156/2061	0.855261	0.978694	0.709662	ENSG0000	TYROBP/I	GO_BP
GO:00607	3	3/1117	87/20610	0.857215	0.978694	0.636249	ENSG0000	CARD16/I	GO_BP
GO:00071	1	1/1117	35/20610	0.857995	0.978694	0.527177	ENSG0000	PRKAR1A	GO_BP
GO:00426	1	1/1117	35/20610	0.857995	0.978694	0.527177	ENSG0000	TRADD	GO_BP
GO:00601	1	1/1117	35/20610	0.857995	0.978694	0.527177	ENSG0000	TYROBP	GO_BP
GO:00700	1	1/1117	35/20610	0.857995	0.978694	0.527177	ENSG0000	CHMP2B	GO_BP
GO:00071	2	2/1117	63/20610	0.862637	0.982163	0.585753	ENSG0000	MUS81/KL	GO_BP
GO:00074	2	2/1117	63/20610	0.862637	0.982163	0.585753	ENSG0000	KCTD11/N	GO_BP
GO:00108	19	19/1117	436/2061	0.865456	0.982635	0.804066	ENSG0000	HNRNPK/P	GO_BP
GO:00164	1	1/1117	36/20610	0.865704	0.982635	0.512534	ENSG0000	FURIN	GO_BP
GO:00025	2	2/1117	64/20610	0.868483	0.983636	0.5766	ENSG0000	TCF3/HMG	GO_BP
GO:00513	7	7/1117	182/2061	0.869243	0.984017	0.709662	ENSG0000	TXNIP/CA	GO_BP
GO:00097	3	3/1117	90/20610	0.872383	0.984017	0.61504	ENSG0000	ETS2/SRF	GO_BP
GO:00217	3	3/1117	90/20610	0.872383	0.984017	0.61504	ENSG0000	KDM6B/SR	GO_BP
GO:19038	1	1/1117	37/20610	0.872995	0.984017	0.498681	ENSG0000	GNAI2	GO_BP
GO:00508	3	3/1117	91/20610	0.877113	0.985365	0.608282	ENSG0000	HCAR2/RH	GO_BP
GO:00095	5	5/1117	139/2061	0.878168	0.985365	0.663713	ENSG0000	ARRB2/UN	GO_BP
GO:00600	1	1/1117	38/20610	0.879891	0.985365	0.485558	ENSG0000	PIM1	GO_BP
GO:00032	4	4/1117	116/2061	0.880272	0.985365	0.636249	ENSG0000	PLXND1/H	GO_BP
GO:00550	4	4/1117	116/2061	0.880272	0.985365	0.636249	ENSG0000	RGS2/PIM	GO_BP
GO:00506	17	17/1117	401/2061	0.881045	0.986018	0.782221	ENSG0000	B2M/NA/Z	GO_BP
GO:00073	6	6/1117	163/2061	0.881717	0.986556	0.679186	ENSG0000	CD9/FOLR	GO_BP

GO:00015:	1	1/1117	39/20610	0.886412	0.987336	0.473108	ENSG0000(	CFAP20	GO_BP
GO:00069:	1	1/1117	39/20610	0.886412	0.987336	0.473108	ENSG0000(	HMGB2	GO_BP
GO:00486	4	4/1117	118/2061(	0.888207	0.98838	0.625465	ENSG0000(	RGS2/SEM	GO_BP
GO:00095	5	5/1117	142/2061(	0.889159	0.98838	0.64969	ENSG0000(	ARRB2/UN(	GO_BP
GO:00400	2	2/1117	68/20610	0.88965	0.98838	0.542683	ENSG0000(	STAT5A/S(	GO_BP
GO:00450:	2	2/1117	68/20610	0.88965	0.98838	0.542683	ENSG0000(	MAX/FOXO	GO_BP
GO:19048	2	2/1117	68/20610	0.88965	0.98838	0.542683	ENSG0000(	EIF4A3/AI	GO_BP
GO:00147	1	1/1117	40/20610	0.89258	0.98838	0.46128	ENSG0000(	RGS2	GO_BP
GO:20000:	1	1/1117	40/20610	0.89258	0.98838	0.46128	ENSG0000(	ZNF706	GO_BP
GO:00300	5	5/1117	143/2061(	0.892628	0.98838	0.645147	ENSG0000(	DYNLT1/RI	GO_BP
GO:00485	6	6/1117	167/2061(	0.894918	0.98838	0.662918	ENSG0000(	HMGB2/PY(	GO_BP
GO:00104(	7	7/1117	191/2061(	0.898112	0.98838	0.676222	ENSG0000(	PRKCD/LY(	GO_BP
GO:00431	1	1/1117	41/20610	0.898413	0.98838	0.450029	ENSG0000(	CXCR2	GO_BP
GO:00901	1	1/1117	41/20610	0.898413	0.98838	0.450029	ENSG0000(	MYC	GO_BP
GO:19001	1	1/1117	41/20610	0.898413	0.98838	0.450029	ENSG0000(	FLCN	GO_BP
GO:19018	1	1/1117	41/20610	0.898413	0.98838	0.450029	ENSG0000(	S100A10	GO_BP
GO:19013	3	3/1117	96/20610	0.898497	0.98838	0.5766	ENSG0000(	CD63/KCNI	GO_BP
GO:00336:	2	2/1117	70/20610	0.899005	0.98838	0.527177	ENSG0000(	ITGB2/TE	GO_BP
GO:00990	2	2/1117	70/20610	0.899005	0.98838	0.527177	ENSG0000(	STX3/HGN(	GO_BP
GO:19059	2	2/1117	70/20610	0.899005	0.98838	0.527177	ENSG0000(	NFKBIA/II	GO_BP
GO:00603	2	2/1117	71/20610	0.903401	0.989051	0.519752	ENSG0000(	VIM/FAM8	GO_BP
GO:00171	1	1/1117	42/20610	0.90393	0.989051	0.439314	ENSG0000(	ZFP36L2	GO_BP
GO:00400	1	1/1117	42/20610	0.90393	0.989051	0.439314	ENSG0000(	HES1	GO_BP
GO:00466:	1	1/1117	42/20610	0.90393	0.989051	0.439314	ENSG0000(	RGS2	GO_BP
GO:00108	4	4/1117	123/2061(	0.906063	0.990499	0.600039	ENSG0000(	NINJ1/S1(	GO_BP
GO:20002	2	2/1117	72/20610	0.907617	0.990921	0.512534	ENSG0000(	RETN/CITI	GO_BP
GO:00025(	1	1/1117	43/20610	0.909147	0.990921	0.429098	ENSG0000(	CD274	GO_BP
GO:00106:	4	4/1117	124/2061(	0.909315	0.990921	0.5952	ENSG0000(	RHOA/ADII	GO_BP
GO:00017	2	2/1117	73/20610	0.91166	0.992433	0.505513	ENSG0000(	KAT2A/TC	GO_BP
GO:00022(	2	2/1117	73/20610	0.91166	0.992433	0.505513	ENSG0000(	TCF3/HMGI	GO_BP
GO:00458:	3	3/1117	100/2061(	0.913126	0.992776	0.553536	ENSG0000(	HCAR2/SC	GO_BP
GO:00069(	1	1/1117	44/20610	0.914082	0.992776	0.419346	ENSG0000(	STX3	GO_BP
GO:00067(	5	5/1117	150/2061(	0.914385	0.992898	0.61504	ENSG0000(	SLC2A1/RI	GO_BP
GO:00192:	2	2/1117	74/20610	0.915537	0.993069	0.498681	ENSG0000(	S1PR1/SOI	GO_BP
GO:00073:	1	1/1117	45/20610	0.918748	0.993069	0.410027	ENSG0000(	ALDOA	GO_BP
GO:00323	1	1/1117	45/20610	0.918748	0.993069	0.410027	ENSG0000(	VDR	GO_BP
GO:00343	1	1/1117	45/20610	0.918748	0.993069	0.410027	ENSG0000(	HNRNPK	GO_BP
GO:00436	1	1/1117	45/20610	0.918748	0.993069	0.410027	ENSG0000(	VDR	GO_BP
GO:00974	11	11/1117	290/2061(	0.919835	0.993069	0.699873	ENSG0000(	IRS2/EPHI	GO_BP
GO:19013	7	7/1117	200/2061(	0.921387	0.993069	0.645792	ENSG0000(	KLF2/RHO	GO_BP
GO:20002	5	5/1117	153/2061(	0.922456	0.993069	0.602981	ENSG0000(	DUSP1/RE	GO_BP
GO:00215	1	1/1117	46/20610	0.923161	0.993069	0.401113	ENSG0000(	HES1	GO_BP
GO:00519	1	1/1117	46/20610	0.923161	0.993069	0.401113	ENSG0000(	DUSP1	GO_BP
GO:00988(	1	1/1117	46/20610	0.923161	0.993069	0.401113	ENSG0000(	CARM1	GO_BP
GO:00032(	4	4/1117	130/2061(	0.926805	0.993642	0.567729	ENSG0000(	SRF/HES1	GO_BP
GO:00076	1	1/1117	47/20610	0.927335	0.993642	0.392579	ENSG0000(	UBE2Q1	GO_BP
GO:00971(	8	8/1117	226/2061(	0.927531	0.993646	0.65314	ENSG0000(	SAT1/PRK(	GO_BP
GO:00198:	6	6/1117	180/2061(	0.929467	0.993753	0.61504	ENSG0000(	ZNF706/P(	GO_BP
GO:00323(	1	1/1117	48/20610	0.931282	0.993753	0.3844	ENSG0000(	IRS2	GO_BP
GO:00336:	1	1/1117	48/20610	0.931282	0.993753	0.3844	ENSG0000(	TESC	GO_BP
GO:00519	3	3/1117	106/2061(	0.931497	0.993753	0.522204	ENSG0000(	DUSP1/RC(	GO_BP
GO:00506	6	6/1117	181/2061(	0.931655	0.993753	0.611642	ENSG0000(	B2M/NA/T	GO_BP
GO:00991	6	6/1117	181/2061(	0.931655	0.993753	0.611642	ENSG0000(	ABHD17A/	GO_BP
GO:00991	1	1/1117	50/20610	0.938546	0.995147	0.369024	ENSG0000(	SNCA	GO_BP

GO:00516[illegible]	1	1/1117	51/20610	0.941884	0.995188	0.361788	ENSG0000[illegible]	DYNLT1	GO_BP
GO:00609[illegible]	5	5/1117	162/2061[illegible]	0.942768	0.995337	0.569482	ENSG0000[illegible]	POLR2C/Z[illegible]	GO_BP
GO:00506[illegible]	7	7/1117	211/2061[illegible]	0.94345	0.995337	0.612125	ENSG0000[illegible]	ZNF703/S[illegible]	GO_BP
GO:00020[illegible]	2	2/1117	83/20610	0.943898	0.995337	0.444607	ENSG0000[illegible]	VIM/PYGO[illegible]	GO_BP
GO:00060[illegible]	2	2/1117	83/20610	0.943898	0.995337	0.444607	ENSG0000[illegible]	TALDO1/T[illegible]	GO_BP
GO:00148[illegible]	2	2/1117	83/20610	0.943898	0.995337	0.444607	ENSG0000[illegible]	PIM1/PRK[illegible]	GO_BP
GO:00720[illegible]	2	2/1117	84/20610	0.94642	0.995342	0.439314	ENSG0000[illegible]	HES1/MYC	GO_BP
GO:00424[illegible]	3	3/1117	113/2061[illegible]	0.948378	0.995827	0.489855	ENSG0000[illegible]	TSPO/VDR[illegible]	GO_BP
GO:00099[illegible]	1	1/1117	55/20610	0.953523	0.995974	0.335477	ENSG0000[illegible]	ZGLP1	GO_BP
GO:00148[illegible]	1	1/1117	57/20610	0.958437	0.998893	0.323705	ENSG0000[illegible]	TCAP	GO_BP
GO:00026[illegible]	1	1/1117	58/20610	0.960696	0.999923	0.318124	ENSG0000[illegible]	DUSP1	GO_BP
GO:00600[illegible]	1	1/1117	58/20610	0.960696	0.999923	0.318124	ENSG0000[illegible]	PIM1	GO_BP
GO:00517[illegible]	2	2/1117	91/20610	0.961274	0.999923	0.405521	ENSG0000[illegible]	RHOA/VEG[illegible]	GO_BP
GO:00063[illegible]	3	3/1117	120/2061[illegible]	0.961317	0.999923	0.46128	ENSG0000[illegible]	HMGA1/CH[illegible]	GO_BP
GO:00032[illegible]	5	5/1117	175/2061[illegible]	0.963691	0.999923	0.527177	ENSG0000[illegible]	PLXND1/S[illegible]	GO_BP
GO:00019[illegible]	2	2/1117	93/20610	0.964734	0.999923	0.3968	ENSG0000[illegible]	TRADD/WN[illegible]	GO_BP
GO:00513[illegible]	2	2/1117	93/20610	0.964734	0.999923	0.3968	ENSG0000[illegible]	DUSP1/MU[illegible]	GO_BP
GO:00426[illegible]	3	3/1117	123/2061[illegible]	0.965871	0.999923	0.450029	ENSG0000[illegible]	TRADD/PT[illegible]	GO_BP
GO:00164[illegible]	1	1/1117	62/20610	0.968569	0.999923	0.2976	ENSG0000[illegible]	TCF3	GO_BP
GO:00224[illegible]	2	2/1117	96/20610	0.969372	0.999923	0.3844	ENSG0000[illegible]	TRADD/WN[illegible]	GO_BP
GO:00224[illegible]	2	2/1117	96/20610	0.969372	0.999923	0.3844	ENSG0000[illegible]	TRADD/WN[illegible]	GO_BP
GO:00076[illegible]	1	1/1117	63/20610	0.970278	0.999923	0.292876	ENSG0000[illegible]	UNC119	GO_BP
GO:00328[illegible]	1	1/1117	63/20610	0.970278	0.999923	0.292876	ENSG0000[illegible]	HES1	GO_BP
GO:00224[illegible]	13	13/1117	377/2061[illegible]	0.972387	0.999923	0.636249	ENSG0000[illegible]	ARRB2/HM[illegible]	GO_BP
GO:00163[illegible]	8	8/1117	259/2061[illegible]	0.972944	0.999923	0.569922	ENSG0000[illegible]	DYNLT1/R[illegible]	GO_BP
GO:00508[illegible]	8	8/1117	261/2061[illegible]	0.974594	0.999923	0.565554	ENSG0000[illegible]	ABHD17A/[illegible]	GO_BP
GO:00902[illegible]	10	10/1117	310/2061[illegible]	0.974966	0.999923	0.5952	ENSG0000[illegible]	SDCBP/ZN[illegible]	GO_BP
GO:00097[illegible]	3	3/1117	134/2061[illegible]	0.978599	0.999923	0.413087	ENSG0000[illegible]	SRF/BBS5[illegible]	GO_BP
GO:00344[illegible]	2	2/1117	105/2061[illegible]	0.98002	0.999923	0.351452	ENSG0000[illegible]	IRS2/ADI[illegible]	GO_BP
GO:00609[illegible]	2	2/1117	106/2061[illegible]	0.980954	0.999923	0.348136	ENSG0000[illegible]	ARF1/DBN[illegible]	GO_BP
GO:00504[illegible]	1	1/1117	71/20610	0.980996	0.999923	0.259876	ENSG0000[illegible]	SNCA	GO_BP
GO:00070[illegible]	9	9/1117	296/2061[illegible]	0.98165	0.999923	0.561016	ENSG0000[illegible]	BLOC1S4/[illegible]	GO_BP
GO:00072[illegible]	8	8/1117	278/2061[illegible]	0.985315	0.999923	0.53097	ENSG0000[illegible]	HMGB2/RP[illegible]	GO_BP
GO:00988[illegible]	8	8/1117	278/2061[illegible]	0.985315	0.999923	0.53097	ENSG0000[illegible]	DUSP1/CH[illegible]	GO_BP
GO:19030[illegible]	1	1/1117	77/20610	0.986413	0.999923	0.239626	ENSG0000[illegible]	TLR4	GO_BP
GO:00902[illegible]	4	4/1117	175/2061[illegible]	0.9871	0.999923	0.421742	ENSG0000[illegible]	VWC2L/UB[illegible]	GO_BP
GO:00442[illegible]	16	16/1117	476/2061[illegible]	0.987286	0.999923	0.620209	ENSG0000[illegible]	NAGK/IRS[illegible]	GO_BP
GO:00519[illegible]	2	2/1117	115/2061[illegible]	0.987653	0.999923	0.320891	ENSG0000[illegible]	TLR2/SNC[illegible]	GO_BP
GO:00216[illegible]	1	1/1117	80/20610	0.988512	0.999923	0.23064	ENSG0000[illegible]	HES1	GO_BP
GO:00335[illegible]	1	1/1117	80/20610	0.988512	0.999923	0.23064	ENSG0000[illegible]	TSPO	GO_BP
GO:00215[illegible]	1	1/1117	84/20610	0.990816	0.999923	0.219657	ENSG0000[illegible]	HES1	GO_BP
GO:00977[illegible]	1	1/1117	91/20610	0.993793	0.999923	0.202761	ENSG0000[illegible]	SLIRP	GO_BP
GO:01400[illegible]	4	4/1117	193/2061[illegible]	0.993873	0.999923	0.382408	ENSG0000[illegible]	MUS81/CA[illegible]	GO_BP
GO:00619[illegible]	2	2/1117	137/2061[illegible]	0.995802	0.999923	0.269361	ENSG0000[illegible]	MUS81/KL[illegible]	GO_BP
GO:00019[illegible]	4	4/1117	203/2061[illegible]	0.995988	0.999923	0.363571	ENSG0000[illegible]	STAT5A/H[illegible]	GO_BP
GO:00451[illegible]	1	1/1117	99/20610	0.996034	0.999923	0.186376	ENSG0000[illegible]	MUS81	GO_BP
GO:00717[illegible]	3	3/1117	177/2061[illegible]	0.996845	0.999923	0.312732	ENSG0000[illegible]	VWC2L/UB[illegible]	GO_BP
GO:19030[illegible]	4	4/1117	209/2061[illegible]	0.996897	0.999923	0.353133	ENSG0000[illegible]	MUS81/CA[illegible]	GO_BP
GO:00017[illegible]	1	1/1117	108/2061[illegible]	0.997604	0.999923	0.170845	ENSG0000[illegible]	ISL2	GO_BP
GO:00720[illegible]	2	2/1117	150/2061[illegible]	0.997804	0.999923	0.246016	ENSG0000[illegible]	HES1/MYC	GO_BP
GO:00508[illegible]	12	12/1117	451/2061[illegible]	0.998565	0.999923	0.490941	ENSG0000[illegible]	PFN1/ABH[illegible]	GO_BP
GO:00600[illegible]	2	2/1117	161/2061[illegible]	0.998738	0.999923	0.229208	ENSG0000[illegible]	MAX/IMPD[illegible]	GO_BP
GO:00513[illegible]	5	5/1117	275/2061[illegible]	0.999311	0.999923	0.335477	ENSG0000[illegible]	DUSP1/MU[illegible]	GO_BP
GO:00718[illegible]	3	3/1117	230/2061[illegible]	0.999741	0.999923	0.240668	ENSG0000[illegible]	CD63/KCN[illegible]	GO_BP

GO:00988(	33	33/1164	101/2173'	9.32E-18	2.86E-15	6.101536	ENSG0000(	NDUFA7/C(	GO_CC
GO:00704(	35	35/1164	116/2173'	1.66E-17	3.40E-15	5.634517	ENSG0000(	NDUFA7/C(	GO_CC
GO:00306(	55	55/1164	283/2173'	5.41E-17	4.78E-15	3.6293	ENSG0000(	LAMP2/TAI	GO_CC
GO:00319ε	66	66/1164	393/2173'	9.90E-17	5.85E-15	3.136159	ENSG0000(	PSMB1/NA,	GO_CC
GO:19902(	35	35/1164	130/2173'	8.61E-16	4.40E-14	5.027723	ENSG0000(	NDUFA7/NI	GO_CC
GO:00057⁄	25	25/1164	66/21737	1.63E-15	6.24E-14	7.073636	ENSG0000(	NDUFA7/NI	GO_CC
GO:00309(	25	25/1164	66/21737	1.63E-15	6.24E-14	7.073636	ENSG0000(	NDUFA7/NI	GO_CC
GO:00057⁄	30	30/1164	104/2173'	1.29E-14	4.16E-13	5.386846	ENSG0000(	NDUFA7/NI	GO_CC
GO:00312:	50	50/1164	279/2173'	4.10E-14	1.14E-12	3.346666	ENSG0000(	TAP2/NA/I	GO_CC
GO:00443:	43	43/1164	216/2173'	5.86E-14	1.56E-12	3.717589	ENSG0000(	RPL38/MRI	GO_CC
GO:00988(	36	36/1164	158/2173'	9.19E-14	2.35E-12	4.254926	ENSG0000(	NDUFA7/NI	GO_CC
GO:00058⁄	50	50/1164	286/2173'	1.11E-13	2.73E-12	3.264755	ENSG0000(	RPL38/MRI	GO_CC
GO:00985!	31	31/1164	126/2173'	5.40E-13	1.07E-11	4.594495	ENSG0000(	HLA-DRA/I	GO_CC
GO:00444!	44	44/1164	255/2173'	5.30E-12	9.85E-11	3.222249	ENSG0000(	NDUFA7/NI	GO_CC
GO:00059:	62	62/1164	467/2173'	3.80E-11	6.29E-10	2.479256	ENSG0000(	SDCBP/RPI	GO_CC
GO:00306(	37	37/1164	207/2173'	9.10E-11	1.33E-09	3.337936	ENSG0000(	HLA-DRA/I	GO_CC
GO:00306(	41	41/1164	275/2173'	2.73E-09	3.56E-08	2.784183	ENSG0000(	HLA-DRA/I	GO_CC
GO:00197'	12	12/1164	27/21737	4.32E-09	5.52E-08	8.299733	ENSG0000(	PSMB1/NA,	GO_CC
GO:00306!	42	42/1164	306/2173'	2.10E-08	2.57E-07	2.563153	ENSG0000(	HLA-DRA/I	GO_CC
GO:00428:	11	11/1164	25/21737	2.20E-08	2.65E-07	8.216735	ENSG0000(	TAP2/NA/I	GO_CC
GO:00058⁄	18	18/1164	74/21737	4.80E-08	5.56E-07	4.542421	ENSG0000(	RPL38/VII	GO_CC
GO:00442:	10	10/1164	23/21737	1.13E-07	1.25E-06	8.119304	ENSG0000(	MBOAT7/N,	GO_CC
GO:00058:	12	12/1164	36/21737	1.99E-07	2.07E-06	6.2248	ENSG0000(	PSMB1/NA,	GO_CC
GO:00428:	8	8/1164	16/21737	5.78E-07	5.62E-06	9.337199	ENSG0000(	TAP2/NA/I	GO_CC
GO:00306(	42	42/1164	353/2173'	1.11E-06	1.05E-05	2.221883	ENSG0000(	LAMP2/BS'	GO_CC
GO:00725(	24	24/1164	150/2173'	1.45E-06	1.35E-05	2.987904	ENSG0000(	SDCBP/HSI	GO_CC
GO:00723!	7	7/1164	13/21737	1.60E-06	1.46E-05	10.05545	ENSG0000(	PPP1R10/I	GO_CC
GO:00442:	10	10/1164	30/21737	2.09E-06	1.86E-05	6.2248	ENSG0000(	MBOAT7/N,	GO_CC
GO:19053(	19	19/1164	106/2173'	3.19E-06	2.75E-05	3.347298	ENSG0000(	PSMB1/NA,	GO_CC
GO:00005(	16	16/1164	82/21737	6.07E-06	5.03E-05	3.643785	ENSG0000(	PSMB1/NA,	GO_CC
GO:00319(	35	35/1164	334/2173'	0.000121	0.000916	1.956898	ENSG0000(	SDCBP/AL(	GO_CC
GO:00018:	9	9/1164	39/21737	0.000173	0.001263	4.309477	ENSG0000(	AIF1/NA/I	GO_CC
GO:19053⁄	10	10/1164	48/21737	0.000189	0.001365	3.8905	ENSG0000(	TSEN34/N,	GO_CC
GO:00017:	22	22/1164	187/2173'	0.000447	0.003113	2.196988	ENSG0000(	EEF1A1/S	GO_CC
GO:00098:	40	40/1164	429/2173'	0.000486	0.003346	1.741203	ENSG0000(	HLA-A/NA,	GO_CC
GO:00452'	6	6/1164	21/21737	0.00063	0.004225	5.335542	ENSG0000(	COX8A/CO.	GO_CC
GO:00162:	14	14/1164	101/2173'	0.000967	0.006114	2.588531	ENSG0000(	GPX1/FBX(	GO_CC
GO:00003.	10	10/1164	59/21737	0.001066	0.006602	3.165152	ENSG0000(	MRPL33/MI	GO_CC
GO:00057(	10	10/1164	59/21737	0.001066	0.006602	3.165152	ENSG0000(	MRPL33/MI	GO_CC
GO:00082ε	10	10/1164	63/21737	0.001788	0.010393	2.96419	ENSG0000(	CTDNEP1/I	GO_CC
GO:19032:	10	10/1164	63/21737	0.001788	0.010393	2.96419	ENSG0000(	CTDNEP1/I	GO_CC
GO:00300:	22	22/1164	211/2173'	0.002201	0.012493	1.947094	ENSG0000(	TWF2/AIF.	GO_CC
GO:00319(	22	22/1164	211/2173'	0.002201	0.012493	1.947094	ENSG0000(	MCL1/PPP.	GO_CC
GO:00447:	23	23/1164	226/2173'	0.002422	0.013495	1.900492	ENSG0000(	JUNB/CEBI	GO_CC
GO:00056(	35	35/1164	400/2173'	0.00308	0.016276	1.63401	ENSG0000(	ABT1/PBX:	GO_CC
GO:00313(	24	24/1164	244/2173'	0.003084	0.016276	1.836826	ENSG0000(	LAMP2/ABI	GO_CC
GO:00082!	4	4/1164	13/21737	0.003962	0.019745	5.745969	ENSG0000(	TMEM258/I	GO_CC
GO:00308ε	15	15/1164	133/2173'	0.00509	0.024764	2.106135	ENSG0000(	ZNRD1/NA,	GO_CC
GO:00080:	20	20/1164	199/2173'	0.005154	0.024876	1.876824	ENSG0000(	CALM3/DGI	GO_CC
GO:00312!	36	36/1164	430/2173'	0.005509	0.02621	1.563438	ENSG0000(	EEF1A1/S	GO_CC
GO:00003.	12	12/1164	97/21737	0.005601	0.02621	2.310235	ENSG0000(	MRPL33/NI	GO_CC
GO:00057(	12	12/1164	97/21737	0.005601	0.02621	2.310235	ENSG0000(	MRPL33/NI	GO_CC
GO:00710.	12	12/1164	97/21737	0.005601	0.02621	2.310235	ENSG0000(	SRSF1/SNI	GO_CC
GO:00313(	21	21/1164	221/2173'	0.007942	0.035278	1.77449	ENSG0000(	LAMP2/SMI	GO_CC

GO:00985	31	31/1164	368/2173	0.008737	0.03853	1.573115	ENSG0000	LAMP2/SD	GO_CC
GO:00987	4	4/1164	16/21737	0.00887	0.03884	4.6686	ENSG0000	TOMM22/T	GO_CC
GO:00312	18	18/1164	183/2173	0.009529	0.041427	1.836826	ENSG0000	EEF1A1/A	GO_CC
GO:00312	18	18/1164	189/2173	0.013012	0.055009	1.778514	ENSG0000	BST2/ABH	GO_CC
GO:00056	20	20/1164	218/2173	0.013514	0.05674	1.713248	ENSG0000	SRSF1/SN	GO_CC
GO:00988	29	29/1164	355/2173	0.01608	0.066603	1.525514	ENSG0000	LAMP2/SD	GO_CC
GO:00008	3	3/1164	11/21737	0.018283	0.073251	5.093018	ENSG0000	CHMP2A/C	GO_CC
GO:00985	9	9/1164	79/21737	0.025064	0.09786	2.127463	ENSG0000	SMDT1/NA	GO_CC
GO:00300	3	3/1164	14/21737	0.035791	0.137126	4.001657	ENSG0000	TRAPPC5/	GO_CC
GO:00059	3	3/1164	15/21737	0.043004	0.158802	3.73488	ENSG0000	PRKAR1A/	GO_CC
GO:00331	3	3/1164	16/21737	0.05088	0.180288	3.50145	ENSG0000	ATP6V1B2	GO_CC
GO:00430	3	3/1164	16/21737	0.05088	0.180288	3.50145	ENSG0000	NCF4/NA/	GO_CC
GO:00226	4	4/1164	27/21737	0.053804	0.188469	2.766578	ENSG0000	PSME1/NA	GO_CC
GO:00308	4	4/1164	27/21737	0.053804	0.188469	2.766578	ENSG0000	ARL6IP1/	GO_CC
GO:00995	3	3/1164	18/21737	0.068542	0.237381	3.1124	ENSG0000	EIF4A3/D	GO_CC
GO:00059	24	24/1164	325/2173	0.07003	0.24117	1.379033	ENSG0000	EEF1A1/P	GO_CC
GO:00059	10	10/1164	110/2173	0.070529	0.241531	1.697673	ENSG0000	CLIC1/NA	GO_CC
GO:00350	3	3/1164	19/21737	0.078276	0.260779	2.948589	ENSG0000	PHF1/NA/	GO_CC
GO:00164	6	6/1164	57/21737	0.083387	0.274817	1.965726	ENSG0000	ATP6V1B2	GO_CC
GO:00709	3	3/1164	20/21737	0.088574	0.288807	2.80116	ENSG0000	EIF1/EIF	GO_CC
GO:00347	11	11/1164	130/2173	0.089326	0.289718	1.580141	ENSG0000	SNRPF/WD	GO_CC
GO:00432	12	12/1164	149/2173	0.103682	0.322624	1.503978	ENSG0000	AIF1/NA/	GO_CC
GO:00985	5	5/1164	48/21737	0.112636	0.34523	1.94525	ENSG0000	RAB11B/R	GO_CC
GO:00001	21	21/1164	298/2173	0.121752	0.365851	1.315981	ENSG0000	CBX7/GID	GO_CC
GO:00469	3	3/1164	23/21737	0.12253	0.366395	2.435791	ENSG0000	PPIF/BAK	GO_CC
GO:00017	4	4/1164	36/21737	0.124689	0.367471	2.074933	ENSG0000	LGALS3/C	GO_CC
GO:00432	5	5/1164	52/21737	0.144418	0.413649	1.795615	ENSG0000	CALM3/TU	GO_CC
GO:00985	14	14/1164	191/2173	0.145757	0.413649	1.368804	ENSG0000	EEF1A1/S	GO_CC
GO:00085	2	2/1164	13/21737	0.151357	0.413649	2.872984	ENSG0000	PSMC4/NA	GO_CC
GO:00988	2	2/1164	13/21737	0.151357	0.413649	2.872984	ENSG0000	ABHD17A/	GO_CC
GO:00989	2	2/1164	13/21737	0.151357	0.413649	2.872984	ENSG0000	RAB11B/R	GO_CC
GO:00016	9	9/1164	113/2173	0.151829	0.413649	1.487341	ENSG0000	CALR/NCF	GO_CC
GO:00098	12	12/1164	163/2173	0.164929	0.443426	1.374802	ENSG0000	S100A6/R	GO_CC
GO:00988	12	12/1164	163/2173	0.164929	0.443426	1.374802	ENSG0000	TWF2/CLI	GO_CC
GO:00319	8	8/1164	100/2173	0.167062	0.44506	1.493952	ENSG0000	TXNIP/CO	GO_CC
GO:00007	2	2/1164	14/21737	0.170618	0.44506	2.667771	ENSG0000	TINF2/NA	GO_CC
GO:00452	2	2/1164	14/21737	0.170618	0.44506	2.667771	ENSG0000	UQCR10/M	GO_CC
GO:00710	2	2/1164	14/21737	0.170618	0.44506	2.667771	ENSG0000	EIF4A3/S	GO_CC
GO:00431	13	13/1164	181/2173	0.173647	0.447389	1.341255	ENSG0000	ABHD17A/	GO_CC
GO:00056	3	3/1164	27/21737	0.173701	0.447389	2.074933	ENSG0000	TMEM109/	GO_CC
GO:00140	23	23/1164	349/2173	0.178663	0.458244	1.230691	ENSG0000	RPL38/RP	GO_CC
GO:00310	2	2/1164	15/21737	0.190251	0.474081	2.48992	ENSG0000	BLOC1S4/	GO_CC
GO:00312	2	2/1164	15/21737	0.190251	0.474081	2.48992	ENSG0000	LBR/TMEM	GO_CC
GO:00322	23	23/1164	353/2173	0.192868	0.478657	1.216746	ENSG0000	RPL38/RP	GO_CC
GO:00003	4	4/1164	43/21737	0.197012	0.486969	1.737153	ENSG0000	CDKN2D/C	GO_CC
GO:00425	2	2/1164	16/21737	0.210161	0.509205	2.3343	ENSG0000	POLE4/CH	GO_CC
GO:00444	2	2/1164	16/21737	0.210161	0.509205	2.3343	ENSG0000	LBR/TMEM	GO_CC
GO:00357	17	17/1164	260/2173	0.231354	0.53924	1.221018	ENSG0000	PATL1/HN	GO_CC
GO:00487	3	3/1164	32/21737	0.244104	0.564663	1.750725	ENSG0000	DGKI/STX	GO_CC
GO:00304	12	12/1164	178/2173	0.246312	0.565503	1.258948	ENSG0000	FLCN/JTB	GO_CC
GO:00312	24	24/1164	386/2173	0.253098	0.578915	1.161103	ENSG0000	EEF1A1/A	GO_CC
GO:00451	5	5/1164	64/21737	0.257662	0.587163	1.458937	ENSG0000	AAMP/UNC	GO_CC
GO:00312	5	5/1164	65/21737	0.267969	0.603916	1.436492	ENSG0000	SLC35A2/	GO_CC
GO:00995	23	23/1164	375/2173	0.280114	0.628974	1.145363	ENSG0000	RPL38/RP	GO_CC

GO:003028	3	3/1164	35/21737	0.288267	0.639315	1.600663	ENSG0000(	VAMP1/ST	GO_CC
GO:00058	2	2/1164	20/21737	0.290977	0.639315	1.86744	ENSG0000(	PIP5K1A/	GO_CC
GO:00347	2	2/1164	20/21737	0.290977	0.639315	1.86744	ENSG0000(	SNRPF/SN	GO_CC
GO:003068	6	6/1164	84/21737	0.294124	0.643922	1.333886	ENSG0000(	NGDN/EBN	GO_CC
GO:000569	2	2/1164	21/21737	0.311143	0.66923	1.778514	ENSG0000(	NOP10/SN	GO_CC
GO:003510	2	2/1164	21/21737	0.311143	0.66923	1.778514	ENSG0000(	CBX7/CBX	GO_CC
GO:004319	4	4/1164	53/21737	0.315425	0.674599	1.409389	ENSG0000(	SNCA/TNN	GO_CC
GO:00975	3	3/1164	37/21737	0.318041	0.674599	1.51414	ENSG0000(	PRKAR1A/	GO_CC
GO:001989	19	19/1164	315/2173'	0.329414	0.695259	1.126392	ENSG0000(	S100A6/D	GO_CC
GO:000583	2	2/1164	22/21737	0.331184	0.695259	1.697673	ENSG0000(	PSMC4/NA	GO_CC
GO:004525	2	2/1164	22/21737	0.331184	0.695259	1.697673	ENSG0000(	PPIF/MT-	GO_CC
GO:00436	8	8/1164	124/2173'	0.34613	0.715593	1.2048	ENSG0000(	DGKI/SRI	GO_CC
GO:00306	7	7/1164	107/2173'	0.349005	0.715593	1.22169	ENSG0000(	CALM3/RA	GO_CC
GO:004430	9	9/1164	143/2173'	0.358222	0.724721	1.175312	ENSG0000(	DGKI/SRI	GO_CC
GO:006202	25	25/1164	438/2173'	0.400071	0.784175	1.06589	ENSG0000(	CSTB/S10	GO_CC
GO:00001	4	4/1164	60/21737	0.401682	0.784175	1.24496	ENSG0000(	SIN3B/DN	GO_CC
GO:004429	5	5/1164	79/21737	0.417342	0.797519	1.181924	ENSG0000(	FLCN/RAP	GO_CC
GO:009889	1	1/1164	10/21737	0.423327	0.797519	1.86744	ENSG0000(	DGKI	GO_CC
GO:199072	1	1/1164	10/21737	0.423327	0.797519	1.86744	ENSG0000(	LSM7	GO_CC
GO:190249	20	20/1164	354/2173'	0.435212	0.811773	1.055051	ENSG0000(	CALM3/SM	GO_CC
GO:00059	2	2/1164	28/21737	0.446656	0.811773	1.333886	ENSG0000(	CISH/SOC	GO_CC
GO:003089	2	2/1164	28/21737	0.446656	0.811773	1.333886	ENSG0000(	PURA/PCN	GO_CC
GO:00312	6	6/1164	100/2173'	0.447864	0.811773	1.120464	ENSG0000(	POLE4/MS	GO_CC
GO:000023	1	1/1164	11/21737	0.454222	0.811773	1.697673	ENSG0000(	MAPRE1	GO_CC
GO:00021	1	1/1164	11/21737	0.454222	0.811773	1.697673	ENSG0000(	PLXND1	GO_CC
GO:003080	1	1/1164	11/21737	0.454222	0.811773	1.697673	ENSG0000(	PGRMC1	GO_CC
GO:006182	1	1/1164	11/21737	0.454222	0.811773	1.697673	ENSG0000(	ALDOA	GO_CC
GO:001989	10	10/1164	174/2173'	0.455628	0.811919	1.073241	ENSG0000(	S100A6/D	GO_CC
GO:000573	2	2/1164	29/21737	0.464838	0.822402	1.28789	ENSG0000(	SNRPF/NO	GO_CC
GO:003326	22	22/1164	397/2173'	0.465536	0.822402	1.034853	ENSG0000(	TWF2/DYN	GO_CC
GO:003120	3	3/1164	48/21737	0.478215	0.82323	1.16715	ENSG0000(	STX4/STX	GO_CC
GO:003005	2	2/1164	30/21737	0.482662	0.82323	1.24496	ENSG0000(	JUP/DSC2	GO_CC
GO:00452	1	1/1164	12/21737	0.483463	0.82323	1.5562	ENSG0000(	KAT2A	GO_CC
GO:00991	1	1/1164	12/21737	0.483463	0.82323	1.5562	ENSG0000(	ABHD17A	GO_CC
GO:01201	1	1/1164	12/21737	0.483463	0.82323	1.5562	ENSG0000(	LSM7	GO_CC
GO:003042	10	10/1164	180/2173'	0.498744	0.838441	1.037467	ENSG0000(	TWF2/DYN	GO_CC
GO:003053	7	7/1164	125/2173'	0.506971	0.838441	1.045766	ENSG0000(	SNRPF/LS	GO_CC
GO:004485	7	7/1164	125/2173'	0.506971	0.838441	1.045766	ENSG0000(	ITGB2/SL	GO_CC
GO:003228	1	1/1164	13/21737	0.511138	0.838441	1.436492	ENSG0000(	NTSR1	GO_CC
GO:004262	1	1/1164	13/21737	0.511138	0.838441	1.436492	ENSG0000(	APOBR	GO_CC
GO:004526	1	1/1164	13/21737	0.511138	0.838441	1.436492	ENSG0000(	MT-ATP6	GO_CC
GO:01201	5	5/1164	88/21737	0.511545	0.838441	1.061045	ENSG0000(	BLOC1S4/	GO_CC
GO:009722	11	11/1164	201/2173'	0.514556	0.841128	1.021982	ENSG0000(	GABARAP/	GO_CC
GO:00313	3	3/1164	52/21737	0.532249	0.844576	1.077369	ENSG0000(	CCDC115/	GO_CC
GO:00320	1	1/1164	14/21737	0.537332	0.844576	1.333886	ENSG0000(	DGKI	GO_CC
GO:00336	1	1/1164	14/21737	0.537332	0.844576	1.333886	ENSG0000(	GBP2	GO_CC
GO:004365	1	1/1164	14/21737	0.537332	0.844576	1.333886	ENSG0000(	GBP2	GO_CC
GO:00992	1	1/1164	14/21737	0.537332	0.844576	1.333886	ENSG0000(	DGKI	GO_CC
GO:009952	1	1/1164	14/21737	0.537332	0.844576	1.333886	ENSG0000(	HSPA8	GO_CC
GO:003042	10	10/1164	186/2173'	0.54091	0.848025	1.004	ENSG0000(	TWF2/DYN	GO_CC
GO:000153	4	4/1164	72/21737	0.542857	0.848906	1.037467	ENSG0000(	CSTA/JUP	GO_CC
GO:003158	1	1/1164	15/21737	0.562124	0.862897	1.24496	ENSG0000(	PRKAR1A	GO_CC
GO:003820	1	1/1164	15/21737	0.562124	0.862897	1.24496	ENSG0000(	AKT1S1	GO_CC
GO:000592	2	2/1164	35/21737	0.566	0.862897	1.067108	ENSG0000(	PANX2/DB	GO_CC

GO:003123	5	5/1164	94/21737	0.570739	0.862897	0.993319	ENSG0000(	S100A6/RI	GO_CC
GO:00316'	8	8/1164	152/2173'	0.571185	0.862897	0.982863	ENSG0000(	SRI/SLC4/	GO_CC
GO:004329	8	8/1164	152/2173'	0.571185	0.862897	0.982863	ENSG0000(	RHOA/RAP:	GO_CC
GO:004365	4	4/1164	75/21737	0.575474	0.862897	0.995968	ENSG0000(	DYNLT1/II	GO_CC
GO:003364	1	1/1164	16/21737	0.585588	0.862897	1.16715	ENSG0000(	GBP2	GO_CC
GO:003215	3	3/1164	57/21737	0.595106	0.87482	0.982863	ENSG0000(	RHOB/RHO/	GO_CC
GO:003404	1	1/1164	17/21737	0.607796	0.878723	1.098494	ENSG0000(	RAB1B	GO_CC
GO:009855	1	1/1164	17/21737	0.607796	0.878723	1.098494	ENSG0000(	RPL27	GO_CC
GO:003001	11	11/1164	216/2173'	0.611482	0.881895	0.951011	ENSG0000(	CALM3/SR	GO_CC
GO:003301	2	2/1164	39/21737	0.62539	0.881895	0.957661	ENSG0000(	SRI/TMEM	GO_CC
GO:000590	4	4/1164	80/21737	0.626769	0.881895	0.93372	ENSG0000(	ARRB2/CT'	GO_CC
GO:000016	1	1/1164	18/21737	0.628814	0.881895	1.037467	ENSG0000(	PPP1R15A	GO_CC
GO:004850	1	1/1164	18/21737	0.628814	0.881895	1.037467	ENSG0000(	SRP68	GO_CC
GO:003001	7	7/1164	141/2173'	0.635178	0.881895	0.927098	ENSG0000(	SRI/SLC4/	GO_CC
GO:00316'	2	2/1164	40/21737	0.639215	0.881895	0.93372	ENSG0000(	MYL4/ALD(	GO_CC
GO:001633	3	3/1164	61/21737	0.641255	0.881895	0.918413	ENSG0000(	JUP/DVL2/	GO_CC
GO:000014	1	1/1164	19/21737	0.648707	0.881895	0.982863	ENSG0000(	TNFAIP2	GO_CC
GO:001633	1	1/1164	19/21737	0.648707	0.881895	0.982863	ENSG0000(	JUP	GO_CC
GO:009719	1	1/1164	19/21737	0.648707	0.881895	0.982863	ENSG0000(	CD81	GO_CC
GO:004421	4	4/1164	83/21737	0.655578	0.881895	0.899971	ENSG0000(	DYNLT1/II	GO_CC
GO:000193	1	1/1164	20/21737	0.667535	0.881895	0.93372	ENSG0000(	MSN	GO_CC
GO:003125	1	1/1164	20/21737	0.667535	0.881895	0.93372	ENSG0000(	MSN	GO_CC
GO:003430	1	1/1164	20/21737	0.667535	0.881895	0.93372	ENSG0000(	APOBR	GO_CC
GO:190271	1	1/1164	20/21737	0.667535	0.881895	0.93372	ENSG0000(	GABRG1	GO_CC
GO:003435	2	2/1164	44/21737	0.690507	0.889245	0.848836	ENSG0000(	APOBR/HPI	GO_CC
GO:19907'	2	2/1164	44/21737	0.690507	0.889245	0.848836	ENSG0000(	APOBR/HPI	GO_CC
GO:000593	6	6/1164	130/2173'	0.702068	0.896287	0.861895	ENSG0000(	MAP1LC3B/	GO_CC
GO:003190	3	3/1164	67/21737	0.703286	0.896287	0.836167	ENSG0000(	ARF1/HMG(	GO_CC
GO:009701	6	6/1164	131/2173'	0.709025	0.898446	0.855316	ENSG0000(	MAP1LC3B/	GO_CC
GO:003215	3	3/1164	68/21737	0.712785	0.898446	0.823871	ENSG0000(	RHOB/RHO/	GO_CC
GO:004444	11	11/1164	234/2173'	0.713773	0.898446	0.877856	ENSG0000(	CALM3/SR	GO_CC
GO:000208	1	1/1164	23/21737	0.718182	0.898461	0.81193	ENSG0000(	BSG	GO_CC
GO:00331'	1	1/1164	23/21737	0.718182	0.898461	0.81193	ENSG0000(	MT-ATP6	GO_CC
GO:007016	6	6/1164	137/2173'	0.74842	0.923102	0.817857	ENSG0000(	RAP2B/DD	GO_CC
GO:003215	3	3/1164	73/21737	0.756777	0.927567	0.767441	ENSG0000(	RHOB/RHO/	GO_CC
GO:003001	1	1/1164	26/21737	0.761119	0.927567	0.718246	ENSG0000(	PATL1	GO_CC
GO:003438	1	1/1164	26/21737	0.761119	0.927567	0.718246	ENSG0000(	APOBR	GO_CC
GO:009722	1	1/1164	26/21737	0.761119	0.927567	0.718246	ENSG0000(	GABARAP	GO_CC
GO:003280	1	1/1164	27/21737	0.773927	0.935734	0.691644	ENSG0000(	KCNE3	GO_CC
GO:004429	1	1/1164	28/21737	0.786048	0.944057	0.666943	ENSG0000(	KCNE3	GO_CC
GO:003612	4	4/1164	100/2173'	0.789759	0.944057	0.746976	ENSG0000(	GABARAP/	GO_CC
GO:003031	2	2/1164	54/21737	0.79273	0.944057	0.691644	ENSG0000(	SRI/BIN1	GO_CC
GO:003242	2	2/1164	54/21737	0.79273	0.944057	0.691644	ENSG0000(	TWF2/MPP	GO_CC
GO:003143	1	1/1164	29/21737	0.797519	0.944057	0.643945	ENSG0000(	ALDOA	GO_CC
GO:004878	3	3/1164	80/21737	0.809039	0.950078	0.70029	ENSG0000(	DGKI/STX	GO_CC
GO:000583	1	1/1164	31/21737	0.818652	0.950395	0.6024	ENSG0000(	GNAI2	GO_CC
GO:000830	1	1/1164	31/21737	0.818652	0.950395	0.6024	ENSG0000(	ITGB2	GO_CC
GO:009050	1	1/1164	31/21737	0.818652	0.950395	0.6024	ENSG0000(	MTA2	GO_CC
GO:190530	1	1/1164	31/21737	0.818652	0.950395	0.6024	ENSG0000(	GNAI2	GO_CC
GO:003242	2	2/1164	59/21737	0.831696	0.953038	0.63303	ENSG0000(	TWF2/MPP	GO_CC
GO:003430	1	1/1164	33/21737	0.837581	0.957778	0.565891	ENSG0000(	HPR	GO_CC
GO:009880	8	8/1164	200/2173'	0.845528	0.957778	0.746976	ENSG0000(	SDCBP/B2I	GO_CC
GO:004443	4	4/1164	110/2173'	0.846845	0.957778	0.679069	ENSG0000(	ARF1/HMG(	GO_CC
GO:004319	1	1/1164	40/21737	0.889582	0.993286	0.46686	ENSG0000(	NTSR1	GO_CC

GO:00301	3	3/1164	100/2173	0.908874	0.999884	0.560232	ENSG0000	CHMP2A/A	GO_CC
GO:00427	6	6/1164	175/2173	0.911995	0.999884	0.640265	ENSG0000	DGKI/APH	GO_CC
GO:00975	1	1/1164	45/21737	0.916191	0.999884	0.414987	ENSG0000	ULK3	GO_CC
GO:00988	2	2/1164	76/21737	0.919502	0.999884	0.491432	ENSG0000	ABHD17A/	GO_CC
GO:19049	4	4/1164	131/2173	0.925203	0.999884	0.570211	ENSG0000	ZNHIT1/I	GO_CC
GO:00301	1	1/1164	48/21737	0.928972	0.999884	0.38905	ENSG0000	AP5B1	GO_CC
GO:00360	4	4/1164	139/2173	0.943951	0.999884	0.537393	ENSG0000	RILP/NA/	GO_CC
GO:00302	1	1/1164	54/21737	0.948988	0.999884	0.345822	ENSG0000	DYNLT1	GO_CC
GO:00991	1	1/1164	54/21737	0.948988	0.999884	0.345822	ENSG0000	ABHD17A	GO_CC
GO:00163	8	8/1164	244/2173	0.953116	0.999884	0.612275	ENSG0000	AQP3/ARR	GO_CC
GO:00423	4	4/1164	147/2173	0.95831	0.999884	0.508147	ENSG0000	SRI/SLC2	GO_CC
GO:00325	1	1/1164	60/21737	0.963367	0.999884	0.31124	ENSG0000	GABRG1	GO_CC
GO:00163	12	12/1164	354/2173	0.969141	0.999884	0.63303	ENSG0000	BST2/ATP	GO_CC
GO:00452	12	12/1164	357/2173	0.971516	0.999884	0.627711	ENSG0000	ABHD17A/	GO_CC
GO:00059	3	3/1164	129/2173	0.971663	0.999884	0.434288	ENSG0000	S100A11/	GO_CC
GO:00358	1	1/1164	67/21737	0.975108	0.999884	0.278722	ENSG0000	UNC119B	GO_CC
GO:00315	5	5/1164	191/2173	0.977903	0.999884	0.488859	ENSG0000	GABARAP/	GO_CC
GO:00996	2	2/1164	105/2173	0.978748	0.999884	0.355703	ENSG0000	ABHD17A/	GO_CC
GO:00970	16	16/1164	470/2173	0.982716	0.999884	0.635724	ENSG0000	ABHD17A/	GO_CC
GO:00451	14	14/1164	426/2173	0.984055	0.999884	0.613713	ENSG0000	BST2/ATP	GO_CC
GO:00989	1	1/1164	80/21737	0.987859	0.999884	0.23343	ENSG0000	ABHD17A	GO_CC
GO:00988	1	1/1164	84/21737	0.990266	0.999884	0.222314	ENSG0000	APH1A	GO_CC
GO:00601	1	1/1164	101/2173	0.996197	0.999884	0.184895	ENSG0000	BBS5	GO_CC
GO:00007	1	1/1164	109/2173	0.997557	0.999884	0.171325	ENSG0000	IRF4	GO_CC
GO:00007	1	1/1164	114/2173	0.998147	0.999884	0.163811	ENSG0000	DCTN3	GO_CC
GO:00992	2	2/1164	176/2173	0.999341	0.999884	0.212209	ENSG0000	ABHD17A/	GO_CC
GO:00989	1	1/1164	134/2173	0.999388	0.999884	0.139361	ENSG0000	ABHD17A	GO_CC
GO:00444	11	11/1164	495/2173	0.999852	0.999884	0.414987	ENSG0000	RILP/NA/	GO_CC
GO:00996	1	1/1164	164/2173	0.999884	0.999884	0.113868	ENSG0000	APH1A	GO_CC
GO:00308	16	16/1121	25/19728	1.34E-14	2.39E-12	11.26309	ENSG0000	HLA-A/NA	GO_MF
GO:00230	24	24/1121	70/19728	2.86E-13	4.26E-11	6.033796	ENSG0000	HLA-DRA/	GO_MF
GO:00469	15	15/1121	28/19728	3.53E-12	2.63E-10	9.427807	ENSG0000	TAP2/NA/	GO_MF
GO:00426	29	29/1121	118/1972	1.29E-11	8.25E-10	4.325073	ENSG0000	HLA-DRA/	GO_MF
GO:00166	29	29/1121	127/1972	8.87E-11	4.67E-09	4.018572	ENSG0000	NDUFA7/G	GO_MF
GO:00422	50	50/1121	409/1972	2.73E-07	9.77E-06	2.151415	ENSG0000	HLA-DRA/	GO_MF
GO:00428	15	15/1121	68/19728	4.89E-06	0.000118	3.882038	ENSG0000	MCL1/TAP	GO_MF
GO:00718	10	10/1121	35/19728	1.68E-05	0.000349	5.028164	ENSG0000	IRS2/ARR	GO_MF
GO:00441	9	9/1121	35/19728	0.00011	0.001971	4.525347	ENSG0000	HSPA6/CA	GO_MF
GO:00150	8	8/1121	28/19728	0.000119	0.002001	5.028164	ENSG0000	COX8A/CO	GO_MF
GO:00166	8	8/1121	29/19728	0.000156	0.002531	4.854779	ENSG0000	COX8A/CO	GO_MF
GO:00977	11	11/1121	56/19728	0.000267	0.004181	3.456862	ENSG0000	ZNRD1/NA	GO_MF
GO:00090	17	17/1121	116/1972	0.000294	0.004536	2.579101	ENSG0000	COX8A/UQ	GO_MF
GO:00517	9	9/1121	45/19728	0.000829	0.012153	3.519715	ENSG0000	HSPA6/HS	GO_MF
GO:00037	14	14/1121	100/1972	0.001537	0.021468	2.4638	ENSG0000	DHX58/EI	GO_MF
GO:00198	8	8/1121	40/19728	0.0016	0.022012	3.519715	ENSG0000	FCGR3B/L	GO_MF
GO:00167	16	16/1121	127/1972	0.002272	0.029432	2.217143	ENSG0000	LYZ/PSAP	GO_MF
GO:00055	7	7/1121	35/19728	0.003108	0.039693	3.519715	ENSG0000	S100A8/A	GO_MF
GO:00302	7	7/1121	36/19728	0.003672	0.044972	3.421945	ENSG0000	HLA-DRA/	GO_MF
GO:00048	38	38/1121	429/1972	0.004569	0.054465	1.558848	ENSG0000	CSTB/BST	GO_MF
GO:00162	4	4/1121	13/19728	0.004903	0.057674	5.414945	ENSG0000	APRT/CYB	GO_MF
GO:00045	15	15/1121	127/1972	0.005657	0.064834	2.078572	ENSG0000	RNASE2/Z	GO_MF
GO:00192	14	14/1121	116/1972	0.006096	0.068986	2.123966	ENSG0000	CALM3/PP	GO_MF
GO:00510	17	17/1121	153/1972	0.006237	0.069695	1.955397	ENSG0000	AAMP/HSP	GO_MF
GO:00083	5	5/1121	22/19728	0.006872	0.074019	3.999676	ENSG0000	MCL1/SEC	GO_MF

GO:00166(	9	9/1121	62/19728	0.008022	0.085382	2.554632	ENSG0000(	IFI30/SR]	GO_MF
GO:00046(	5	5/1121	24/19728	0.010096	0.104955	3.666369	ENSG0000(	MGST3/AL(	GO_MF
GO:00510{	13	13/1121	112/1972{	0.011035	0.112106	2.042691	ENSG0000(	AHSA1/CA]	GO_MF
GO:00228{	5	5/1121	25/19728	0.012042	0.11962	3.519715	ENSG0000(	MCL1/SEC(	GO_MF
GO:00474{	14	14/1121	129/1972{	0.014947	0.139197	1.909923	ENSG0000(	SDCBP/CA]	GO_MF
GO:00166'	3	3/1121	10/19728	0.016248	0.145301	5.279572	ENSG0000(	UQCR10/U(	GO_MF
GO:01401(	5	5/1121	28/19728	0.019362	0.168058	3.142602	ENSG0000(	B3GALT6/]	GO_MF
GO:00167:	4	4/1121	19/19728	0.020259	0.172494	3.704963	ENSG0000(	FTL/FTH1,	GO_MF
GO:00510]	20	20/1121	215/1972{	0.020902	0.173225	1.637077	ENSG0000(	TWF2/AIF	GO_MF
GO:00166{	8	8/1121	61/19728	0.021532	0.173225	2.30801	ENSG0000(	MGST3/AL(	GO_MF
GO:00310'	15	15/1121	149/1972{	0.022512	0.175003	1.771668	ENSG0000(	HSPA6/IT(	GO_MF
GO:00985:	7	7/1121	52/19728	0.026903	0.202113	2.369039	ENSG0000(	RXRB/NA/]	GO_MF
GO:00055:	3	3/1121	12/19728	0.027364	0.202176	4.399643	ENSG0000(	DBN1/CTT]	GO_MF
GO:00381{	4	4/1121	21/19728	0.0286	0.209578	3.352109	ENSG0000(	TLR2/FCN	GO_MF
GO:00551(	4	4/1121	22/19728	0.033429	0.237183	3.19974	ENSG0000(	RPL11/RP]	GO_MF
GO:00167<	25	25/1121	300/1972{	0.035983	0.249372	1.466548	ENSG0000(	WDR5/SAT	GO_MF
GO:19015(	6	6/1121	47/19728	0.048876	0.32367	2.246626	ENSG0000(	SLC25A5/{	GO_MF
GO:00037(	7	7/1121	61/19728	0.056866	0.360139	2.019508	ENSG0000(	RXRB/NA/]	GO_MF
GO:00168<	3	3/1121	16/19728	0.058878	0.360529	3.299732	ENSG0000(	MGST3/AL(	GO_MF
GO:00084'	3	3/1121	17/19728	0.068578	0.400711	3.10563	ENSG0000(	ABHD17A/]	GO_MF
GO:00985!	3	3/1121	17/19728	0.068578	0.400711	3.10563	ENSG0000(	ABHD17A/]	GO_MF
GO:00483(	7	7/1121	64/19728	0.070159	0.407289	1.924844	ENSG0000(	S100A6/C/	GO_MF
GO:00976(	3	3/1121	18/19728	0.078948	0.435674	2.933095	ENSG0000(	SKP1/DDB	GO_MF
GO:00197(	4	4/1121	30/19728	0.087957	0.474146	2.346476	ENSG0000(	ZDHHC7/Z]	GO_MF
GO:00445<	3	3/1121	20/19728	0.101553	0.523842	2.639786	ENSG0000(	S100A6/S	GO_MF
GO:00037]	26	26/1121	352/1972{	0.103742	0.523842	1.299895	ENSG0000(	ABT1/JUN]	GO_MF
GO:00055]	8	8/1121	84/19728	0.104104	0.523842	1.676055	ENSG0000(	MMP9/DDR	GO_MF
GO:00055:	2	2/1121	10/19728	0.107229	0.523842	3.519715	ENSG0000(	FKBP2/NA	GO_MF
GO:00317:	2	2/1121	10/19728	0.107229	0.523842	3.519715	ENSG0000(	HBD/HBB	GO_MF
GO:00717:	2	2/1121	10/19728	0.107229	0.523842	3.519715	ENSG0000(	TLR2/CD1]	GO_MF
GO:00228(	29	29/1121	401/1972{	0.10906	0.529891	1.272715	ENSG0000(	TAP2/NA/]	GO_MF
GO:00159:	5	5/1121	45/19728	0.110415	0.533572	1.955397	ENSG0000(	SLC25A5/{	GO_MF
GO:00051'	12	12/1121	144/1972{	0.118348	0.558231	1.466548	ENSG0000(	ISG15/IT(	GO_MF
GO:00046!	2	2/1121	11/19728	0.1263	0.558231	3.19974	ENSG0000(	PRKACA/N/	GO_MF
GO:00430:	13	13/1121	161/1972{	0.127978	0.560847	1.421003	ENSG0000(	PIM1/EIF	GO_MF
GO:00037]	19	19/1121	257/1972{	0.145711	0.623278	1.301062	ENSG0000(	JUNB/DRA]	GO_MF
GO:00314!	8	8/1121	92/19728	0.151748	0.646012	1.530311	ENSG0000(	ZNHIT1/H]	GO_MF
GO:00039(	2	2/1121	13/19728	0.166448	0.678121	2.707473	ENSG0000(	HMGA1/AP]	GO_MF
GO:00199!	2	2/1121	13/19728	0.166448	0.678121	2.707473	ENSG0000(	CDIPT/RA{	GO_MF
GO:00015:	4	4/1121	38/19728	0.167634	0.678121	1.852481	ENSG0000(	PSMB4/TL]	GO_MF
GO:00431'	16	16/1121	217/1972{	0.173119	0.697155	1.297591	ENSG0000(	S100A8/P{	GO_MF
GO:00429]	2	2/1121	14/19728	0.187254	0.725043	2.514082	ENSG0000(	RALBP1/S]	GO_MF
GO:00019(	3	3/1121	28/19728	0.211019	0.76377	1.885561	ENSG0000(	RGS2/RGS	GO_MF
GO:00436:	5	5/1121	59/19728	0.24324	0.842854	1.491404	ENSG0000(	ADAM8/MY]	GO_MF
GO:00055:	2	2/1121	17/19728	0.251236	0.852151	2.07042	ENSG0000(	LBR/TMEM:	GO_MF
GO:00480:	2	2/1121	17/19728	0.251236	0.852151	2.07042	ENSG0000(	MT-ND4/A(	GO_MF
GO:00054{	3	3/1121	31/19728	0.257057	0.863945	1.703088	ENSG0000(	STX4/STX:	GO_MF
GO:00435<	3	3/1121	31/19728	0.257057	0.863945	1.703088	ENSG0000(	IRS2/CAL]	GO_MF
GO:00200:	11	11/1121	154/1972{	0.260003	0.870573	1.257041	ENSG0000(	SDHC/CYP<	GO_MF
GO:00192]	4	4/1121	46/19728	0.264329	0.881753	1.530311	ENSG0000(	YDJC/SIN:	GO_MF
GO:00480:	6	6/1121	77/19728	0.272537	0.883545	1.371317	ENSG0000(	TALDO1/C]	GO_MF
GO:00018<	2	2/1121	18/19728	0.272772	0.883545	1.955397	ENSG0000(	ITGB2/CA]	GO_MF
GO:00700(	2	2/1121	18/19728	0.272772	0.883545	1.955397	ENSG0000(	PFN1/RAB/	GO_MF
GO:00305<	4	4/1121	47/19728	0.27713	0.891204	1.497751	ENSG0000(	LY6E/NA/	GO_MF

GO:00150[illegible]	4	4/1121	49/19728	0.303007	0.95863	1.436618	ENSG0000[illegible]	LILRA4/N[illegible]	GO_MF
GO:00018[illegible]	3	3/1121	34/19728	0.304263	0.95863	1.552815	ENSG0000[illegible]	ITGB2/CA[illegible]	GO_MF
GO:00986[illegible]	4	4/1121	50/19728	0.316046	0.977664	1.407886	ENSG0000[illegible]	S100A11/[illegible]	GO_MF
GO:00469[illegible]	11	11/1121	164/1972[illegible]	0.329538	0.990918	1.180392	ENSG0000[illegible]	SDHC/CYP[illegible]	GO_MF
GO:00156[illegible]	3	3/1121	36/19728	0.335958	0.990918	1.466548	ENSG0000[illegible]	SLC25A5/[illegible]	GO_MF
GO:00167[illegible]	2	2/1121	21/19728	0.336956	0.990918	1.676055	ENSG0000[illegible]	QPCT/EPB[illegible]	GO_MF
GO:00518[illegible]	2	2/1121	21/19728	0.336956	0.990918	1.676055	ENSG0000[illegible]	PSAP/MAN[illegible]	GO_MF
GO:00996[illegible]	2	2/1121	21/19728	0.336956	0.990918	1.676055	ENSG0000[illegible]	LY6E/NA	GO_MF
GO:00307[illegible]	2	2/1121	22/19728	0.358013	1	1.59987	ENSG0000[illegible]	RAB5B/HP[illegible]	GO_MF
GO:00605[illegible]	24	24/1121	390/1972[illegible]	0.372849	1	1.082989	ENSG0000[illegible]	RGS2/CDC[illegible]	GO_MF
GO:00466[illegible]	2	2/1121	23/19728	0.37882	1	1.530311	ENSG0000[illegible]	PSAP/S1P[illegible]	GO_MF
GO:00511[illegible]	2	2/1121	23/19728	0.37882	1	1.530311	ENSG0000[illegible]	SLC48A1/[illegible]	GO_MF
GO:00001[illegible]	8	8/1121	121/1972[illegible]	0.382072	1	1.163542	ENSG0000[illegible]	GABARAPL[illegible]	GO_MF
GO:01401[illegible]	10	10/1121	158/1972[illegible]	0.409526	1	1.113834	ENSG0000[illegible]	TSEN34/N[illegible]	GO_MF
GO:00192[illegible]	14	14/1121	227/1972[illegible]	0.415361	1	1.085375	ENSG0000[illegible]	CDKN2D/C[illegible]	GO_MF
GO:19908[illegible]	4	4/1121	58/19728	0.420678	1	1.213695	ENSG0000[illegible]	NFE2L1/Z[illegible]	GO_MF
GO:00443[illegible]	8	8/1121	127/1972[illegible]	0.434501	1	1.108572	ENSG0000[illegible]	CALM3/SR[illegible]	GO_MF
GO:00037[illegible]	1	1/1121	10/19728	0.442977	1	1.759857	ENSG0000[illegible]	LYZ	GO_MF
GO:00452[illegible]	1	1/1121	10/19728	0.442977	1	1.759857	ENSG0000[illegible]	JUP	GO_MF
GO:00037[illegible]	3	3/1121	43/19728	0.445041	1	1.227807	ENSG0000[illegible]	PPIF/FKB[illegible]	GO_MF
GO:00040[illegible]	1	1/1121	11/19728	0.474644	1	1.59987	ENSG0000[illegible]	ADCY7	GO_MF
GO:00324[illegible]	1	1/1121	11/19728	0.474644	1	1.59987	ENSG0000[illegible]	PCNA	GO_MF
GO:00432[illegible]	1	1/1121	11/19728	0.474644	1	1.59987	ENSG0000[illegible]	PTGES2	GO_MF
GO:00435[illegible]	1	1/1121	11/19728	0.474644	1	1.59987	ENSG0000[illegible]	PRKCD	GO_MF
GO:00166[illegible]	2	2/1121	29/19728	0.496542	1	1.213695	ENSG0000[illegible]	PYCR2/AA[illegible]	GO_MF
GO:00168[illegible]	3	3/1121	47/19728	0.504193	1	1.123313	ENSG0000[illegible]	PPIF/FKB[illegible]	GO_MF
GO:00055[illegible]	1	1/1121	12/19728	0.504513	1	1.466548	ENSG0000[illegible]	FOLR3	GO_MF
GO:00152[illegible]	1	1/1121	12/19728	0.504513	1	1.466548	ENSG0000[illegible]	SLC6A8	GO_MF
GO:00315[illegible]	1	1/1121	12/19728	0.504513	1	1.466548	ENSG0000[illegible]	P4HB	GO_MF
GO:00336[illegible]	1	1/1121	12/19728	0.504513	1	1.466548	ENSG0000[illegible]	FCN1	GO_MF
GO:00335[illegible]	2	2/1121	30/19728	0.514757	1	1.173238	ENSG0000[illegible]	SIN3B/MT[illegible]	GO_MF
GO:00054[illegible]	6	6/1121	101/1972[illegible]	0.515474	1	1.04546	ENSG0000[illegible]	TSPO/CD8[illegible]	GO_MF
GO:00434[illegible]	1	1/1121	13/19728	0.532685	1	1.353736	ENSG0000[illegible]	SLC4A1	GO_MF
GO:00512[illegible]	5	5/1121	85/19728	0.533894	1	1.03521	ENSG0000[illegible]	SH3BP2/F[illegible]	GO_MF
GO:00167[illegible]	17	17/1121	299/1972[illegible]	0.53481	1	1.000588	ENSG0000[illegible]	SDF2/DPM[illegible]	GO_MF
GO:00519[illegible]	2	2/1121	32/19728	0.549851	1	1.099911	ENSG0000[illegible]	RILP/NA	GO_MF
GO:00314[illegible]	7	7/1121	123/1972[illegible]	0.55346	1	1.001545	ENSG0000[illegible]	KDM6B/WB[illegible]	GO_MF
GO:00053[illegible]	1	1/1121	14/19728	0.559257	1	1.257041	ENSG0000[illegible]	SLC25A22	GO_MF
GO:00168[illegible]	1	1/1121	14/19728	0.559257	1	1.257041	ENSG0000[illegible]	APMAP	GO_MF
GO:00191[illegible]	1	1/1121	14/19728	0.559257	1	1.257041	ENSG0000[illegible]	PCNA	GO_MF
GO:00970[illegible]	1	1/1121	14/19728	0.559257	1	1.257041	ENSG0000[illegible]	PSAP	GO_MF
GO:00168[illegible]	3	3/1121	51/19728	0.559916	1	1.03521	ENSG0000[illegible]	PTGES2/P[illegible]	GO_MF
GO:00192[illegible]	2	2/1121	33/19728	0.566715	1	1.06658	ENSG0000[illegible]	AMPD2/ZB[illegible]	GO_MF
GO:00199[illegible]	8	8/1121	143/1972[illegible]	0.569527	1	0.984536	ENSG0000[illegible]	IL13RA1/[illegible]	GO_MF
GO:00340[illegible]	2	2/1121	34/19728	0.583118	1	1.03521	ENSG0000[illegible]	POLE4/CH[illegible]	GO_MF
GO:00165[illegible]	1	1/1121	15/19728	0.584319	1	1.173238	ENSG0000[illegible]	ALAS2	GO_MF
GO:00510[illegible]	3	3/1121	53/19728	0.586315	1	0.996146	ENSG0000[illegible]	PRKAR1A/[illegible]	GO_MF
GO:00167[illegible]	4	4/1121	72/19728	0.590627	1	0.977698	ENSG0000[illegible]	SULT1A1/[illegible]	GO_MF
GO:00037[illegible]	1	1/1121	16/19728	0.607957	1	1.099911	ENSG0000[illegible]	P4HB	GO_MF
GO:00723[illegible]	1	1/1121	16/19728	0.607957	1	1.099911	ENSG0000[illegible]	SLC6A8	GO_MF
GO:00168[illegible]	3	3/1121	56/19728	0.623962	1	0.942781	ENSG0000[illegible]	ODC1/ALD[illegible]	GO_MF
GO:00198[illegible]	2	2/1121	37/19728	0.629549	1	0.951274	ENSG0000[illegible]	HBD/HBB	GO_MF
GO:00009[illegible]	1	1/1121	17/19728	0.630252	1	1.03521	ENSG0000[illegible]	PURA	GO_MF
GO:00192[illegible]	1	1/1121	17/19728	0.630252	1	1.03521	ENSG0000[illegible]	VIM	GO_MF

GO:00050{	8	8/1121	151/1972{	0.631403	1	0.932375	ENSG0000(	TRAPPC5/I	GO_MF
GO:00512I	5	5/1121	95/19728	0.633494	1	0.926241	ENSG0000(	KDM6B/ALI	GO_MF
GO:00611:	13	13/1121	247/1972{	0.652114	1	0.926241	ENSG0000(	CSTB/BST:	GO_MF
GO:00018{	22	22/1121	415/1972{	0.663228	1	0.932936	ENSG0000(	EEF1A1/TI	GO_MF
GO:00152:	5	5/1121	99/19728	0.669468	1	0.888817	ENSG0000(	SLC25A5/I	GO_MF
GO:00423{	11	11/1121	215/1972{	0.682889	1	0.900392	ENSG0000(	WDR5/PYG(	GO_MF
GO:00991(	6	6/1121	120/1972{	0.683443	1	0.879929	ENSG0000(	SRI/GNB2,	GO_MF
GO:00050{	1	1/1121	20/19728	0.68982	1	0.879929	ENSG0000(	SEC61B	GO_MF
GO:00162!	1	1/1121	20/19728	0.68982	1	0.879929	ENSG0000(	TAF10	GO_MF
GO:00165(	1	1/1121	20/19728	0.68982	1	0.879929	ENSG0000(	ADIPOR1	GO_MF
GO:00168!	1	1/1121	20/19728	0.68982	1	0.879929	ENSG0000(	DHRS9	GO_MF
GO:00316{	1	1/1121	20/19728	0.68982	1	0.879929	ENSG0000(	GNAI2	GO_MF
GO:00151⁄	2	2/1121	42/19728	0.697811	1	0.838027	ENSG0000(	AQP3/SLC:	GO_MF
GO:00047:	10	10/1121	199/1972{	0.700051	1	0.88435	ENSG0000(	CTDSP2/DI	GO_MF
GO:00166:	1	1/1121	21/19728	0.707463	1	0.838027	ENSG0000(	AOC3	GO_MF
GO:00708⁄	1	1/1121	21/19728	0.707463	1	0.838027	ENSG0000(	SNCA	GO_MF
GO:00016!	8	8/1121	162/1972{	0.707824	1	0.869065	ENSG0000(	FPR2/AGTI	GO_MF
GO:00515:	3	3/1121	64/19728	0.712472	1	0.824933	ENSG0000(	ISCU/RSAI	GO_MF
GO:00080:	10	10/1121	202/1972{	0.717485	1	0.871216	ENSG0000(	DYNLT1/M/	GO_MF
GO:00160I	1	1/1121	22/19728	0.724104	1	0.799935	ENSG0000(	PPIF	GO_MF
GO:00080I	4	4/1121	87/19728	0.735722	1	0.80913	ENSG0000(	KDM6B/SKI	GO_MF
GO:00051!	1	1/1121	23/19728	0.739798	1	0.765155	ENSG0000(	IRS2	GO_MF
GO:00168⁄	1	1/1121	23/19728	0.739798	1	0.765155	ENSG0000(	ADCY7	GO_MF
GO:00327{	1	1/1121	23/19728	0.739798	1	0.765155	ENSG0000(	RGS14	GO_MF
GO:01401(	1	1/1121	23/19728	0.739798	1	0.765155	ENSG0000(	TRMT112	GO_MF
GO:00306'	9	9/1121	187/1972{	0.741839	1	0.84699	ENSG0000(	SDCBP/LA!	GO_MF
GO:00431'	4	4/1121	88/19728	0.743847	1	0.799935	ENSG0000(	CDIPT/TSI	GO_MF
GO:00430:	2	2/1121	46/19728	0.744622	1	0.765155	ENSG0000(	SNCA/CASI	GO_MF
GO:00198:	7	7/1121	149/1972{	0.749007	1	0.826779	ENSG0000(	DUSP1/IL:	GO_MF
GO:00301!	1	1/1121	25/19728	0.768562	1	0.703943	ENSG0000(	RGS14	GO_MF
GO:00312I	1	1/1121	26/19728	0.78173	1	0.676868	ENSG0000(	CHMP2A	GO_MF
GO:00455(	1	1/1121	26/19728	0.78173	1	0.676868	ENSG0000(	GLUL	GO_MF
GO:00053:	1	1/1121	27/19728	0.794149	1	0.651799	ENSG0000(	SLC6A8	GO_MF
GO:00324!	1	1/1121	27/19728	0.794149	1	0.651799	ENSG0000(	KDM6B	GO_MF
GO:00082'	4	4/1121	95/19728	0.795444	1	0.740993	ENSG0000(	TRMT112/\	GO_MF
GO:00168I	6	6/1121	138/1972{	0.802806	1	0.765155	ENSG0000(	SIN3B/AMI	GO_MF
GO:00168:	3	3/1121	75/19728	0.806739	1	0.703943	ENSG0000(	APEX2/FH,	GO_MF
GO:00168(	1	1/1121	29/19728	0.816909	1	0.606847	ENSG0000(	TRUB1	GO_MF
GO:00432:	1	1/1121	30/19728	0.827328	1	0.586619	ENSG0000(	LGALS3	GO_MF
GO:00455(	1	1/1121	30/19728	0.827328	1	0.586619	ENSG0000(	BAG3	GO_MF
GO:00020:	3	3/1121	79/19728	0.833891	1	0.6683	ENSG0000(	BCL2L12/I	GO_MF
GO:01400:	7	7/1121	165/1972{	0.834295	1	0.746606	ENSG0000(	WDR5/ING:	GO_MF
GO:00167(	1	1/1121	31/19728	0.837155	1	0.567696	ENSG0000(	PTGS2	GO_MF
GO:00303:	1	1/1121	31/19728	0.837155	1	0.567696	ENSG0000(	CDKN1A	GO_MF
GO:00329⁄	1	1/1121	31/19728	0.837155	1	0.567696	ENSG0000(	RGS14	GO_MF
GO:00702'	2	2/1121	58/19728	0.84937	1	0.606847	ENSG0000(	MARC1/AL/	GO_MF
GO:00197{	21	21/1121	449/1972{	0.850213	1	0.823096	ENSG0000(	IRF2BPL/'	GO_MF
GO:00355{	3	3/1121	82/19728	0.852039	1	0.64385	ENSG0000(	LASP1/SH:	GO_MF
GO:00313(	1	1/1121	33/19728	0.855164	1	0.53329	ENSG0000(	HHEX	GO_MF
GO:00171'	9	9/1121	212/1972{	0.856946	1	0.747109	ENSG0000(	MMP9/CFD,	GO_MF
GO:00166:	2	2/1121	60/19728	0.862445	1	0.586619	ENSG0000(	SDHC/LBR	GO_MF
GO:00302'	2	2/1121	60/19728	0.862445	1	0.586619	ENSG0000(	BIN1/HGN(	GO_MF
GO:00170{	1	1/1121	34/19728	0.863407	1	0.517605	ENSG0000(	FXYD5	GO_MF
GO:00045:	2	2/1121	61/19728	0.868585	1	0.577002	ENSG0000(	APEX2/MU!	GO_MF

GO:00321ا	4	4/1121	109/1972ا	0.873412	1	0.645819	ENSG0000(	TOLLIP/FI	GO_MF
GO:00971	2	2/1121	63/19728	0.880115	1	0.558685	ENSG0000(	VIM/CDC3'	GO_MF
GO:00718	1	1/1121	37/19728	0.885431	1	0.475637	ENSG0000(	APOL2	GO_MF
GO:00167(	2	2/1121	65/19728	0.890704	1	0.541495	ENSG0000(	MGST3/AL(	GO_MF
GO:00162	1	1/1121	38/19728	0.891953	1	0.46312	ENSG0000(	ENSA	GO_MF
GO:00171	1	1/1121	38/19728	0.891953	1	0.46312	ENSG0000(	FZD2	GO_MF
GO:00904ا	1	1/1121	38/19728	0.891953	1	0.46312	ENSG0000(	SLC2A1	GO_MF
GO:00433	1	1/1121	39/19728	0.898105	1	0.451245	ENSG0000(	SDCBP	GO_MF
GO:00162	1	1/1121	42/19728	0.914538	1	0.419014	ENSG0000(	DHRS9	GO_MF
GO:01010(	4	4/1121	120/1972ا	0.915251	1	0.586619	ENSG0000(	ZC3H12A/I	GO_MF
GO:00169(	1	1/1121	44/19728	0.923994	1	0.399968	ENSG0000(	PDHB	GO_MF
GO:19016ا	1	1/1121	44/19728	0.923994	1	0.399968	ENSG0000(	SLC35B2	GO_MF
GO:00168'	1	1/1121	47/19728	0.936255	1	0.374438	ENSG0000(	GLUL	GO_MF
GO:00053	5	5/1121	156/1972ا	0.945981	1	0.564057	ENSG0000(	SLC16A5/	GO_MF
GO:00463	2	2/1121	83/19728	0.953602	1	0.424062	ENSG0000(	PURA/RGC(	GO_MF
GO:00055	1	1/1121	54/19728	0.95772	1	0.325899	ENSG0000(	PRELID1	GO_MF
GO:00198	4	4/1121	145/1972ا	0.968056	1	0.485478	ENSG0000(	FOLR3/MAI	GO_MF
GO:00017ا	1	1/1121	61/19728	0.971961	1	0.288501	ENSG0000(	MFGE8	GO_MF
GO:00166	4	4/1121	149/1972ا	0.972864	1	0.472445	ENSG0000(	DHRS9/HM(	GO_MF
GO:00081'	1	1/1121	69/19728	0.982469	1	0.255052	ENSG0000(	TRMT112	GO_MF
GO:00055	15	15/1121	443/1972ا	0.990884	1	0.595888	ENSG0000(	SDCBP/TWI	GO_MF
GO:00228(	17	17/1121	496/1972ا	0.992729	1	0.603177	ENSG0000(	PANX2/AQI	GO_MF
GO:00305	2	2/1121	129/1972ا	0.995458	1	0.272846	ENSG0000(	TSPO/GABI	GO_MF
GO:00082(	4	4/1121	193/1972ا	0.99591	1	0.364737	ENSG0000(	AAMP/HDGI	GO_MF
GO:00055	6	6/1121	252/1972ا	0.996463	1	0.419014	ENSG0000(	AAMP/HDGI	GO_MF
GO:00167(	3	3/1121	176/1972ا	0.997791	1	0.299976	ENSG0000(	CYP4F3/P	GO_MF
GO:00167	5	5/1121	240/1972ا	0.998174	1	0.366637	ENSG0000(	TRMT112/	GO_MF
GO:00044	1	1/1121	114/1972ا	0.998755	1	0.154373	ENSG0000(	CYP4F3	GO_MF
GO:00055	3	3/1121	212/1972ا	0.999628	1	0.249036	ENSG0000(	EEF1A1/R(	GO_MF
GO:00506(	6	6/1121	310/1972ا	0.999698	1	0.340618	ENSG0000(	LBR/CYB5I	GO_MF
