# Supplementary material for: Genomic Expression Profiling and Bioinformatics Analysis of Chronic Recurrent Multifocal Osteomyelitis
Source: Biomed Res Int. 2021 Feb 9;2021:6898093. doi: 10.1155/2021/6898093 (PMC7888306; doi:10.1155/2021/6898093)
Supplement: Supplementary 8 — Supplementary File 3 The detailed results of the KEGG pathway analyses of all the DEGs. [file 6898093.f8.pdf]

Pathway ID and Terms	Counts	GeneRati(	BgRatio	pValue
hsa00190~Oxidative phosphorylation	31	31/496	133/8037	6.50E-11
hsa03010~Ribosome	33	33/496	158/8037	3.50E-10
hsa05012~Parkinson disease	43	43/496	247/8037	3.51E-10
hsa04932~Non-alcoholic fatty liver disease (NAF	28	28/496	149/8037	8.79E-08
hsa05010~Alzheimer disease	45	45/496	334/8037	4.47E-07
hsa04145~Phagosome	27	27/496	152/8037	4.92E-07
hsa05016~Huntington disease	39	39/496	271/8037	5.02E-07
hsa04714~Thermogenesis	34	34/496	231/8037	1.65E-06
hsa05140~Leishmaniasis	17	17/496	77/8037	3.20E-06
hsa04612~Antigen processing and presentation	15	15/496	78/8037	6.82E-05
hsa05134~Legionellosis	12	12/496	57/8037	0.000147
hsa00510~N-Glycan biosynthesis	11	11/496	50/8037	0.000183
hsa05169~Epstein-Barr virus infection	26	26/496	201/8037	0.00025
hsa04723~Retrograde endocannabinoid signaling	21	21/496	148/8037	0.000276
hsa05132~Salmonella infection	26	26/496	214/8037	0.000667
hsa05152~Tuberculosis	23	23/496	180/8037	0.000669
hsa04141~Protein processing in endoplasmic reti	21	21/496	167/8037	0.001389
hsa05166~Human T-cell leukemia virus 1 infectic	25	25/496	219/8037	0.002047
hsa03060~Protein export	6	6/496	23/8037	0.002193
hsa05144~Malaria	9	9/496	50/8037	0.00312
hsa03040~Spliceosome	18	18/496	149/8037	0.004593
hsa05150~Staphylococcus aureus infection	13	13/496	96/8037	0.005833
hsa05145~Toxoplasmosis	14	14/496	112/8037	0.008751
hsa05162~Measles	16	16/496	138/8037	0.010723
hsa05110~Vibrio cholerae infection	8	8/496	50/8037	0.010759
hsa05323~Rheumatoid arthritis	12	12/496	93/8037	0.011518
hsa04662~B cell receptor signaling pathway	11	11/496	82/8037	0.011542
hsa04216~Ferroptosis	7	7/496	41/8037	0.011772
hsa04380~Osteoclast differentiation	15	15/496	128/8037	0.012076
hsa04213~Longevity regulating pathway - multipl	9	9/496	62/8037	0.013136
hsa04260~Cardiac muscle contraction	11	11/496	87/8037	0.017528
hsa05131~Shigellosis	23	23/496	236/8037	0.019394
hsa04210~Apoptosis	15	15/496	136/8037	0.020157
hsa00920~Sulfur metabolism	3	3/496	10/8037	0.020232
hsa05216~Thyroid cancer	6	6/496	37/8037	0.024333
hsa05330~Allograft rejection	6	6/496	38/8037	0.027442
hsa04666~Fc gamma R-mediated phagocytosis	11	11/496	93/8037	0.027455
hsa04657~IL-17 signaling pathway	11	11/496	94/8037	0.029438
hsa05416~Viral myocarditis	8	8/496	60/8037	0.030044
hsa00513~Various types of N-glycan biosynthesis	6	6/496	39/8037	0.030803
hsa04068~FoxO signaling pathway	14	14/496	131/8037	0.030836
hsa05164~Influenza A	17	17/496	170/8037	0.032917
hsa04217~Necroptosis	16	16/496	159/8037	0.035927
hsa05163~Human cytomegalovirus infection	21	21/496	225/8037	0.03735
hsa05170~Human immunodeficiency virus 1 infecti	20	20/496	212/8037	0.037707
hsa05210~Colorectal cancer	10	10/496	86/8037	0.038273
hsa05332~Graft-versus-host disease	6	6/496	41/8037	0.038306
hsa04330~Notch signaling pathway	7	7/496	53/8037	0.042949
hsa05320~Autoimmune thyroid disease	7	7/496	53/8037	0.042949
hsa05133~Pertussis	9	9/496	76/8037	0.043392
hsa04215~Apoptosis - multiple species	5	5/496	32/8037	0.044413
hsa05321~Inflammatory bowel disease (IBD)	8	8/496	65/8037	0.045468
hsa04940~Type I diabetes mellitus	6	6/496	43/8037	0.04688

hsa04142~Lysosome	13	13/496	128/8037	0.051742
hsa04144~Endocytosis	22	22/496	248/8037	0.053922
hsa03050~Proteasome	6	6/496	45/8037	0.056555
hsa04920~Adipocytokine signaling pathway	8	8/496	69/8037	0.060998
hsa04659~Th17 cell differentiation	11	11/496	107/8037	0.065128
hsa04672~Intestinal immune network for IgA proc	6	6/496	49/8037	0.079249
hsa04966~Collecting duct acid secretion	4	4/496	27/8037	0.081586
hsa04621~NOD-like receptor signaling pathway	16	16/496	181/8037	0.092611
hsa04064~NF-kappa B signaling pathway	10	10/496	104/8037	0.107429
hsa04625~C-type lectin receptor signaling pathw	10	10/496	104/8037	0.107429
hsa00020~Citrate cycle (TCA cycle)	4	4/496	30/8037	0.110528
hsa00030~Pentose phosphate pathway	4	4/496	30/8037	0.110528
hsa03018~RNA degradation	8	8/496	79/8037	0.112855
hsa05222~Small cell lung cancer	9	9/496	92/8037	0.113251
hsa05221~Acute myeloid leukemia	7	7/496	67/8037	0.117624
hsa04928~Parathyroid hormone synthesis, secreti	10	10/496	106/8037	0.117897
hsa03020~RNA polymerase	4	4/496	31/8037	0.121066
hsa05310~Asthma	4	4/496	31/8037	0.121066
hsa05161~Hepatitis B	14	14/496	162/8037	0.126095
hsa04962~Vasopressin-regulated water reabsorpti	5	5/496	44/8037	0.132585
hsa04371~Apelin signaling pathway	12	12/496	137/8037	0.138739
hsa04622~RIG-I-like receptor signaling pathway	7	7/496	70/8037	0.139162
hsa04668~TNF signaling pathway	10	10/496	112/8037	0.152538
hsa04670~Leukocyte transendothelial migration	10	10/496	113/8037	0.158766
hsa01230~Biosynthesis of amino acids	7	7/496	75/8037	0.178913
hsa03030~DNA replication	4	4/496	36/8037	0.179488
hsa04727~GABAergic synapse	8	8/496	89/8037	0.182481
hsa05235~PD-L1 expression and PD-1 checkpoint p	8	8/496	89/8037	0.182481
hsa01200~Carbon metabolism	10	10/496	117/8037	0.184889
hsa04218~Cellular senescence	13	13/496	160/8037	0.188275
hsa04062~Chemokine signaling pathway	15	15/496	189/8037	0.189578
hsa05217~Basal cell carcinoma	6	6/496	63/8037	0.191406
hsa05205~Proteoglycans in cancer	16	16/496	205/8037	0.19745
hsa04658~Th1 and Th2 cell differentiation	8	8/496	92/8037	0.206365
hsa04140~Autophagy - animal	11	11/496	137/8037	0.224635
hsa04066~HIF-1 signaling pathway	9	9/496	109/8037	0.229728
hsa04915~Estrogen signaling pathway	11	11/496	138/8037	0.231618
hsa04530~Tight junction	13	13/496	169/8037	0.243788
hsa04150~mTOR signaling pathway	12	12/496	155/8037	0.24842
hsa05160~Hepatitis C	12	12/496	155/8037	0.24842
hsa04923~Regulation of lipolysis in adipocytes	5	5/496	55/8037	0.250613
hsa05167~Kaposi sarcoma-associated herpesvirus	14	14/496	186/8037	0.25801
hsa05202~Transcriptional misregulation in cance	14	14/496	186/8037	0.25801
hsa05120~Epithelial cell signaling in Helicobac	6	6/496	70/8037	0.262383
hsa05130~Pathogenic Escherichia coli infection	15	15/496	202/8037	0.26485
hsa04010~MAPK signaling pathway	21	21/496	294/8037	0.272991
hsa00480~Glutathione metabolism	5	5/496	57/8037	0.274504
hsa05014~Amyotrophic lateral sclerosis (ALS)	5	5/496	57/8037	0.274504
hsa05203~Viral carcinogenesis	15	15/496	204/8037	0.277312
hsa00630~Glyoxylate and dicarboxylate metabolis	3	3/496	30/8037	0.281687
hsa04115~p53 signaling pathway	6	6/496	72/8037	0.283835
hsa04978~Mineral absorption	5	5/496	58/8037	0.286624
hsa05213~Endometrial cancer	5	5/496	58/8037	0.286624
hsa01524~Platinum drug resistance	6	6/496	73/8037	0.294703

hsa00052~Galactose metabolism	3	3/496	31/8037	0.298896
hsa04620~Toll-like receptor signaling pathway	8	8/496	104/8037	0.311921
hsa05226~Gastric cancer	11	11/496	149/8037	0.313221
hsa04930~Type II diabetes mellitus	4	4/496	46/8037	0.315331
hsa05135~Yersinia infection	9	9/496	120/8037	0.321764
hsa05212~Pancreatic cancer	6	6/496	76/8037	0.327756
hsa05220~Chronic myeloid leukemia	6	6/496	76/8037	0.327756
hsa00600~Sphingolipid metabolism	4	4/496	47/8037	0.3296
hsa00051~Fructose and mannose metabolism	3	3/496	33/8037	0.33344
hsa03410~Base excision repair	3	3/496	33/8037	0.33344
hsa04130~SNARE interactions in vesicular transp	3	3/496	33/8037	0.33344
hsa04623~Cytosolic DNA-sensing pathway	5	5/496	63/8037	0.348384
hsa00270~Cysteine and methionine metabolism	4	4/496	49/8037	0.35822
hsa05020~Prion diseases	3	3/496	35/8037	0.367913
hsa00500~Starch and sucrose metabolism	3	3/496	36/8037	0.385042
hsa05223~Non-small cell lung cancer	5	5/496	66/8037	0.385859
hsa04913~Ovarian steroidogenesis	4	4/496	51/8037	0.386818
hsa05143~African trypanosomiasis	3	3/496	37/8037	0.402062
hsa04640~Hematopoietic cell lineage	7	7/496	99/8037	0.411883
hsa03430~Mismatch repair	2	2/496	23/8037	0.419603
hsa04015~Rap1 signaling pathway	14	14/496	210/8037	0.422226
hsa05224~Breast cancer	10	10/496	147/8037	0.422355
hsa05230~Central carbon metabolism in cancer	5	5/496	69/8037	0.423219
hsa04916~Melanogenesis	7	7/496	101/8037	0.432427
hsa00534~Glycosaminoglycan biosynthesis - hepar	2	2/496	24/8037	0.441211
hsa05142~Chagas disease (American trypanosomias	7	7/496	102/8037	0.442666
hsa05146~Amoebiasis	7	7/496	102/8037	0.442666
hsa04071~Sphingolipid signaling pathway	8	8/496	119/8037	0.454325
hsa04935~Growth hormone synthesis, secretion ar	8	8/496	119/8037	0.454325
hsa04022~cGMP-PKG signaling pathway	11	11/496	167/8037	0.456748
hsa04540~Gap junction	6	6/496	88/8037	0.461909
hsa04014~Ras signaling pathway	15	15/496	232/8037	0.464661
hsa05225~Hepatocellular carcinoma	11	11/496	168/8037	0.464758
hsa05219~Bladder cancer	3	3/496	41/8037	0.468546
hsa04211~Longevity regulating pathway	6	6/496	89/8037	0.472915
hsa04950~Maturity onset diabetes of the young	2	2/496	26/8037	0.483048
hsa04918~Thyroid hormone synthesis	5	5/496	74/8037	0.48439
hsa04934~Cushing syndrome	10	10/496	155/8037	0.489692
hsa04910~Insulin signaling pathway	9	9/496	139/8037	0.490792
hsa05418~Fluid shear stress and atherosclerosis	9	9/496	139/8037	0.490792
hsa05032~Morphine addiction	6	6/496	91/8037	0.49473
hsa00062~Fatty acid elongation	2	2/496	27/8037	0.503229
hsa00130~Ubiquinone and other terpenoid-quinone	1	1/496	11/8037	0.503992
hsa04912~GnRH signaling pathway	6	6/496	93/8037	0.516236
hsa04310~Wnt signaling pathway	10	10/496	160/8037	0.530859
hsa04926~Relaxin signaling pathway	8	8/496	129/8037	0.546973
hsa04630~JAK-STAT signaling pathway	10	10/496	162/8037	0.547017
hsa00590~Arachidonic acid metabolism	4	4/496	63/8037	0.550716
hsa05168~Herpes simplex virus 1 infection	30	30/496	491/8037	0.552005
hsa00230~Purine metabolism	8	8/496	130/8037	0.555911
hsa03420~Nucleotide excision repair	3	3/496	47/8037	0.561408
hsa04650~Natural killer cell mediated cytotoxic	8	8/496	131/8037	0.564776
hsa04072~Phospholipase D signaling pathway	9	9/496	148/8037	0.567877
hsa04514~Cell adhesion molecules (CAMs)	9	9/496	148/8037	0.567877

hsa00564~Glycerophospholipid metabolism	6	6/496	98/8037	0.568338
hsa04137~Mitophagy - animal	4	4/496	65/8037	0.575832
hsa00520~Amino sugar and nucleotide sugar metal	3	3/496	48/8037	0.575908
hsa04136~Autophagy - other	2	2/496	32/8037	0.596175
hsa00330~Arginine and proline metabolism	3	3/496	50/8037	0.603993
hsa04340~Hedgehog signaling pathway	3	3/496	50/8037	0.603993
hsa05322~Systemic lupus erythematosus	8	8/496	136/8037	0.607873
hsa04610~Complement and coagulation cascades	5	5/496	85/8037	0.609218
hsa00010~Glycolysis / Gluconeogenesis	4	4/496	68/8037	0.611969
hsa04921~Oxytocin signaling pathway	9	9/496	154/8037	0.616374
hsa04152~AMPK signaling pathway	7	7/496	120/8037	0.616554
hsa05211~Renal cell carcinoma	4	4/496	69/8037	0.623584
hsa04922~Glucagon signaling pathway	6	6/496	106/8037	0.645548
hsa04110~Cell cycle	7	7/496	124/8037	0.651071
hsa03460~Fanconi anemia pathway	3	3/496	54/8037	0.656367
hsa04976~Bile secretion	4	4/496	72/8037	0.657088
hsa00360~Phenylalanine metabolism	1	1/496	17/8037	0.661771
hsa00450~Selenocompound metabolism	1	1/496	17/8037	0.661771
hsa00910~Nitrogen metabolism	1	1/496	17/8037	0.661771
hsa05100~Bacterial invasion of epithelial cells	4	4/496	73/8037	0.667799
hsa03013~RNA transport	10	10/496	180/8037	0.680867
hsa00511~Other glycan degradation	1	1/496	18/8037	0.682689
hsa01210~2-Oxocarboxylic acid metabolism	1	1/496	18/8037	0.682689
hsa05214~Glioma	4	4/496	75/8037	0.688528
hsa05340~Primary immunodeficiency	2	2/496	38/8037	0.689728
hsa04350~TGF-beta signaling pathway	5	5/496	94/8037	0.697141
hsa04971~Gastric acid secretion	4	4/496	76/8037	0.698544
hsa00531~Glycosaminoglycan degradation	1	1/496	19/8037	0.702316
hsa00770~Pantothenate and CoA biosynthesis	1	1/496	19/8037	0.702316
hsa00620~Pyruvate metabolism	2	2/496	39/8037	0.703461
hsa04724~Glutamatergic synapse	6	6/496	114/8037	0.713826
hsa00260~Glycine, serine and threonine metaboli	2	2/496	40/8037	0.716684
hsa04721~Synaptic vesicle cycle	4	4/496	78/8037	0.717878
hsa00100~Steroid biosynthesis	1	1/496	20/8037	0.720731
hsa00532~Glycosaminoglycan biosynthesis - chonc	1	1/496	20/8037	0.720731
hsa04713~Circadian entrainment	5	5/496	97/8037	0.723219
hsa03440~Homologous recombination	2	2/496	41/8037	0.72941
hsa01522~Endocrine resistance	5	5/496	98/8037	0.731546
hsa05231~Choline metabolism in cancer	5	5/496	98/8037	0.731546
hsa00561~Glycerolipid metabolism	3	3/496	61/8037	0.735625
hsa00220~Arginine biosynthesis	1	1/496	21/8037	0.738009
hsa04070~Phosphatidylinositol signaling system	5	5/496	99/8037	0.73969
hsa00860~Porphyrin and chlorophyll metabolism	2	2/496	42/8037	0.741647
hsa05165~Human papillomavirus infection	18	18/496	330/8037	0.742768
hsa04120~Ubiquitin mediated proteolysis	7	7/496	136/8037	0.742869
hsa04061~Viral protein interaction with cytokir	5	5/496	100/8037	0.747652
hsa04750~Inflammatory mediator regulation of TF	5	5/496	100/8037	0.747652
hsa04722~Neurotrophin signaling pathway	6	6/496	119/8037	0.751638
hsa04975~Fat digestion and absorption	2	2/496	43/8037	0.753408
hsa00900~Terpenoid backbone biosynthesis	1	1/496	22/8037	0.75422
hsa04810~Regulation of actin cytoskeleton	11	11/496	213/8037	0.772379
hsa03022~Basal transcription factors	2	2/496	45/8037	0.775551
hsa04012~ErbB signaling pathway	4	4/496	85/8037	0.77831
hsa04611~Platelet activation	6	6/496	124/8037	0.785693

hsa04024~cAMP signaling pathway	11	11/496	216/8037	0.78758
hsa00830~Retinol metabolism	3	3/496	67/8037	0.791441
hsa00514~Other types of O-glycan biosynthesis	2	2/496	47/8037	0.795937
hsa00563~Glycosylphosphatidylinositol (GPI)-anc	1	1/496	25/8037	0.797089
hsa04664~Fc epsilon RI signaling pathway	3	3/496	68/8037	0.799724
hsa04931~Insulin resistance	5	5/496	108/8037	0.804924
hsa04924~Renin secretion	3	3/496	69/8037	0.80773
hsa05030~Cocaine addiction	2	2/496	49/8037	0.81467
hsa04917~Prolactin signaling pathway	3	3/496	70/8037	0.815465
hsa01040~Biosynthesis of unsaturated fatty acic	1	1/496	27/8037	0.821436
hsa03015~mRNA surveillance pathway	4	4/496	91/8037	0.821554
hsa04970~Salivary secretion	4	4/496	91/8037	0.821554
hsa04520~Adherens junction	3	3/496	71/8037	0.822935
hsa04979~Cholesterol metabolism	2	2/496	50/8037	0.823448
hsa05218~Melanoma	3	3/496	72/8037	0.830146
hsa04744~Phototransduction	1	1/496	28/8037	0.832493
hsa00562~Inositol phosphate metabolism	3	3/496	74/8037	0.843815
hsa04726~Serotonergic synapse	5	5/496	115/8037	0.846145
hsa04961~Endocrine and other factor-regulated c	2	2/496	53/8037	0.847584
hsa00410~beta-Alanine metabolism	1	1/496	31/8037	0.86173
hsa01523~Antifolate resistance	1	1/496	31/8037	0.86173
hsa04710~Circadian rhythm	1	1/496	31/8037	0.86173
hsa05412~Arrhythmogenic right ventricular cardi	3	3/496	77/8037	0.862529
hsa04919~Thyroid hormone signaling pathway	5	5/496	119/8037	0.866291
hsa05206~MicroRNAs in cancer	15	15/496	310/8037	0.86981
hsa04933~AGE-RAGE signaling pathway in diabetic	4	4/496	100/8037	0.873176
hsa00983~Drug metabolism - other enzymes	3	3/496	79/8037	0.873885
hsa00240~Pyrimidine metabolism	2	2/496	57/8037	0.8751
hsa04550~Signaling pathways regulating pluripot	6	6/496	143/8037	0.883334
hsa04370~VEGF signaling pathway	2	2/496	59/8037	0.887073
hsa05204~Chemical carcinogenesis	3	3/496	83/8037	0.894135
hsa00310~Lysine degradation	2	2/496	61/8037	0.897976
hsa00250~Alanine, aspartate and glutamate metak	1	1/496	36/8037	0.899582
hsa00350~Tyrosine metabolism	1	1/496	36/8037	0.899582
hsa00760~Nicotinate and nicotinamide metabolism	1	1/496	37/8037	0.905807
hsa04911~Insulin secretion	3	3/496	86/8037	0.907356
hsa04927~Cortisol synthesis and secretion	2	2/496	65/8037	0.916902
hsa04270~Vascular smooth muscle contraction	5	5/496	132/8037	0.917066
hsa04728~Dopaminergic synapse	5	5/496	132/8037	0.917066
hsa05033~Nicotine addiction	1	1/496	40/8037	0.922265
hsa04725~Cholinergic synapse	4	4/496	113/8037	0.924813
hsa04720~Long-term potentiation	2	2/496	67/8037	0.92508
hsa04390~Hippo signaling pathway	6	6/496	157/8037	0.928573
hsa05031~Amphetamine addiction	2	2/496	69/8037	0.932495
hsa04360~Axon guidance	7	7/496	181/8037	0.936339
hsa05414~Dilated cardiomyopathy (DCM)	3	3/496	96/8037	0.941319
hsa02010~ABC transporters	1	1/496	45/8037	0.943566
hsa05215~Prostate cancer	3	3/496	97/8037	0.943991
hsa04925~Aldosterone synthesis and secretion	3	3/496	98/8037	0.94655
hsa04914~Progesterone-mediated oocyte maturatic	3	3/496	99/8037	0.949
hsa00565~Ether lipid metabolism	1	1/496	47/8037	0.950354
hsa04973~Carbohydrate digestion and absorption	1	1/496	47/8037	0.950354
hsa03320~PPAR signaling pathway	2	2/496	76/8037	0.95334
hsa04261~Adrenergic signaling in cardiomyocytes	5	5/496	149/8037	0.957488

hsa04660~T cell receptor signaling pathway	3	3/496	104/8037	0.959756
hsa01521~EGFR tyrosine kinase inhibitor resista	2	2/496	79/8037	0.960249
hsa04114~Oocyte meiosis	4	4/496	128/8037	0.960351
hsa04146~Peroxisome	2	2/496	83/8037	0.967951
hsa03008~Ribosome biogenesis in eukaryotes	3	3/496	111/8037	0.971285
hsa01212~Fatty acid metabolism	1	1/496	57/8037	0.973855
hsa04060~Cytokine-cytokine receptor interactior	11	11/496	294/8037	0.977119
hsa04730~Long-term depression	1	1/496	60/8037	0.978434
hsa04020~Calcium signaling pathway	6	6/496	193/8037	0.982308
hsa04929~GnRH secretion	1	1/496	64/8037	0.983319
hsa04510~Focal adhesion	6	6/496	199/8037	0.986201
hsa04151~PI3K-Akt signaling pathway	13	13/496	354/8037	0.987606
hsa04972~Pancreatic secretion	2	2/496	102/8037	0.988733
hsa00982~Drug metabolism - cytochrome P450	1	1/496	72/8037	0.990024
hsa00980~Metabolism of xenobiotics by cytochrom	1	1/496	77/8037	0.992767
hsa04742~Taste transduction	1	1/496	83/8037	0.995084
hsa04512~ECM-receptor interaction	1	1/496	88/8037	0.996437
hsa05410~Hypertrophic cardiomyopathy (HCM)	1	1/496	90/8037	0.996868
hsa05034~Alcoholism	4	4/496	187/8037	0.997566
hsa04974~Protein digestion and absorption	1	1/496	95/8037	0.997731
hsa04080~Neuroactive ligand-receptor interactic	7	7/496	340/8037	0.999932
hsa04740~Olfactory transduction	4	4/496	448/8037	1

FDR	foldEnrichment
1.89E-08	3.776786
3.41E-08	3.384302
3.41E-08	2.820875
6.40E-06	3.044977
2.09E-05	2.183124
2.09E-05	2.878276
2.09E-05	2.331888
5.99E-05	2.38495
0.000104	3.577425
0.001984	3.116083
0.003887	3.41129
0.004436	3.564798
0.005595	2.095992
0.005727	2.299164
0.012159	1.968665
0.012159	2.070464
0.023777	2.037582
0.033087	1.849729
0.03359	4.227034
0.045397	2.916653
0.06364	1.957485
0.077158	2.194241
0.110714	2.025454
0.121176	1.878682
0.121176	2.592581
0.121176	2.090791
0.121176	2.173658
0.121176	2.766473
0.121176	1.898863
0.12742	2.35214
0.164537	2.048735
0.173162	1.579167
0.173162	1.787165
0.173162	4.861089
0.202308	2.627616
0.215932	2.558468
0.215932	1.916558
0.218859	1.896169
0.218859	2.160484
0.218859	2.492866
0.218859	1.731686
0.22807	1.620363
0.237169	1.630554
0.237169	1.512339
0.237169	1.528644
0.237169	1.884143
0.237169	2.371263
0.252543	2.140102
0.252543	2.140102
0.252543	1.918851
0.253414	2.531817
0.254445	1.994293
0.2574	2.260971

0.278834	1.645681
0.285297	1.437419
0.293885	2.160484
0.311411	1.878682
0.326761	1.665794
0.39087	1.984118
0.395693	2.400538
0.441798	1.432365
0.491883	1.558041
0.491883	1.558041
0.491883	2.160484
0.491883	2.160484
0.491883	1.640874
0.491883	1.585138
0.496202	1.692916
0.496202	1.528644
0.496202	2.090791
0.496202	2.090791
0.509636	1.400314
0.528523	1.841321
0.53995	1.419296
0.53995	1.620363
0.584062	1.446753
0.600011	1.433949
0.655286	1.512339
0.655286	1.800403
0.655286	1.456506
0.655286	1.456506
0.655286	1.384926
0.655286	1.316545
0.655286	1.286002
0.655286	1.543203
0.668117	1.264673
0.690255	1.409011
0.742826	1.301021
0.748897	1.337914
0.748897	1.291594
0.775835	1.246433
0.775835	1.254475
0.775835	1.254475
0.775835	1.473057
0.782094	1.219628
0.782094	1.219628
0.786442	1.388882
0.786442	1.20324
0.786863	1.157402
0.786863	1.421371
0.786863	1.421371
0.786863	1.191443
0.786863	1.620363
0.786863	1.350302
0.786863	1.396865
0.786863	1.396865
0.801483	1.331805

0.805359	1.568093
0.822296	1.246433
0.822296	1.196241
0.822296	1.409011
0.822296	1.215272
0.822296	1.279234
0.822296	1.279234
0.822296	1.379032
0.822296	1.473057
0.822296	1.473057
0.822296	1.473057
0.851929	1.286002
0.868683	1.322745
0.884814	1.388882
0.907775	1.350302
0.907775	1.227548
0.907775	1.270873
0.935999	1.313808
0.947359	1.145711
0.947359	1.409011
0.947359	1.080242
0.947359	1.102288
0.947359	1.174176
0.960582	1.123024
0.961312	1.350302
0.961312	1.112014
0.961312	1.112014
0.966032	1.08932
0.966032	1.08932
0.966032	1.067305
0.966032	1.104793
0.966032	1.047648
0.966032	1.060952
0.966999	1.185631
0.969144	1.092379
0.971567	1.246433
0.971567	1.09484
0.971567	1.045395
0.971567	1.049156
0.971567	1.049156
0.972747	1.068371
0.977744	1.200269
0.977744	1.473057
0.994866	1.045395
1	1.012727
1	1.004876
1	1.000224
1	1.028802
1	0.990038
1	0.997146
1	1.034274
1	0.989535
1	0.985356
1	0.985356

1	0.992059
1	0.997146
1	1.012727
1	1.012727
1	0.972218
1	0.972218
1	0.953155
1	0.953155
1	0.953155
1	0.946965
1	0.945212
1	0.939341
1	0.917187
1	0.914721
1	0.900202
1	0.900202
1	0.953155
1	0.953155
1	0.953155
1	0.88787
1	0.900202
1	0.900202
1	0.900202
1	0.864194
1	0.852823
1	0.861895
1	0.852823
1	0.852823
1	0.852823
1	0.830955
1	0.852823
1	0.810181
1	0.830955
1	0.810181
1	0.810181
1	0.835239
1	0.790421
1	0.826716
1	0.826716
1	0.7969
1	0.771601
1	0.818365
1	0.771601
1	0.883834
1	0.83401
1	0.810181
1	0.810181
1	0.81699
1	0.753657
1	0.736529
1	0.836807
1	0.720161
1	0.762524
1	0.784047

1	0.825185
1	0.725536
1	0.689516
1	0.648145
1	0.714866
1	0.750168
1	0.704506
1	0.661373
1	0.694441
1	0.600134
1	0.712247
1	0.712247
1	0.68466
1	0.648145
1	0.675151
1	0.578701
1	0.656904
1	0.704506
1	0.611458
1	0.522698
1	0.522698
1	0.522698
1	0.63131
1	0.680825
1	0.784047
1	0.648145
1	0.615328
1	0.568548
1	0.679873
1	0.549276
1	0.585673
1	0.531267
1	0.450101
1	0.450101
1	0.437936
1	0.565243
1	0.498573
1	0.613774
1	0.613774
1	0.405091
1	0.57358
1	0.48369
1	0.619247
1	0.46967
1	0.62666
1	0.506363
1	0.360081
1	0.501143
1	0.496029
1	0.491019
1	0.344758
1	0.344758
1	0.426411
1	0.543746

1	0.467412
1	0.410218
1	0.506363
1	0.390449
1	0.437936
1	0.284274
1	0.606258
1	0.27006
1	0.50374
1	0.253182
1	0.488552
1	0.595049
1	0.317718
1	0.22505
1	0.210437
1	0.195224
1	0.184132
1	0.18004
1	0.346602
1	0.170565
1	0.333604
1	0.144675
